# Uniform Lipschitz Functions on the Triangular Lattice Have Logarithmic Variations

**DOI:** 10.1007/s00220-020-03920-z

**Published:** 2021-01-07

**Authors:** Alexander Glazman, Ioan Manolescu

**Affiliations:** 1 Faculty of Mathematics, University of Vienna, Oskar-Morgenstern-Platz 1, 1090 Vienna, Austria; 2 Département de Mathématiques, Université de Fribourg, 23 Chemin du Musée, 1700 Fribourg, Switzerland

## Abstract

Uniform integer-valued Lipschitz functions on a domain of size *N* of the triangular lattice are shown to have variations of order $\sqrt{\log N}$. The level lines of such functions form a loop *O*(2) model on the edges of the hexagonal lattice with edge-weight one. An infinite-volume Gibbs measure for the loop *O*(2) model is constructed as a thermodynamic limit and is shown to be unique. It contains only finite loops and has properties indicative of scale-invariance: macroscopic loops appearing at every scale. The existence of the infinite-volume measure carries over to height functions pinned at the origin; the uniqueness of the Gibbs measure does not. The proof is based on a representation of the loop *O*(2) model via a pair of spin configurations that are shown to satisfy the FKG inequality. We prove RSW-type estimates for a certain connectivity notion in the aforementioned spin model.

## Introduction

Height functions occupy a central role in statistical mechanics models on lattices. Indeed, the Ising, six-vertex and dimer models are only some of the lattice models involving height function representations. The predicted conformal invariance of these models is tightly linked to the convergence of their associated height functions to the Gaussian Free Field (GFF) or variations of it. Both statements were proved only in a handful of cases, and remain fascinating conjectures in general.

**Fig. 1:**
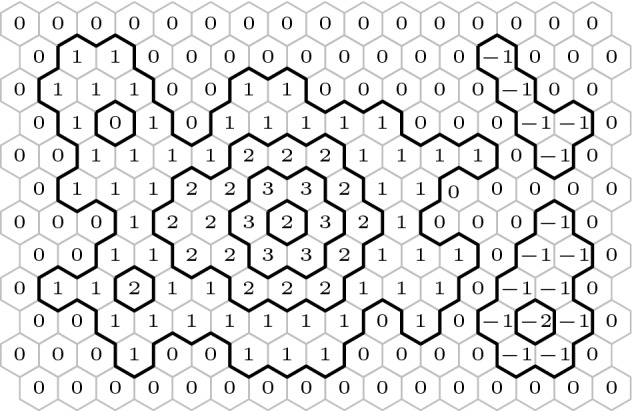
Lipschitz function — values at any two adjacent faces differ by at most 1. Level lines are shown in bold; they form a loop configuration distributed according to the loop *O*(2) measure with edge-weight one

In this paper we study integer-valued height functions defined on the vertices of the two-dimensional triangular lattice ${\mathbb {T}}$, or equivalently on the faces of the hexagonal lattice ${\mathbb {H}}$. It is then natural to impose that height functions are Lipschitz, that is functions whose difference between any two adjacent vertices is at most 1; see Figure 1. More specifically, for any finite domain ${\mathscr {D}}$ of ${\mathbb {H}}$, we will consider a uniformly chosen Lipschitz function among those with values 0 outside of ${\mathscr {D}}$. The question of interest is the behaviour of such a function, especially as the domain ${\mathscr {D}}$ increases towards ${\mathbb {H}}$.

Our goal is to show that the variance of the value at the origin of a uniformly chosen Lipschitz function is of order $\log N$, where *N* is the radius of the largest ball centred at the origin and contained in ${\mathscr {D}}$. This result, termed *delocalisation* (or logarithmic delocalisation to be precise) is in agreement with the conjectural convergence of uniform Lipschitz functions to the GFF.

The essential tool here is a re-interpretation of the uniform Lipschitz functions as the loop *O*(2) model, which in turn is represented as the superposition of two site percolations on ${\mathbb {T}}$ interacting with each other – below we view these as ±-spin assignments. This *double-spin representation* is obtained by colouring the loops of the loop *O*(2) model in two colours (as was done in [7]), then deriving a spin configuration from the families of loops of each colour. This may be viewed as the infinite-coupling-limit of the Ashkin–Teller model on the triangular lattice [26].

The loop *O*(*n*) model is defined on collections of non-intersecting simple cycles (loops) on a finite domain ${\mathscr {D}}$ of ${\mathbb {H}}$ and has two real parameters $n,x>0$. The probability of each configuration is proportional to *n* to the number of loops times *x* to the number of edges in it. The loop *O*(*n*) model has a rich conjectural phase diagram [4, 31] that remains mostly open; see [35] for an overview of the topic.

For $n = 2$, the model is expected to exhibit macroscopic loops when $x\ge \tfrac{1}{\sqrt{2}}$ and exponential decay of loop sizes when $x< \tfrac{1}{\sqrt{2}}$. The former is confirmed in this paper for $x = 1$ and in [13] for $x=\tfrac{1}{\sqrt{2}}$. The latter behaviour is shown to hold for $x < \tfrac{1}{\sqrt{3}}+\epsilon$ for some $\epsilon > 0$ in [21]. The correspondence between the loop *O*(2) model and Lipschitz functions holds for any $x > 0$, but the corresponding height functions are not uniform: they are weighted by *x* to the number of pairs of adjacent faces of ${\mathbb {H}}$ having different values. The regime of exponential decay of loop sizes corresponds to localisation for the height function; that of macroscopic loops corresponds to logarithmic delocalisation.

A main difficulty in the study of the loop *O*(*n*) model is the lack of monotonicity and positive association. These type of properties are however expected to hold in convenient representations of the model, as illustrated by the present paper and by [13]. Indeed, a core ingredient of our arguments is the FKG inequality, which we show for the marginals of the double-spin representation. We then develop Russo–Seymour–Welsh (or RSW)-type results for these marginals, which translate to similar statements for the loop and height function models.

The RSW theory was initially developed for percolation [36, 38], and later generalised to other models via more robust arguments (see for instance [3, 10, 11, 17, 42]). It has become increasingly clear that for the latter type of arguments to apply the essential feature of the model is an instance of the FKG inequality. Indeed, other restrictions such as independence, symmetries and planarity have been, in some forms, relaxed in recent works. In this paper we do yet another step towards generalising this approach by considering a case where the Spatial Markov property applies only in a limited way.

The FKG inequality mentioned above extends to the case of certain non-uniform distributions on Lipschitz functions (corresponding to the loop *O*(2) model with $x<1$) and more generally to the loop *O*(*n*) model with $n\ge 2, x\le \tfrac{1}{\sqrt{n-1}}$. Thus, we hope that this instance of the FKG inequality, together with the strategy of our proofs can be useful in other studies of the loop *O*(*n*) model.

Finally, we want to emphasise that in this work we do not attempt to prove convergence to the GFF. In general, the RSW theory can be viewed as a robust technique based on geometric constructions, but is not expected to lead to subtle convergence results. Indeed the seminal proofs of convergence of [8, 9, 27, 37, 40, 41] are all based on some form of exact solvability, which is missing in our case.

### Uniform Lipschitz functions

Let ${\mathbb {H}}$ denote the hexagonal lattice, embedded in ${\mathbb {R}}^2$ with the origin 0 being the center of a face and the distance between the centres of any adjacent faces being 1. Write $F({\mathbb {H}})$ for the set of faces of ${\mathbb {H}}$. A subgraph ${\mathscr {D}}= (V({\mathscr {D}}),E({\mathscr {D}}))$ of ${\mathbb {H}}$ without isolated vertices is called a *domain* if there exists a self-avoiding polygon in ${\mathbb {H}}$ denoted by $\partial _E{\mathscr {D}}$ such that $E({\mathscr {D}})$ is the set of edges surrounded by $\partial _E{\mathscr {D}}$ (excluding those of $\partial _E{\mathscr {D}}$). Denote by $F({\mathscr {D}})$ the set of faces adjacent to at least one edge of ${\mathscr {D}}$. The inner (and outer) face boundary of ${\mathscr {D}}$, written $\partial _\mathrm {in}{\mathscr {D}}$ (and $\partial _\mathrm {out}{\mathscr {D}}$, respectively) is the set of faces of ${\mathscr {D}}$ (and ${\mathbb {H}}\setminus {\mathscr {D}}$, respectively) bounded by at least one edge in $\partial _E {\mathscr {D}}$. The faces strictly in the interior of ${\mathscr {D}}$ are $\mathrm {Int}({\mathscr {D}}) = F({\mathscr {D}}) \setminus \partial _\mathrm {in}{\mathscr {D}}$.

For a domain ${\mathscr {D}}$, a Lipschitz function on ${\mathscr {D}}$ with zero boundary conditions is an integer-valued function $\phi$ on the faces of ${\mathscr {D}}$ with the constraint that

- if $u,v \in F({\mathscr {D}})$ are two adjacent faces, then $|\phi (u) - \phi (v)|\le 1$;
- for each $u\in \partial _\mathrm {in}{\mathscr {D}}$ we have $\phi (u) = 0$.

Since ${\mathscr {D}}$ is finite, only finitely many such functions exist. Write $\pi _{\mathscr {D}}$ for the uniform measure on such functions, and let $\Phi _{\mathscr {D}}$ denote a random variable with law $\pi _{\mathscr {D}}$.

#### Theorem 1.1

 

(i) There exist constants $c, C > 0$ such that, for any finite domain ${\mathscr {D}}$ with $0 \in {\mathscr {D}}$,
  $$\begin{aligned} c \, \log \mathrm {dist}(0,{\mathscr {D}}^c) \le \mathrm {Var}(\Phi _{\mathscr {D}}(0)) \le C\,\log \mathrm {dist}(0,{\mathscr {D}}^c). \end{aligned}$$
(ii) For any increasing sequence of domains $({\mathscr {D}}_n)_{n\ge 1}$ with $0 \in {\mathscr {D}}_1$ and ${\mathbb {H}}= \bigcup _n {\mathscr {D}}_n$, the sequence of variables $\Phi _{{\mathscr {D}}_n} - \Phi _{{\mathscr {D}}_n}(0)$ converges in law as $n \rightarrow \infty$ to a random Lipschitz function $\Phi _{\mathbb {H}}: {\mathbb {H}}\rightarrow {\mathbb {Z}}$ that is equal to 0 at 0. Write $\pi _{\mathbb {H}}$ for the law of $\Phi _{\mathbb {H}}$.
(iii) There exists $c,C> 0$ such that, for any distinct $x,y \in F({\mathbb {H}})$,
  $$\begin{aligned} c\log |x- y|\le \mathrm {Var}(\Phi _{\mathbb {H}}(x) - \Phi _{\mathbb {H}}(y)) \le C\log |x- y|. \end{aligned}$$
  The same holds for $\Phi _{\mathscr {D}}$ for any domain ${\mathscr {D}}$ containing the ball of radius $2|x- y|$ around *x*.

One may wish to study height functions with different values imposed on the boundary via so-called boundary conditions. While we do not attempt to provide the most general form of our result, let us briefly mention some direct generalisations. First, for constant boundary conditions – that is if we study uniform height functions with $\phi (u) = c$ for all $u\in \partial _\mathrm {in}{\mathscr {D}}$ – the law obtained is that of $c + \Phi _{\mathscr {D}}$, and the results above adapt readily. Versions of the results above may also be deduced for “flat” boundary conditions, that is boundary conditions whose maximum and minimum differ by at most a constant, independently of ${\mathscr {D}}$. The results for such boundary conditions may be obtained using the FKG inequality for the height function; we refer the reader to the upcoming paper [15] for formulations and proofs of such results in a slightly different context.

In addition to the theorem above, RSW-type statements may be proved for  $\Phi _{\mathscr {D}}$, see Theorem 5.6. These may be used to prove bounds on the tail of $\frac{1}{\sqrt{ \log N}}\Phi _{\mathscr {D}}(0)$ in a domain where $\mathrm {dist}(0,{\mathscr {D}}^c) =N$.

To the best of our knowledge this is the first instance when a uniformly distributed Lipschitz function is proven to have logarithmically diverging variance. Previously known results establish that the variance is bounded (referred to as *localisation*) in high dimensions [32], or when the underlying graph is a tree [33] or an expander [34]. The conjectured convergence of the height function to the GFF indicates that localisation should also hold on lattices in dimensions three and above.

Recently it was established in [13] that the variance is logarithmic in a very similar setup — also on the hexagonal lattice, though the distribution is not uniform but instead the probability of a function $\phi$ is proportional to $(1/\sqrt{2})^{\#\{u\sim v:\phi (u) \ne \phi (v)\}}$. This result follows from [13, Thm. 1] for $n=2$.

On the square lattice ${\mathbb {Z}}^2$, one may also consider the related model of graph homomorphisms from ${\mathbb {Z}}^2$ to ${\mathbb {Z}}$, which are defined as functions on the faces of ${\mathbb {Z}}^2$ restricted to differ by *exactly* one between any two adjacent faces. These functions may be viewed as height functions of the six-vertex model that has parameters $a,b,c>0$. When $a=b=1$ and $c>0$ is general, the height functions are weighted by $c^{n_5+n_6}$, where $n_5+n_6$ is the number of vertices of ${\mathbb {Z}}^2$ for which the four adjacent faces contain only two values. For the uniform model $c=1$ (termed square ice) a non-quantitative delocalisation result is proved in [6] based on an approach described in [39]. In [14] a dichotomy theorem similar to our Theorem 4.1 is developed and logarithmic delocalisation is shown. In [22] logarithmic delocalisation at $c=2$ and localisation for $c>2$ are shown, based on the Baxter–Kelland–Wu coupling [2] with the random-cluster model and results of [17] and [12], where the order of the phase transition in the latter model is computed. In the upcoming [15], the logarithmic delocalisation result is generalised to all $c \in [1,2]$.

Convergence of the height function of the dimer model to the GFF was proven in a seminal work by Kenyon [27] and was recently extended to the case of a weak interaction [20]. On the square lattice, this corresponds to graph homomorphisms to ${\mathbb {Z}}$ with $c \approx \sqrt{2}$. Proving convergence of delocalised discrete-valued height functions outside of the free-fermion solution remains a major open problem.

The case of real-valued height functions is better understood. In particular, convergence to the GFF was established for uniformly convex symmetric potentials (under additional regularity assumptions) [29] and the delocalisation was proven for some non-convex nearest-neighbour potentials [30].

### The loop *O*(2) model

Let ${\mathscr {D}}$ be a domain of ${\mathbb {H}}$. A *loop configuration on* ${\mathscr {D}}$ is a subgraph of ${\mathscr {D}}$ in which every vertex has even degree. Thus, a loop configuration is a disjoint union of loops (i.e., subgraphs which are isomorphic to cycles) that are contained entirely in ${\mathscr {D}}$. In particular, none of these loops contain edges of $\partial _E{\mathscr {D}}$. Denote by ${\mathscr {L}}({\mathscr {D}})$ the set of all loop configurations on ${\mathscr {D}}$.

The loop *O*(2) model on ${\mathscr {D}}$ with edge-weight 1 (and empty boundary conditions) is the measure ${\mathbb {P}}_{{\mathscr {D}}}$ on ${\mathscr {L}}({\mathscr {D}})$ given by

$$\begin{aligned} {\mathbb {P}}_{{\mathscr {D}}}(\omega ) = \frac{1}{Z({\mathscr {D}})}\,2^{\ell (\omega )}, \end{aligned}$$

where $\ell (\omega )$ is the number of loops in $\omega$. The normalising constant $Z({\mathscr {D}})$, chosen so that ${\mathbb {P}}_{{\mathscr {D}}}$ is a probability measure, is called the partition function.

**Fig. 2:**
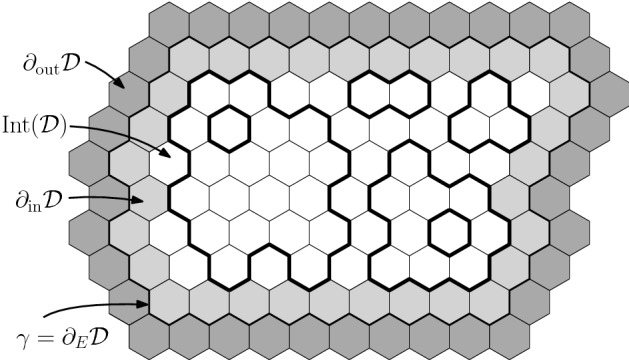
A loop configuration in a domain ${\mathscr {D}}$ bounded by the path $\gamma$. The domain ${\mathscr {D}}$ is formed of all the edges *strictly* in the interior of $\gamma$. Loops inside ${\mathscr {D}}$ are contained in the interior of $\gamma$ and are not allowed to intersect $\gamma$. The hexagons or $\partial _{\mathrm {in}} {\mathscr {D}}$ and $\partial _{\mathrm {out}}{\mathscr {D}}$ are marked by light and dark gray, respectively

Write $\Lambda _n$ for the domain defined by a self-avoiding contour going around the set of faces at distance *n* from 0 (for the graph distance on the dual ${\mathbb {H}}^*={\mathbb {T}}$ of ${\mathbb {H}}$). A sequence of domains $({\mathscr {D}}_n)_{n\ge 1}$ is said to converge to ${\mathbb {H}}$ if, for all *k*, all except finitely many domains of $({\mathscr {D}}_n)_{n\ge 1}$ contain $\Lambda _k$.

#### Theorem 1.2

(Existence of Gibbs measure and delocalisation). 

(i) For any increasing sequence of domains $({\mathscr {D}}_n)_{n\ge 1}$ converging to ${\mathbb {H}}$, ${\mathbb {P}}_{{\mathscr {D}}_n}$ has a limit denoted by ${\mathbb {P}}_{\mathbb {H}}$.
(ii) The measure ${\mathbb {P}}_{{\mathbb {H}}}$ is supported on even subgraphs of ${\mathbb {H}}$ that contain only finite loops.
(iii) The measure ${\mathbb {P}}_{{\mathbb {H}}}$ is ergodic and invariant under translations and rotations by $\pi /3$.
(iv) There exists $c > 0$ such that, for any even integer *n* and any finite domain ${\mathscr {D}}$ containing $\Lambda _{n}$, or for ${\mathscr {D}}= {\mathbb {H}}$,
  1.1 $$\begin{aligned} {\mathbb {P}}_{{\mathscr {D}}}(\text {there exists a loop in }\Lambda _{n}\text { surrounding }\Lambda _{n/2} )&\ge c. \end{aligned}$$
  Moreover, there exists $\rho < 1$, such that, for any finite domain ${\mathscr {D}}$, if we set $n = \mathrm {dist} (0,\partial _E {\mathscr {D}})$, we have
  1.2 $$\begin{aligned} {\mathbb {P}}_{{\mathscr {D}}}(\text {there exist two loops surrounding }\Lambda _{\rho n})&\le 1 - c. \end{aligned}$$
(v) Write $N_{\mathscr {D}}$ for the number of loops surrounding 0, contained in some domain ${\mathscr {D}}$. There exist constants $c,C >0$ such that for any domain ${\mathscr {D}}$,
  1.3 $$\begin{aligned} c \, \log \mathrm {dist}(0,{\mathscr {D}}^c) \le {\mathbb {E}}_{{\mathscr {D}}}(N_{\mathscr {D}}) \le C \, \log \mathrm {dist}(0,{\mathscr {D}}^c), \end{aligned}$$
  where ${\mathbb {E}}_{\mathscr {D}}$ denotes the expectation with respect to ${\mathbb {P}}_{\mathscr {D}}$. The same holds if we replace ${\mathbb {E}}_{\mathscr {D}}(N_{\mathscr {D}})$ with ${\mathbb {E}}_{\mathbb {H}}(N_{\mathscr {D}})$. In particular, ${\mathbb {P}}_{\mathbb {H}}$-a.s., there are infinitely many loops surrounding the origin.

Any limit of measures of the type ${\mathbb {P}}_{{\mathscr {D}}}$ is supported on even subgraphs of ${\mathbb {H}}$. Such graphs are in general disjoint unions of loops and infinite paths on ${\mathbb {H}}$. Thus, point *(ii)* of the above states that no infinite path exists ${\mathbb {P}}_{\mathbb {H}}$-a.s.

Point *(iv)* of the theorem above resembles a RSW-type statement for the loops of the *O*(2) model; indeed, it stems from an actual RSW result for a related model (see Corollary 5.2). Due to the imperfect correspondence between the models, the upper bound of (1.2) takes this slightly odd form. We believe that a similar bound should apply to any $\rho < 1$ (with *c* depending on $\rho$), for any *n* and for a single loop instead of two. This statement is of an independent interest as it would in particular imply, via Aizenman–Burchard [1], tightness of interfaces under Dobrushin 0/1 boundary conditions.

Point *(v)* is a direct consequence of *(iv)*. Moreover, bounds on the deviation of $N_{\mathscr {D}}$ from $\log \mathrm {dist}(0,{\mathscr {D}}^c)$ may be obtained in a straightforward manner.

Finally, we discuss the issue of Gibbs measures for the loop *O*(2) model. Consider a measure $\eta$ on $\{0,1\}^{{\mathbb {H}}}$ supported on even configurations. Recall that these are disjoint unions of bi-infinite paths and finite loops. Let $\omega$ be a configuration in the support of $\eta$ and ${\mathscr {D}}$ be a finite domain. Then $\omega \cap {\mathscr {D}}^c$ induces certain connections between the vertices of $\partial _E{\mathscr {D}}$. Indeed, each such vertex may be connected to another such vertex, to infinity, or be isolated. These connections constitute a boundary condition on ${\mathscr {D}}$. Formally we describe boundary conditions as follows.

For $\xi _1$ and $\xi _2$ two restrictions to ${\mathscr {D}}^c$ of even configurations on ${\mathbb {H}}$, write $\xi _1 \sim \xi _2$ if they induce the same connections on $\partial _E {\mathscr {D}}$. A measure $\eta$ on even configurations on ${\mathbb {H}}$ is called a Gibbs measure for the loop *O*(2) model with edge weight 1 if, for any finite domain ${\mathscr {D}}$ of ${\mathbb {H}}$ and any restriction $\xi$ of an even configuration to ${\mathscr {D}}^c$,

DLR $$\begin{aligned} \eta \big (\omega \cap {\mathscr {D}}= \omega _0 \,\big |\, \omega \cap {\mathscr {D}}^c \sim \xi \big ) = \frac{1}{Z_{\mathscr {D}}^{\xi }} \,2^{\#\text {finite loops of }\omega _0 \cup \xi \text { that intersect }{\mathscr {D}}} \, {\mathbf {1}}_{\{\omega _0 \cup \xi \text { is even}\}} \end{aligned}$$

for all $\omega _0 \in \{0,1\}^{E({\mathscr {D}})}$. The above equation needs only to hold when the conditioning is not degenerated. Write ${\mathbb {P}}_{\mathscr {D}}^{\xi }$ for the measure on $\{0,1\}^{E({\mathscr {D}})}$ described by the right-hand side above; it is the loop *O*(2) measure on ${\mathscr {D}}$ with boundary conditions $\xi$. It is immediate that ${\mathbb {P}}^\xi _{\mathscr {D}}$ does not depend on the choice of $\xi$ within its equivalency class for $\sim$.

Notice that the infinite paths do not contribute to the right-hand side of (DLR). One may be tempted to add a term of the form $(n')^{\#\text {infinite paths of }\omega _0 \cup \xi _0\text { that intersect }{\mathscr {D}}}$ in (DLR) for some $n' >0$. This would be superfluous, as the number of infinite paths intersecting ${\mathscr {D}}$ is imposed by the boundary conditions.

#### Theorem 1.3

(Uniqueness of Gibbs measure). There exists only one Gibbs measure for the loop *O*(2) model on ${\mathbb {H}}$ with edge-weight 1, namely ${\mathbb {P}}_{{\mathbb {H}}}$.

In particular, any Gibbs measure is supported on configurations formed entirely of finite loops. Notice that we do not require that the Gibbs measure be translation invariant or ergodic for it to be equal to ${\mathbb {P}}_{\mathbb {H}}$. However, we do not claim that for any sequence of domains ${\mathscr {D}}_n$ converging to ${\mathbb {H}}$ and any sequence of boundary conditions $\xi _n$ on these domains, ${\mathbb {P}}_{{\mathscr {D}}_n}^{\xi _n}$ tends to ${\mathbb {P}}_{\mathbb {H}}$. This is a stronger statement than Theorem 1.3; we believe it to be true, but have no proof. It may appear surprising, but limits of measures ${\mathbb {P}}_{{\mathscr {D}}_n}^{\xi _n}$ need not be Gibbs in the sense of (DLR). Theorem 1.3 does not imply the uniqueness of the Gibbs measure for height functions. We will discuss more on this point in the following section.

The loop model studied here is part of the larger class of loop *O*(*n*) models with edge weight *x*, where *n* and *x* are positive parameters. The loop *O*(*n*) model with edge-weight *x* in a domain ${\mathscr {D}}$ is the measure on loop configuration given by

$$\begin{aligned} {\mathbb {P}}_{{\mathscr {D}},n,x}(\omega ) = \frac{1}{Z({\mathscr {D}},n,x)}\,x^{|\omega |}n^{\ell (\omega )}, \end{aligned}$$

where $|\omega |$ is the number of edges in $\omega$ and $Z({\mathscr {D}},n,x)$ is called the partition function.

Results similar to Theorems 1.2 and 1.3 were proved in [13, Theorems 1 and 2] for the loop *O*(*n*) model with $n \in [1,2]$ and $x = \frac{1}{\sqrt{2 + \sqrt{2-n}}}$. They are based on the (single) spin representation of the loop *O*(*n*) model, which is shown to satisfy the FKG inequality for $n\ge 1$ and $x\le 1/\sqrt{n}$. This is then used to prove a dichotomy similar to our Theorem 4.1. For $x = \frac{1}{\sqrt{2 + \sqrt{2-n}}}$ and $n \in [1,2]$, the parafermionic observable is then used to exclude exponential decay of loops, thus proving the equivalent of Theorem 1.2. The uniqueness of the Gibbs measure is shown via the stronger statement which we are unable to prove here: convergence to the unique infinite-volume measure of finite-volume measures on any increasing sequence of domains, with any boundary conditions.

The point $n=2$, $x=1$ is clearly outside of the FKG regime determined in [13], and a more complicated spin representation is required. This representation will involve two spin configurations, and will therefore be sometimes referred to as the double-spin representation (see Section 2 for precise definitions).

Let us also mention that [16] proves that for *n* large enough and any $x >0$, the loops of the loop *O*(*n*) model with edge-weight *x* exhibit exponential decay. Moreover, for $n\,x^6$ large enough, it is shown that at least three distinct, linearly independent infinite-volume Gibbs measures exist. For $n\,x^6$ small enough (and *n* large) it was shown in the same paper that at least one Gibbs measure exists, but its uniqueness (though expected) was not proved.

### Relation between the loop *O*(2) model and random Lipschitz functions

Fix a domain ${\mathscr {D}}$. For a Lipschitz function $\varphi$ on ${\mathscr {D}}$, define an edge configuration $\omega _\varphi$ by $\omega _\varphi (e) = 1$ if and only if the two faces separated by *e* have different values of $\varphi$. It is straightforward to check that $\omega _\varphi$ is indeed a loop configuration.

#### Proposition 1.4

(i) If $\Phi$ has law $\pi _{\mathscr {D}}$, then $\omega _\Phi$ has law ${\mathbb {P}}_{\mathscr {D}}$.
(ii) Given some loop configuration $\omega$, the law of $\Phi$ conditionally on $\omega _\Phi = \omega$ is obtained as follows: define $\overrightarrow{\omega }$ by choosing a clockwise or a counter-clockwise orientation uniformly and independently for each loop $\ell$ of $\omega$. Then, for each face *u* of ${\mathscr {D}}$, set 
  1.4 $$\begin{aligned} \Phi (u) = \ell _\circlearrowright (\overrightarrow{\omega }; u) - \ell _\circlearrowleft (\overrightarrow{\omega }; u), \end{aligned}$$
  where $\ell _\circlearrowright (\overrightarrow{\omega }; u)$ and $\ell _\circlearrowleft (\overrightarrow{\omega }; u)$ stand for the number of clockwise (resp. counter-clockwise) oriented loops of $\overrightarrow{\omega }$ surrounding *u*.

#### Proof

The correspondence between oriented loop configurations and Lipschitz functions defined by (1.4) is in fact a bijection. Indeed, the reverse mapping can be defined as follows: given a Lipschitz function $\varphi : {\mathscr {D}}\rightarrow {\mathbb {Z}}$, each loop of the corresponding (unoriented) loop configuration $\omega _\varphi$ is oriented clockwise if the values of $\varphi$ inside of the loop are higher than those outside, and is oriented counter-clockwise otherwise.

The push-forward of $\pi _{\mathscr {D}}$ under this bijection is a uniform measure on all oriented loop configurations on ${\mathscr {D}}$. Considering the projection on the set of unoriented loop configurations we obtain ${\mathbb {P}}_{\mathscr {D}}$, since each loop has two possible orientations. This proves (*i*), and (*ii*) follows readily. $\quad \square$

Using the correspondence between Lipschitz functions and loop configurations described above, Theorem 1.1 follows easily from Theorem 1.2.

#### Proof of Theorem 1.1

**(assuming Theorem** 1.2) (***i***) By Proposition 1.4*(ii)*, a random Lipschitz function $\Phi _{\mathscr {D}}$ distributed according to $\pi _{\mathscr {D}}$ can be sampled from a random loop configuration $\omega$ distributed according to ${\mathbb {P}}_{\mathscr {D}}$ by orienting each loop of $\omega$ uniformly and independently. Then $\Phi _{\mathscr {D}}(0)$ has the distribution of a simple random walk on ${\mathbb {Z}}$ with $N_{\mathscr {D}}$ steps, where $N_{\mathscr {D}}$ is the number of loops in $\omega$ surrounding 0. Thus, $\text {Var}(\Phi _{\mathscr {D}}(0)) = {\mathbb {E}}_{\mathscr {D}}(N_{\mathscr {D}})$. The conclusion follows from (1.3).

***(ii)*** Using the coupling from Proposition 1.4, we get that for any $u\in F({\mathscr {D}})$, the value of $\Phi _{\mathscr {D}}(u) - \Phi _{\mathscr {D}}(0)$ is a function of number of loops separating *u* from 0 and their orientations. By items (*i*) and (*ii*) of Theorem 1.2 the infinite-volume limit of ${\mathbb {P}}_{\mathscr {D}}$ exists and consists only of finite loops. Thus, the infinite-volume limit of $\Phi _{\mathscr {D}}- \Phi _{\mathscr {D}}(0)$ also exists.

***(iii)*** We will prove the statement for $\Phi _{\mathbb {H}}$; that for $\Phi _{\mathscr {D}}$ is proved in the same way. Similarly to the previous items, we have

1.5 $$\begin{aligned} \mathrm {Var}(\Phi _{\mathbb {H}}(x) - \Phi _{\mathbb {H}}(y)) = {\mathbb {E}}_{\mathbb {H}}(N_{x \setminus y} + N_{y \setminus x}), \end{aligned}$$

where $N_{x\setminus y}$ stands for the number of loops surrounding *x* but not *y* and $N_{y \setminus x}$ for those surrounding *y* but not *x*.

For the lower bound, notice that $N_{x\setminus y}$ is larger than the number of loops surrounding *x* and contained in $\Lambda _{|x-y|}$. Thus, by (1.3), ${\mathbb {E}}_{\mathbb {H}}(N_{x \setminus y}) \ge c \log |x-y|$ for some universal constant $c > 0$. The desired lower bound on $\mathrm {Var}(\Phi _{\mathbb {H}}(x) - \Phi _{\mathbb {H}}(y))$ follows.

For the upper bound, define $\Gamma$ to be the outermost loop surrounding *x* but not *y*, provided such a loop exists. Let $\gamma$ be a possible realisation of $\Gamma$ and let $\mathrm {Int}(\gamma )$ be the interior of the domain delimited by $\gamma$. Notice that the event $\{\Gamma = \gamma \}$ is measurable in terms of the configuration on and outside $\gamma$. Therefore, conditionally on $\Gamma = \gamma$, the restriction of ${\mathbb {P}}_{\mathbb {H}}$ to $\mathrm {Int}(\gamma )$ is the uniform measure among all loop configuration in $\mathrm {Int}(\gamma )$, which is to say it is equal to ${\mathbb {P}}_{\mathrm {Int}(\gamma )}$. Thus

$$\begin{aligned} {\mathbb {E}}_{\mathbb {H}}(N_{x\setminus y}) = {\mathbb {P}}( N_{x\setminus y} > 0) + \sum _\gamma {\mathbb {E}}_{\mathrm {Int}(\gamma )}(N_{\mathrm {Int}(\gamma )}(x))\cdot {\mathbb {P}}_{\mathbb {H}}(\Gamma = \gamma ), \end{aligned}$$

where the sum is over all possible realisations $\gamma$ of $\Gamma$ and $N_{\mathrm {Int}(\gamma )}(x)$ in the right hand side stands for the number of loops surrounding *x* and contained in $\mathrm {Int}(\gamma )$. Now, since $y \notin \mathrm {Int}(\Gamma )$, $\mathrm {dist}(x,\mathrm {Int}(\gamma )^c) \le |x-y|$ for any path $\gamma$ appearing in the sum. Thus, (1.3) proves that ${\mathbb {E}}_{\mathbb {H}}(N_{\mathrm {Int}(\gamma )}(x)) \le 1 + C \log |x-y|$ for some universal constant *C*.

The same holds for ${\mathbb {E}}_{\mathbb {H}}(N_{y\setminus x})$. Using this and (1.5), we obtain the desired upper bound on $\mathrm {Var}(\Phi _{\mathbb {H}}(x) - \Phi _{\mathbb {H}}(y))$. $\quad \square$

Let us briefly comment on the uniqueness of infinite-volume measures for Lipschitz functions. One may think that, due to Theorem 1.3, $\pi _{\mathbb {H}}$ should be the only infinite-volume measure with the property that its restriction to any finite domain is uniform among Lipschitz functions that take the value 0 at the origin. This is not the case. Indeed, the correspondence between the loop and Lipschitz functions models is not perfect, and does not allow us to deduce this.

Moreover the claim is false, as an infinite family of infinite-volume measures for uniform Lipschitz functions is expected to exist, one for each global “slope”. The loop representation of any of these contains infinite paths and is not Gibbs in the sense of (DLR).

**Structure of the paper** The rest of the paper is entirely dedicated to the loop *O*(2) model with $x = 1$. In Section 2 we derive a representation of the loop model in terms of two loop *O*(1) configurations conditioned not to intersect. These are in turn represented in terms of spin configurations that are shown to satisfy the FKG inequality and a certain form of Spatial Markov property.

In Section 3 this spin representation is used to construct an infinite-volume, ergodic loop *O*(2) measure. The infinite-volume measure is then shown to be unique (in some sense that will be made precise later). In doing so, we show that 0 is surrounded by infinitely many loops. For height functions, this corresponds to the delocalisation of $\Phi (0)$ or equivalently to the divergence of covariances. At this stage, the delocalisation/divergence is not quantitative.

Section 4 contains a dichotomy theorem. In the language of uniform Lipschitz functions, the dichotomy theorem roughly states that the covariance between two points either is bounded or diverges logarithmically in the distance between the points.

Finally, in Section 5, the non-quantitative delocalisation result and the dichotomy theorem are used to prove Theorem 1.2. Theorem 1.3 is also proved here. Moreover, we provide an RSW result for height functions in Section 5.4.

The paper is structured so as to isolate the different ingredients of our argument; some of them may be useful for the analysis of the loop *O*(*n*) model with other values of *n* and *x*, or other similar models. We further discuss in Section 2.1 the various properties of the loop *O*(2) model that are necessary for our proof.

**Notation** Below is a list of notation used throughout the paper. Some of it was already mentioned, some is new.

Recall that ${\mathbb {H}}$ denotes the hexagonal lattice; its dual is the triangular lattice, written ${\mathbb {H}}^* = {\mathbb {T}}$. We will call edge-path any finite or infinite sequence of adjacent edges of ${\mathbb {H}}$ with no repetitions. A face-path is a sequence of adjacent faces of ${\mathbb {H}}$ with no repetitions, or equivalently it is a path on ${\mathbb {T}}$ that does not visit the same vertex twice.

Domains ${\mathscr {D}}= (V({\mathscr {D}}),E({\mathscr {D}}))$ are interior of edge-polygons of ${\mathbb {H}}$. The edges of the polygon form the edge-boundary of ${\mathscr {D}}$, written $\partial _E{\mathscr {D}}$. The faces of ${\mathbb {H}}$ adjacent to $\partial _E{\mathscr {D}}$ and inside $\partial _E{\mathscr {D}}$ (outside, respectively) form the inner face boundary of ${\mathscr {D}}$, written $\partial _\mathrm {in}{\mathscr {D}}$ (and the outer face-boundary written $\partial _\mathrm {out}{\mathscr {D}}$, respectively). The set of faces of ${\mathbb {H}}$ inside $\partial _E{\mathscr {D}}$ is written $F({\mathscr {D}})$; those not adjacent to $\partial _E{\mathscr {D}}$ form the interior of ${\mathscr {D}}$, denoted by $\mathrm {Int}({\mathscr {D}}) = F({\mathscr {D}}) \setminus \partial _\mathrm {in}{\mathscr {D}}$. The dual ${\mathscr {D}}^* = (V({\mathscr {D}}^*), E({\mathscr {D}}^*))$ of ${\mathscr {D}}$ is the induced subgraph of ${\mathbb {T}}$ with vertex set $F({\mathscr {D}})$.

An edge configuration on ${\mathscr {D}}$ is an element $\omega \in \{0,1\}^{E({\mathscr {D}})}$; it is identified to the graph with vertex set $V({\mathscr {D}})$ and edge-set $\{e \in E({\mathscr {D}}) :\, \omega (e) = 1\}$. Write  to indicate that two vertices *u*, *v* of $V({\mathscr {D}})$ are connected in $\omega$. The same notation applies to ${\mathbb {H}}$ and ${\mathscr {D}}^*$.

A spin configuration on ${\mathscr {D}}$ is an element $\sigma \in \{-,+\}^{F({\mathscr {D}})}$; the notation extends to ${\mathbb {H}}$. Below we will use two superposing spin configurations. We identify one as red, the other as blue and denote the relevant spins by  and  for legibility.

For a red-spin configuration  and two faces $u,v \in F({\mathscr {D}})$, write  (or  when the choice of ${\mathscr {D}}$ is unclear) to indicate that there exists a face-path in ${\mathscr {D}}$ starting at *u* and ending at *v*, formed entirely of faces with $\sigma$-spin . Such a path will be called a -path or simple- path. Connected components for this notion of connectivity are called -clusters.

A double- path will be an edge-path for which all adjacent faces have spin ; connections by double- paths will be denoted by . The same applies to spins  and .

Write  for the negation of $\leftrightarrow$.

## 1+1 = 2

Fix a domain ${\mathscr {D}}$. Choose a loop configuration $\omega$ according to ${\mathbb {P}}_{{\mathscr {D}}}$ and colour each loop of $\omega$ in either red or blue, with equal probability, independently for each loop. Extend ${\mathbb {P}}_{\mathscr {D}}$ to include this additional randomness. Write $\omega _r$ and $\omega _b$ for the configurations of blue and red loops. Then, for any two disjoint loop configurations $\omega _r,\omega _b$,

$$\begin{aligned} {\mathbb {P}}_{\mathscr {D}}(\omega _r,\omega _b)&=\frac{1}{Z({\mathscr {D}})}2^{\ell (\omega )} \big (\tfrac{1}{2}\big )^{\ell (\omega _r)} \big (\tfrac{1}{2}\big )^{\ell (\omega _b)}= \frac{1}{Z({\mathscr {D}})}. \end{aligned}$$

In other words, ${\mathbb {P}}_{\mathscr {D}}$ is the uniform distribution on pairs of loop configurations $(\omega _r,\omega _b)$ that do not to intersect.

In the context of Lipschitz functions, one may think of $\omega _r$ as the level lines with higher value on the inside and $\omega _b$ as those with higher value on the outside (that is the clockwise and counter-clockwise, respectively, oriented loops in the language of (1.4)). While accurate, this interpretation is not relevant below.

Keeping the idea of colouring loops as the intuition, in the next section we introduce a measure on pairs of red and blue $\pm 1$ spin configurations. Though this measure is tightly linked to the loop *O*(2) measure on pairs of red and blue loops and under certain boundary conditions these two measures will be shown to coincide, we emphasise that this is not always the case.

To shorten notation, we will use the symbols  to denote the values of red spins and  for blue spins.

### Spin representation

Define $\mu _{\mathscr {D}}$ to be the uniform measure on all pairs of spin configurations  and  such that for every two adjacent faces $u,v\in F({\mathscr {D}})$ at least one of the equalities $\sigma _r(u)=\sigma _r(v)$ and $\sigma _b(u)=\sigma _b(v)$ holds. We call such configurations $\sigma _r$, $\sigma _b$ *coherent* and denote this relation by $\sigma _r\perp \sigma _b$.

Given a spin configuration $\sigma \in \{\pm 1\}^{\mathscr {D}}$, define $\omega (\sigma )$ to be set of edges of ${\mathscr {D}}$ separating adjacent faces bearing different spin in $\sigma$. Then $\omega (\sigma )$ consists of disjoint loops and paths linking boundary vertices in ${\mathscr {D}}$.

The correspondence $\sigma \mapsto \omega (\sigma )$ is a classical tool in the study of the Ising model, called the high temperature representation (see for instance [19, Sec. 3.10.1]). If $\sigma$ is chosen according to a Ising distribution, then $\omega (\sigma )$ has the law of a loop *O*(1) model, with parameter *x* depending on the temperature of the Ising measure. For the loop *O*(*n*) model with general values of *n*, this correspondence was used in [13] with the name cluster representation.

The following proposition describes the relation between $\mu _{\mathscr {D}}$ and ${\mathbb {P}}_{{\mathscr {D}}}$. Define 2.1where $\equiv$ should be understood as “equal everywhere to”. The notation  comes from Theorem 2.3, where these boundary conditions are shown to be equivalent to setting  on the interior boundary of ${\mathscr {D}}$ and  on its exterior boundary.

#### Proposition 2.1

If the couple $(\sigma _r,\sigma _b)$ has law , then the couple $(\omega (\sigma _r), \omega (\sigma _b))$ has law ${\mathbb {P}}_{\mathscr {D}}$. In particular $\omega (\sigma _r)\cup \omega (\sigma _b)$ has the law of the loop *O*(2) model on ${\mathscr {D}}$.

#### Proof

The map $\sigma _r\mapsto \omega (\sigma _r)$ is a bijection between spin configurations   that are equal to  on $\partial _\mathrm {in}{\mathscr {D}}$ and all loop configurations on ${\mathscr {D}}$. Indeed, due to the constant spin of $\sigma _r$ on $\partial _\mathrm {in}{\mathscr {D}}$, $\omega (\sigma _r)$ is indeed a loop configuration. Moreover, the reverse mapping is the following: a loop configuration $\omega$ on ${\mathscr {D}}$ is mapped to the spin configuration  that is equal to  (resp. ) at all faces of ${\mathscr {D}}$ that are surrounded by an even (resp. odd) number of loops of $\omega$.

Similarly, the map $\sigma _b \mapsto \omega (\sigma _b)$ defined on the set of spin configurations  that are constant on $\partial _\mathrm {in}{\mathscr {D}}$ and taking values in ${\mathscr {L}}({\mathscr {D}})$ is two to one, due to its invariance under global spin flip.

The condition $\sigma _r\perp \sigma _b$ corresponds to $\omega (\sigma _r)\cap \omega (\sigma _b) = \emptyset$. Thus,  induces a uniform measure on all pairs $(\omega (\sigma _r), \omega (\sigma _b))$ of non-intersecting red and blue loop configurations on ${\mathscr {D}}$, that is ${\mathbb {P}}_{\mathscr {D}}$. As described above, the marginal of this measure on the non-coloured loop configuration $\omega (\sigma _r)\cup \omega (\sigma _b)$ is the loop *O*(2) measure on ${\mathscr {D}}$. $\quad \square$

#### Remark 2.2

Extensions of the statement to all $x \ne 1$ are possible and result in non-uniform measures on pairs of spin configurations. As already mentioned, the correspondence between double-spins and loops does not extend to general boundary conditions for the loop *O*(2) model.

We will show below that, under $\mu _{\mathscr {D}}$, the marginals $\sigma _r$ and $\sigma _b$ satisfy the FKG inequality. Moreover the spin measures of the type $\mu _{\mathscr {D}}$ satisfy the Spatial Markov property in the following sense. If ${\mathscr {D}}'$ is a domain contained in some larger domain ${\mathscr {D}}$, then the restriction of $\mu _{\mathscr {D}}$ to ${\mathscr {D}}'$, conditionally on the spins $\sigma _r, \sigma _b$ outside ${\mathscr {D}}'$, is entirely determined by the values of $\sigma _r$ and $\sigma _b$ on $\partial _\mathrm {out}{\mathscr {D}}$.

It may be tempting to think that these two observations suffice to apply the techniques developed for the random-cluster model to our setting (such as those of [17, 18]). Unfortunately this is easier said than done. Indeed, many of these techniques use a form of monotonicity of boundary conditions. In our case, it is unclear how to compare boundary conditions consisting of pairs of spins, as the FKG inequality applies only individually to the single-spin marginals of $\mu _{\mathscr {D}}$.

To circumvent this difficulty, we will focus our study on one of the single-spin marginals of $\mu _{\mathscr {D}}$; we arbitrarily choose the red-spin marginal, and call it $\nu _{\mathscr {D}}$. As already stated, this measure satisfies the FKG inequality, but fails to have a general spatial Markov property. However, we show in Theorem 2.3 and Corollary 2.4 that a limited version of the spatial Markov property applies to $\nu _{\mathscr {D}}$, under certain restrictions.

One may attempt to apply the same strategy to other values of *n* and *x*. Our argument is quite intricate, and different parts of it use different properties of the double spin representation described above. The paper is organised to separate the different arguments, so as to facilitate the identification of blocks that may be applied to other models. Below is brief list of the essential properties of the double spin representation and their uses.

- The FKG inequality for the red-spin marginal is crucial and is used extensively throughout the proof. As mentioned in Remark 2.11 *(iii)*, the FKG inequality extends to the red-spin marginal of a certain double spin representation of the loop *O*(*n*) model with parameters $n \ge 2$ and $x \le \frac{1}{\sqrt{n-1}}$.
- That $x = 1$ is essentially only used for the spatial Markov property. In its current form, the property does not apply to $x \ne 1$.
- The symmetry between the red and blue spin marginals (which, in light of Remark 2.11 *(iii)* boils down to $n =2$) is akin to a self-duality property, and is used to prove RSW type estimates (see Lemma 3.8).

Finally, let us mention that it is expected that the loop *O*(2) model for $$n =2$$ and $$x \ge 1/\sqrt{2}$$ has a similar behaviour to the case $$x = 1$$, that is macroscopic loops exist at every scale. However, for all $$ n>2$$ and any $$x > 0$$ or $$n =2$$ and $$x < 1/\sqrt{2}$$, loops are expected to exhibit exponential decay. Thus, parts of our proof need to fail for more general values of *n* and *x*. The dichotomy theorem of Section 4 (or similar statements) may be expected to hold for all values of *n* and *x*, but no proof is generally available.

### Spatial Markov property

In general, the measures $\nu _{\mathscr {D}}$, that is the red-spin marginals of $\mu _{\mathscr {D}}$, do not have the spatial Markov property. However, a version of this property holds in certain cases. Recall the definition (2.1) of  and set Let  and , respectively, be the marginals on $\sigma _r$ of the above two measures. Define the measures , ,  etc. in a similar ways, and write  etc. their red-spin marginals.

#### Theorem 2.3

(Spatial Markov property). Let ${\mathscr {D}},{\mathscr {D}}'\subset {\mathbb {H}}$ be two domains such that $\partial _E{\mathscr {D}}\subset E({\mathscr {D}}')$. Let  and  be two coherent spin configurations on ${\mathscr {D}}'$.

(i) if  on $\partial _\mathrm {in}{\mathscr {D}}\cup \partial _\mathrm {out}{\mathscr {D}}$, then 2.2
(ii) if  on $\partial _\mathrm {in}{\mathscr {D}}$,  on $\partial _\mathrm {out}{\mathscr {D}}$ and $s:=\tau _b(u)$ for some $u\in \partial _\mathrm {out}{\mathscr {D}}$ , then 2.3

where symbol  means that the two measures are equal when $\sigma _r$ and $\sigma _b$ are restricted to ${\mathscr {D}}$.

#### Proof

All measures under consideration are uniform over sets of coherent pairs $(\sigma _r,\sigma _b)$ that agree with the corresponding boundary conditions. Thus, it is enough to show that the two sets corresponding to the two sides of (2.2), and of (2.3), respectively, are equal.

*(i)*: Consider a pair of coherent configurations  and  contributing to the RHS of (2.2); let us show that they also contribute to the LHS. By definition,  on $\partial _\mathrm {in}{\mathscr {D}}$, which is to say that $\sigma _r = \tau _r$ on $\partial _\mathrm {in}{\mathscr {D}}$. It remains to check that, if $\sigma _r$ and $\sigma _b$ are completed by $\tau _r$ and $\tau _b$, respectively, on $F({\mathscr {D}}')\setminus F({\mathscr {D}})$, they are coherent on ${\mathscr {D}}'$. For edges of $E({\mathscr {D}})$ and $E({\mathscr {D}}') \setminus (E({\mathscr {D}}) \cup \partial _E{\mathscr {D}})$, the coherence condition follow from the coherence of $\sigma _r$ with $\sigma _b$ and that of $\tau _r$ with $\tau _b$, respectively. For edges of $\partial _E{\mathscr {D}}$ the statement holds because both faces adjacent to each such edge are  in $\sigma _r$.
  The reverse direction is straighforward since each pair of configurations  and  contributing to the LHS of (2.2) is coherent and satisfies  on $\partial _\mathrm {in}{\mathscr {D}}$.
*(ii)*: The values of $\tau _r$ imply that $\tau _b$ is constant on $\partial _\mathrm {in}{\mathscr {D}}\cup \partial _\mathrm {out}{\mathscr {D}}$. Similarly, the definition of  requires that $\sigma _b$ be constant on $\partial _\mathrm {in}{\mathscr {D}}$ in the RHS of (2.3). The values of $\tau _b$ and $\sigma _b$ on $\partial _\mathrm {in}{\mathscr {D}}$ are the same because of the condition $\tau _b(u)= \sigma _b(v)=s$ for some $u\in \partial _\mathrm {in}{\mathscr {D}}$ and $v\in \partial _\mathrm {out}{\mathscr {D}}$. Thus, the pairs $(\tau _r,\tau _b)$ and $(\sigma _r,\sigma _b)$ agree on $\partial _\mathrm {in}{\mathscr {D}}$ and as a consequence these boundary values impose the same distribution on the LHS and the RHS of (2.3).$\quad \square$

Summing equalities of Theorem 2.3 over all possibilities for $\sigma _b$, we get the following corollary for the red-spin marginals of the measures.

#### Corollary 2.4

(Spatial Markov property for $\nu$). Let ${\mathscr {D}}, {\mathscr {D}}'$ be two domains such that $\partial _E{\mathscr {D}}\subset E({\mathscr {D}}')$. Let . Then the following statements hold:

(i) if  on $\partial _\mathrm {in}{\mathscr {D}}\cup \partial _\mathrm {out}{\mathscr {D}}$, then
(ii) if  on $\partial _\mathrm {in}{\mathscr {D}}$ and  on $\partial _\mathrm {out}{\mathscr {D}}$, then

where by symbol  we mean that the two measures are equal when $\sigma _r$ is restricted to ${\mathscr {D}}$.

#### Remark 2.5

It is tempting to think that the above Spatial Markov property holds for any boundary conditions on $\partial _\mathrm {in}{\mathscr {D}}\cup \partial _\mathrm {out}{\mathscr {D}}$. This is not the case. One significant example is that of the boundary conditions consisting of four arc of alternating spins , , , . Indeed, these boundary conditions are coherent with non-intersecting loop configurations^1^$(\omega _r, \omega _b)$ where  $\omega _b$ contains

- paths between the arcs ,
- paths between the arcs  or
- none of the above.

The three cases above are mutually exclusive. Depending on the red configuration outside ${\mathscr {D}}$ one or both of the first two cases may be excluded.

### FKG inequality

In this section we show that the red-spin marginals $\nu$ of the measures $\mu$ satisfiy the FKG inequality. This property is crucial to all our proofs. Similar properties were found in [13] for the single-spin representation of the loop *O*(*n*) for a certain range of parameters and in [22] for a spin representation of height functions on ${\mathbb {Z}}^2$ arising from the six-vertex model.

Fix some domain ${\mathscr {D}}$. We start by introducing a partial order on . Given two elements  we say that $\sigma \le \tau$ if $\sigma (u)\le \tau (u)$ for every $u\in F({\mathscr {D}})$, where by convention . An event  is called increasing if for any $\sigma \in A$ and  such that $\sigma \le \tau$, we have $\tau \in A$.

A probability measure ${\mathbb {P}}$ on  is said to satisfy the FKG inequality (or called positively associated) if for any two increasing events , we have

2.4 $$\begin{aligned} {\mathbb {P}}(A\cap B) \ge {\mathbb {P}}(A)\cdot {\mathbb {P}}(B). \end{aligned}$$

Recall that the marginal of $\mu _{\mathscr {D}}$ on the red spin configurations is denoted by $\nu _{\mathscr {D}}$.

#### Theorem 2.6

The measure $\nu _{\mathscr {D}}$ satisfies the FKG inequality (2.4).

**Fig. 3:**
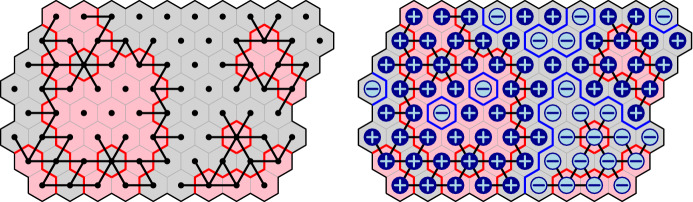
*Left:* A red-spin configuration on a domain ${\mathscr {D}}$ and the associated loops. The pink faces correspond to red spin , while the gray ones to red spin . The graph $\theta (\sigma _r)$ is drawn in black. *Right:* A blue-spin configuration coherent with the red one

Before proving the FKG inequality, let us compute $\nu _{\mathscr {D}}$. For a spin configuration $\sigma$ on ${\mathscr {D}}$, let $\theta (\sigma ) \in \{0,1\}^{E({\mathscr {D}}^*)}$ be the set of all edges $e=uv\in E({\mathscr {D}}^*)$ such that $\sigma (u) \ne \sigma (v)$. If $\sigma$ is associated to a loop configuration $\omega$, then $e^*\in \theta (\sigma )$ if and only if *e* is present in $\omega$. For readers familiar with the notion of duality in percolation (where the dual configuration is written $\omega ^*$), we mention that $\theta (\sigma ) = (\omega ^*)^c$. See Figure 3 for an example. Denote by $k(\theta (\sigma ))$ the number of connected components of $\theta (\sigma )$; note that isolated vertices of ${\mathscr {D}}^*$ (that is faces of ${\mathscr {D}}$) are also counted as connected components.

#### Proposition 2.7

 

(i) The law of $\sigma _r$ under $\mu _{\mathscr {D}}$ is given by
  2.5 $$\begin{aligned} \nu _{\mathscr {D}}(\sigma _r) =\frac{1}{Z_{\mathscr {D}}}\, 2^{k(\theta (\sigma _r))}, \end{aligned}$$
  where $Z_{\mathscr {D}}$ is a normalising constant.
(ii) The law of $\sigma _b$ on ${\mathscr {D}}$ under the conditional measure $\mu _{\mathscr {D}}(.\,|\, \sigma _r)$ is obtained by colouring independently and uniformly the clusters of $\theta (\sigma _r)$ in either  or .

#### Proof

Let  and consider any  that is coherent with $\sigma _r$. For any two faces $u,v\in F({\mathscr {D}})$ corresponding to vertices in $V({\mathscr {D}}^*)$ that are connected by an edge in $\theta (\sigma _r)$, we have $\sigma _b(u) = \sigma _b(v)$. Thus, $\sigma _b$ has a constant value on each connected component of $\theta (\sigma _r)$. Moreover, there is no restriction on values of $\sigma _b$ on different connected components of $\theta (\sigma _r)$. Thus, there are exactly $2^{k(\theta (\sigma _r))}$ blue spin configurations coherent with $\sigma _r$, and *(i)* follows readily. In addition, when conditioned on $\sigma _r$, the measure on these blue spin configurations is uniform, thus asserting *(ii)*. $\quad \square$

#### Remark 2.8

A straightforward adaptation of the proof above shows that, for ${\mathscr {A}}\subset F({\mathscr {D}})$, the law of $\sigma _r$ under  is given by $\frac{1}{Z}\, 2^{k_{\mathscr {A}}(\theta (\sigma _r))}$, where $k_{\mathscr {A}}(\theta (\sigma _r))$ is the number of connected components of $\theta (\sigma _r)$ when all components intersecting ${\mathscr {A}}$ are counted as a single one. When ${\mathscr {A}}$ is connected, $k_{\mathscr {A}}(\theta (\sigma _r))$ may be viewed as the number of connected components of the configuration obtained by adding to $\theta (\sigma _r)$ all edges between pairs of adjacent faces of ${\mathscr {A}}$.

As a consequencewhere $k_{\partial {\mathscr {D}}}(\theta (\sigma _r))$ is the number of connected components of $\theta (\sigma _r)$, where all components intersecting $\partial _\mathrm {in}{\mathscr {D}}$ are counted as a single one.

We are in a position to prove Theorem 2.6.

#### Proof of Theorem 2.6

By [25, Thm. 4.11], it is enough to show the FKG lattice condition, which states that, for any two spin configurations $\sigma$ and ${{\tilde{\sigma }}}$,

2.6 $$\begin{aligned} \nu _{\mathscr {D}}(\sigma \vee {{\tilde{\sigma }}}) \nu _{\mathscr {D}}(\sigma \wedge {{\tilde{\sigma }}}) \ge \nu _{\mathscr {D}}(\sigma ) \nu _{\mathscr {D}}({{\tilde{\sigma }}}), \end{aligned}$$

where  are defined by $\sigma \vee {{\tilde{\sigma }}}(u) = \max (\sigma (u),{{\tilde{\sigma }}}(u))$ and $\sigma \wedge {{\tilde{\sigma }}}(u) = \min (\sigma (u),{{\tilde{\sigma }}}(u))$ for every $u\in {\mathbb {T}}$. Moreover, by [24, Thm. (2.22)], it is enough to show (2.6) for any two configurations which differ for exactly two faces. That is, that for any  and $u, v\in F({\mathscr {D}})$ two distinct faces,2.7where $\sigma ^{ab}$ is the configuration coinciding with $\sigma$ except (possibly) at *u* and *v*, and such that $\sigma ^{ab}(u)= a$ and $\sigma ^{ab}(v)= b$. By Proposition 2.7, the ratio of the LHS and RHS of (2.7) is written2.8Our goal is thus to show that2.9First we will treat the simple case where $\sigma$ is such that  in  and  in . Then *u* and *v* are not adjacent and there exist two paths or circuits, one of  the other of , that separate *u* from *v* in ${\mathscr {D}}$. Hence, there exists a path or loop $\gamma$ in $\omega (\sigma )$ that separates *u* from *v* and does not contain any edges of the faces *u* or *v*. For any choice of , edges in ${\mathbb {T}}$ that cross $\gamma$ belong to $\theta (\sigma ^{ab})$, thus forming a path or a circuit of edges in $\theta (\sigma ^{ab})$ that separates *u* from *v*. The effect on $k(\theta (\sigma ))$ of switching the spin at *v* from  to  is then independent of the value of the spin at *u*:As a consequence, the LHS of (2.9) is zero.

We move on to the case where *u* and *v* are connected by a path of  or by a path of . Before diving into the core of the proof, we need to eliminate a degenerate case: when *u* and *v* are neighbouring faces and no face of ${\mathscr {D}}$ is adjacent to both *u* and *v*. Then ${\mathscr {D}}$ may be split into two domains ${\mathscr {D}}_u$ and ${\mathscr {D}}_v$ containing all faces connected to *u* in ${\mathscr {D}}\setminus \{v\}$ and those connected to *v* in ${\mathscr {D}}\setminus \{u\}$, respectively. It is then immediate to see that the number of connected components of  intersecting ${\mathscr {D}}_u$ is the same as that for . The same statement applies to  and . A similar statement may be formulated for ${\mathscr {D}}_v$, by pairing  with  and  with . Finally, in  and , faces *u* and *v* are in the same connected component, while in  and  they are in different components. Thus, we findHenceforth we may assume that, if *u* and *v* are neighbours, then there exists at least one face of ${\mathscr {D}}$ adjacent to both *u* and *v*. Moreover, we will suppose that *u* and *v* are connected by a path of  in  or by a path of  in . By symmetry, we may limit our study to the case where *u* is connected to *v* in  by a -path; when *u* and *v* are neighbours, we may choose the path to contains at least one vertex other than *u* and *v*.

Denote by *P* the -cluster of *u* (and implicitly of *v* as well) in ; denote by *M* the union of all -clusters in  that are adjacent to *u* or *v*. Both *P* and *M* are fixed sets of faces of ${\mathscr {D}}$. Then all the connected components of , , , and  that do not intersect $P\cup M$ are the same in these four configurations, and thus cancel out in (2.9). It remains to study the contribution of connected components of $\theta (.)$ that do intersect $P\cup M$.

For a spanning subgraph $\Theta$ of ${\mathscr {D}}^*$, define $k_P(\Theta )$ to be the number of connected components of $\Theta$ that intersect *P*, and  $k_M(\Theta )$ as number of connected components that intersect *M* and do not intersect *P*. Clearly, $k_P(\Theta )+ k_M(\Theta )$ is equal to the number of connected components in $\Theta$ that intersect $P\cup M$. Thus, is suffices to prove the following two inequalities:2.102.11We start by proving the easier inequality (2.11). Four types of components contribute to : those who contain faces adjacent to both *u* and *v*, those who contain faces adjacent to *u* but not *v*, those who contain faces adjacent to *v* but not *u*, and those containing no faces adjacent to *u* or *v*. Write $K_{\{u,v\}}$, $K_{\{u\}}$, $K_{\{v\}}$ and $K_{\emptyset }$ for the number of components in each category above. By the definition of $k_M$ and the fact that $u,v \in P$, any connected component contributing to  is such that all its faces that are adjacent to *u* or *v* have spin  in . When turning the spin of *u* from  to , all components of the type $K_{\{u,v\}}$, $K_{\{u\}}$ become connected to *u*, and thus cease to contribute to $k_M$. The same holds for *v*, and we find:Using that , we find that the LHS of (2.11) is equal to $K_{\{u,v\}}$, hence is non-negative.

Let us now prove (2.10). Denote by $E_u,E_v\subset E({\mathscr {D}}^*)$ the sets of all edges linking *u* (resp. *v*) to adjacent vertices in $V({\mathscr {D}}^*)$. The next claim constitutes the core of the proof and, as we will see below, implies readily (2.10). $\quad \square$

#### Claim 2.9

The following equalities hold:2.122.132.14

#### Proof

**Fig. 4:**
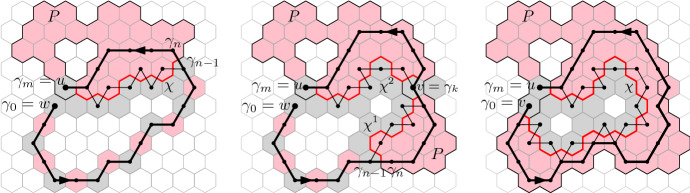
The constructions used in the proofs of (2.12) (left) and (2.14) (centre and right). Spins  are pink and  are gray; only spins of interest are depicted. The path $\gamma$ (black bold) uses faces of alternating spins until it enters *P*, then it continues on *P*, whose faces (except for *v* in the central and right diagrams) are of spin . The paths $\chi$, $\chi ^1$ and $\chi ^2$ (in red) are part of the boundary of *P* and separate faces of distinct spins. Their dual edges contain paths linking *u* to $\gamma _n$, *u* to *v* and *v* to $\gamma _n$, respectively

We start by showing (2.12). Note thatThus, it remains to show that for any face $w\sim u$,2.15Figure 4, left diagram, helps illustrate the construction below. Consider a face *w* neighbouring *u*, such that  and . Let $\gamma = (\gamma _0,\dots , \gamma _n)$ be a simple path of  with $\gamma _0 = w$, $\gamma _n \in P$ and such that $\gamma _0,\dots , \gamma _{n-1} \notin P$. By our assumption , we have $w \notin P$, so $n\ge 1$. Continue $\gamma$ by a face-path $\gamma _{n},\gamma _{n+1}, \dots , \gamma _{m}$ contained in *P* and with $\gamma _m =u$. (Note that we do not require that the path $\gamma _n \dots , \gamma _m$ be contained in .) Then it is necessary that , hence $\gamma _{m-1} \in P$, which is to say $n < m$. Finally set $\gamma _{m+1} = w$.

Then $\gamma$ is a non-trivial simple cycle on ${\mathbb {T}}$. Since the domain ${\mathscr {D}}$ is simply connected, $\gamma$ delimits a simply connected domain which we denote by $D_\gamma$. The boundary of $P \setminus \{u\}$ intersects $\gamma$ at two places: the midpoint of the edge $\gamma _{m-1}\gamma _m$ and the midpoint of the edge $\gamma _{n-1}\gamma _n$. Thus the boundary of $P\setminus \{u\}$ contains a path $\chi$ that is contained in $D_\gamma$ and that connects these two midpoints of edges.

Finally notice that, for any two adjacent faces *a*, *b* with $a \in P \setminus \{u\}$ and $b \notin P\setminus \{u\}$, we have  and , hence . Applying this to faces on either side of $\chi$, we find that all edges of ${\mathbb {T}}$ crossing $\chi$ are contained in . In particular, we deduce that *u* is connected in  to $\gamma _n$, hence also to *w*. This completes the proof of (2.12). The same argument proves (2.13).

We turn to the proof of (2.14). We will prove this in two steps:2.16The second equality above is implied by (2.13). Indeed, we have proved that no edge of   may connect two distinct clusters contributing to . That is also true for clusters contributing to , since the latter configuration dominates the former.

The first equality of (2.16) is similar to (2.12), with the only difference that it applies to  rather than . This apparent detail complicates the proof slightly as *u* is not necessarily connected to all points of *P* by paths of  in . The middle and right diagram of Figure 4 helps illustrate the argument below.

As for (2.12), the proof goes through the equivalent of (2.15). Fix a face *w* neighbouring *u* with  and which belongs to a connected component of  that intersects *P*. Our goal is to prove that *w* is connected to *u* in 

In a first instance let us suppose that $w \ne v$. Then, as in the proof of (2.12), we may produce a path $w = \gamma _0,\dots , \gamma _n,\dots , \gamma _m = u$ such that $\gamma _0,\dots , \gamma _{n-1} \notin P$, $\gamma _n,\dots , \gamma _m \in P$ and $\gamma _{0},\dots ,\gamma _{n}$ uses only edges of . If such a path may be constructed to not include *v*, then we choose $\gamma$ such, and the same reasoning as in (2.12) (applied with $P\setminus \{v\}$ instead of *P*) allows us to conclude that .

Suppose now that no path $\gamma$ with the properties above and which avoids *v* exists. Then pick $\gamma$ to visit *v* at some index $k \ge n$ and with $k < m -1$ (see Figure 4, center). We have $k \ge n$ since $v \in P$; we may pick $k < m-1$ since, even when *u* and *v* are adjacent, *u* is connected to *v* by a non-trivial path of , and we include this path in $\gamma$. It is also true that $k > n$, since  necessarily. Let $D_\gamma$ be the domain delimited by $\gamma$.

Consider the boundary of $P \setminus \{u,v\}$ inside the domain $D_\gamma$; it intersects the boundary of $D_\gamma$ at four points: the midpoint of the edges $\gamma _{n-1}\gamma _n$, $\gamma _{k-1}\gamma _k$, $\gamma _{k}\gamma _{k+1}$ and $\gamma _{m-1}\gamma _m$. Since no path $\gamma$ avoiding *v* exists, the boundary of $P \setminus \{u,v\}$ contains two non-empty segments $\chi ^1$ and $\chi ^2$ which connect $\gamma _{n-1}\gamma _n$ to  $\gamma _{k-1}\gamma _k$ and $\gamma _{k}\gamma _{k+1}$ to $\gamma _{m-1}\gamma _m$, respectively. By the choice of $\chi ^1$ and $\chi ^2$ as parts of the boundary of $P \setminus \{u,v\}$, all edges of ${\mathbb {T}}$ that intersect $\chi ^1$ and $\chi ^2$ are present in . In particular, we find  and , which implies that *u* is connected to *w* in .

Finally let us study the case when $w = v$ and hence *u* and *v* are adjacent (see Figure 4, right). Then, due to our assumption that *u* and *v* are connected by a non-trivial path of  in , we may choose a face-path $\gamma = \gamma _0,\dots ,\gamma _m$ with $m\ge 2$, $\gamma _0 = v$, $\gamma _m = u$ and  for all $1 \le k < m$. The cycle $\gamma \cup \{uv\}$ delimits a simply connected domain $D_\gamma$. By considering the interface between *P* and the  cluster of *u* in , we deduce the existence of an edge-path $\chi$ on $E(D_\gamma )$ with  on one side and  on the other, that starts on an edge adjacent to *v* and ends on one adjacent to *u*. This implies that , and the proof is complete. $\quad \square$

Using Claim 2.9, (2.10) becomes2.17The LHS above is the number of distinct connected components in  that contain at least one endpoint of an edge of $E_u$ minus one. The RHS is the same number for  instead of . Clearly, the former is greater or equal than the latter, and the proof of (2.10) is finished. $\quad \square$

Below we formulate several corollaries about the FKG inequality under various boundary conditions that we are going to use in the proofs.

#### Corollary 2.10

The FKG inequality (2.4) holds also in the following cases:

(i) for the red-spin marginal of $\mu _{\mathscr {D}}$, when the red spins are conditioned to take given values on a set of faces of ${\mathscr {D}}$ and the blue spins are conditioned to be  on a connected set of faces of ${\mathscr {D}}$. More precisely, for $\sigma _r$ chosen according to  , where ${\mathscr {A}}$ is any set of faces of ${\mathscr {D}}$, $\sigma _0$ is any red spin configuration on ${\mathscr {A}}$, and ${\mathscr {B}}$ is a connected set of faces of ${\mathscr {D}}$;
(ii) for the measures , ,  and .

#### Proof

*(i)* First let us show the FKG inequality when ${\mathscr {A}}$ is empty. As described in Remark 2.8, conditioning the blue spins to be  on ${\mathscr {B}}$ boils down to counting all connected components of $\theta (\sigma _r)$ that intersect ${\mathscr {B}}$ as a single one. When ${\mathscr {B}}$ is connected, this may be achieved by adding to $\theta (\sigma _r)$ all edges linking pairs of neighbouring vertices in ${\mathscr {B}}$. The proofs of (2.12), (2.13) and (2.14) adapt directly to this situation. Indeed, as already discussed in the proof above, adding edges to $\theta (\sigma _r)$ only helps in proving (2.12), (2.13) and (2.14). The rest of the proof of Theorem 2.6 applies directly.

Next assume that ${\mathscr {A}}$ is non-empty and $\sigma _0$ is given. The FKG lattice condition for  is a subset of the inequalities that constitute the FKG lattice condition for . Since the latter were proved to hold, so do the former.

*(ii)* Let ${\mathscr {D}}'$ be a domain containing $F({\mathscr {D}}) \cup \partial _\mathrm {out}{\mathscr {D}}$. Then $\nu _{{\mathscr {D}}'}$ satisfies the FKG inequality. By point *(i)* and the Spatial Markov property (Corollary 2.4), the FKG inequality also applies to , ,  and . $\quad \square$

#### Remark 2.11

 

(i) The FKG inequality does not apply to $\sigma _r$ under  when ${\mathscr {B}}$ is not connected. A counter-example is provided by a domain formed of six faces in a line, with ${\mathscr {B}}$ being formed of the first and last face, *u* and *v* being the second and fifth face, respectively, and $\sigma$ being the red spin configuration formed of alternating  and  spins.
  Nor does the FKG inequality apply to the red spin marginal of , where ${\mathscr {B}}_+$ and ${\mathscr {B}}_-$ are disjoint sets of faces of ${\mathscr {D}}$.
(ii) The proof of the FKG inequality only uses limited features of the hexagonal lattice. Indeed, it adapts to any planar trivalent graph whose set of faces forms a simply connected domain. It is however worth mentioning that the condition of simply connectedness is essential. Indeed, counter-examples may be given for sets of faces of ${\mathbb {H}}$ which are not simply connected: the counter-example of point (i) above may easily be adapted.
(iii) A similar instance of the FKG inequality extends to the loop *O*(*n*) model with $n\ge 2$ and $x\le 1/\sqrt{n-1}$, when the red spin configuration is obtained by colouring loops in red with probability 1/*n* and in blue otherwise, independently. The only difference in the proof is that the term $2^{k(\theta (\sigma ))}$ in (2.5) should be replaced by the partition function of the Ising model on the graph obtained by collapsing each cluster of $\theta (\sigma _r)$ into a single vertex. The FKG property of the FK-Ising representation then leads to the analogue of (2.17). We do not give further details of this generalisation as it is irrelevant here; the reader is referred to [22], where similar ideas are used to prove a FKG statement for the spin representation of a six-vertex model.

### Comparison between boundary conditions

Above he have introduced a number of boundary conditions for the positively associated measure $\nu _{\mathscr {D}}$. As for the random cluster model or other positively associated models, the boundary conditions may have an increasing or decreasing effect on the measure.

For two measures $\nu _1,\nu _2$ on  (where *F* is some non-empty set), say that $\nu _1$ stochastically dominates $\nu _2$, written $\nu _1 \ge _{\text {st}} \nu _2$, if for any increasing event , $\nu _1(A) \ge \nu _2(A)$.

#### Corollary 2.12

(Comparison between boundary conditions). 

(i) Let ${\mathscr {D}}$ be a domain and let ${\mathscr {A}}\subset F({\mathscr {D}})$. Let $\sigma ^1 \le \sigma ^2$ be two (red) spin configurations on ${\mathscr {A}}$. Then $\nu _{\mathscr {D}}( . \,|\, \sigma _r = \sigma ^1 \text { on }{\mathscr {A}})\le _{\text {st}} \nu _{\mathscr {D}}( . \,|\, \sigma _r = \sigma ^2 \text { on }{\mathscr {A}})$.
(ii) For any domain ${\mathscr {D}}$ the following comparison inequalities hold: 2.18

#### Proof

*(i)* Write ${\mathscr {A}}= {\mathscr {A}}_= \sqcup {\mathscr {A}}_\ne$, where ${\mathscr {A}}_=$ is the set of faces where $\sigma _1$ and $\sigma _2$ agree and ${\mathscr {A}}_{\ne }$ that where they disagree. By the ordering $\sigma _1 \le \sigma _2$, we deduce that $\sigma _1$ is constantly  on ${\mathscr {A}}_\ne$ while $\sigma _2$ is constantly  on this set. Due to the positive association of $\nu _{{\mathscr {D}}}( . \,|\, \sigma _r = \sigma _1 \text { on }{\mathscr {A}}_=)$ shown in Corollary 2.10,*(ii)* Let us begin with the first and last inequalities of (2.18). Let ${\mathscr {D}}'$ be a domain containing $F({\mathscr {D}}) \cup \partial _\mathrm {out}{\mathscr {D}}$. By point *(i)* above,The Spatial Markov property (Corollary 2.4) translates the above to . The first inequality of (2.18) is proved in the same way.

We move on to the middle inequality of (2.18). Considering (2.3) and the symmetry of blue spins, this inequality may be written aswhere the stochastic ordering refers only to the red-spin marginal. Clearly, the set $\partial _\mathrm {in}{\mathscr {D}}$ is connected in ${\mathbb {H}}^*$, thus the inequality follows from Corollary 2.10*(i)*. $\quad \square$

Let ${\mathscr {D}}$ be a domain with vertices *a*, *b*, *c*, *d* on its boundary $\partial _E {\mathscr {D}}$, arranged in counter-clockwise order, and such that the edges incident to *a*, *b*, *c* and *d* all belong to $\partial _E {\mathscr {D}}$ or to $E({\mathscr {D}})$. Call (*ab*) the segment of $\partial _E {\mathscr {D}}$ between *a* and *b*, when going around ${\mathscr {D}}$ in the counter-clockwise direction. Define (*bc*), (*cd*) and (*da*) similarly.

Let  be the uniform measure on pairs of coherent red and blue spin configurations on ${\mathscr {D}}$ with the property that $\sigma _r$ is equal to  on all faces of $\partial _\mathrm {in}{\mathscr {D}}$ adjacent to (*ab*) or (*cd*) and  on all other faces of $\partial _\mathrm {in}{\mathscr {D}}$. The condition above also imposes that the blue spins of the two faces of $\partial _\mathrm {in}{\mathscr {D}}$ that are adjacent to *a* are equal, and the same for the pairs of faces adjacent to *b*, *c* and *d*. Other than this, there is no restriction for the blue spins on $\partial _\mathrm {in}{\mathscr {D}}$. The marginal on red spins of the above is denoted by .

**Fig. 5:**
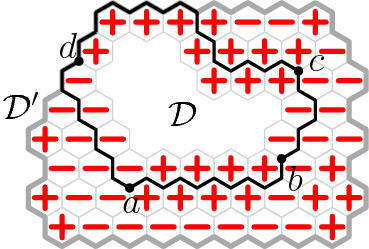
Two domains ${\mathscr {D}}\subset {\mathscr {D}}'$ bounded by the black and grey contours, respectively. The configuration in ${\mathscr {D}}' \setminus \mathrm {Int}({\mathscr {D}})$ imposes boundary conditions  on ${\mathscr {D}}$

Fix now a larger domain ${\mathscr {D}}'$ with ${\mathscr {D}}\subset {\mathscr {D}}'$ (possibly with $\partial _E{\mathscr {D}}\cap \partial _E{\mathscr {D}}' \ne \emptyset$). We say that a configuration $\tau _r$ on ${\mathscr {D}}' \setminus \mathrm {Int}({\mathscr {D}})$ imposes boundary conditions  on ${\mathscr {D}}$ if all the faces adjacent to $(ab) \cup (cd)$ but not to $(bc) \cup (da)$ have spin  in $\tau _r$ and all those adjacent to $(bc) \cup (da)$ but not to $(ab) \cup (cd)$ have spin . The faces of $\partial _\mathrm {in}{\mathscr {D}}\cup \partial _\mathrm {out}{\mathscr {D}}$ that are adjacent to both $(bc) \cup (da)$ and $(ab) \cup (cd)$ may have spins  or  in $\tau _r$^2^ (see Figure 5).

As already discussed in Remark 2.5, the spatial Markov property does not apply to the boundary conditions . Indeed, the connectivity of the edges of $\theta (\tau _r)$ that are adjacent to *a*, *b*, *c* and *d* influences the measure in ${\mathscr {D}}$ and is not determined by the spins on $\partial _\mathrm {in}{\mathscr {D}}\cup \partial _\mathrm {out}{\mathscr {D}}$. It may be that certain configuration $\sigma _b$ are awarded positive probability in , but null probability in $\mu _{{\mathscr {D}}'}(.\,|\, \sigma _r = \tau _r \text { on }{\mathscr {D}}' \setminus \mathrm {Int}({\mathscr {D}}))$. However, if we limit ourselves to the red-spin marginal, the Radon-Nikodim derivative of the second measure with respect to the first may be shown to be uniformly bounded.

#### Lemma 2.13

*(i)* Let ${\mathscr {D}}$ be a domain and *a*, *b*, *c*, *d* be points on $\partial _E{\mathscr {D}}$ as above. Let ${\mathscr {D}}'$ be a domain containing ${\mathscr {D}}$ and $\tau _r$ be a red spin configuration on ${\mathscr {D}}'\setminus \mathrm {Int}({\mathscr {D}})$, that imposes boundary conditions  on ${\mathscr {D}}$. Then, for any configuration ,*(ii)* Moreover, if $a,b,c,d \in \partial _E {\mathscr {D}}\cap \partial _E {\mathscr {D}}'$ and the arcs (*bc*) and (*da*) of  $\partial _E {\mathscr {D}}$ and $\partial _E {\mathscr {D}}'$ coincide, thenby which we mean that the former stochastically dominates the restriction to ${\mathscr {D}}$ of the latter.

*(iii)* Finally, if $a',b',c',d' \in \partial _E{\mathscr {D}}$ are another set of four points with the same properties as *a*, *b*, *c*, *d* and such that $(bc)\subset (b'c')$ and $(da)\subset (d'a')$, then

#### Proof

***(i)*** Both measures in the statement are supported on configuration  that agree with $\tau _r$ on $\partial _\mathrm {in}{\mathscr {D}}$. By Proposition 2.7, for any such configuration $\varsigma _r$,2.192.20where  are normalising constants and $k_{\mathscr {D}}(\theta (\varsigma _r \cup \tau _r))$ is the number of connected components of $\theta (\varsigma _r \cup \tau _r)$ that intersect ${\mathscr {D}}$. Indeed, the number of connected components that do not intersect ${\mathscr {D}}$ does not depend on $\varsigma _r$, hence cancel out.

Observe that $\theta (\varsigma _r \cup \tau _r)$ contains more connections than $\theta (\varsigma _r)$, hence fewer connected components. However, in $\theta (\varsigma _r)$, there are at most four distinct connected components that may be connected in  $\theta (\varsigma _r \cup \tau _r)$. Thus,

$$\begin{aligned} k(\theta (\varsigma _r)) - 3 \le k_{\mathscr {D}}(\theta (\varsigma _r \cup \tau _r)) \le k(\theta (\varsigma _r)). \end{aligned}$$

By summing the above over all configuration $\sigma _r$ in the support of  , we find . Inserting the last two inequalities in (2.19) provides the desired bound.

***(ii)*** We haveThe FKG inequality implies the desired stochastic domination.

***(iii)*** Let $A_+$ be the faces of $\partial _\mathrm {in}{\mathscr {D}}$ that are adjacent to $(a'b')$ or $(c'd')$ and $A_-$ be the faces of $\partial _\mathrm {in}{\mathscr {D}}$ that are adjacent to (*bc*) or (*da*). Set $A_\ne = \partial _\mathrm {in}{\mathscr {D}}\setminus (A_+\cup A_-)$. ThenCorollary 2.12*(i)* implies that the first measure dominates the second. $\quad \square$

## Infinite-Volume Measure: Existence and Uniqueness

In this section we construct an infinite-volume Gibbs measure for the loop *O*(2) model. As for the Ising, Potts or FK models, the infinite-volume limit will be created as a limit of finite-volume measures. The existence of the limit rests on the monotonicity of the measures $\nu _{\mathscr {D}}$ in their boundary conditions.

### Theorem 3.1

(Existence of limiting measure). For any increasing sequence of domains  $({\mathscr {D}}_n)_{n \ge 0}$ with $\bigcup _{n\ge 0} {\mathscr {D}}_n = {\mathbb {H}}$, the sequence of measures  converges to a measure  on . Moreover, the   is invariant under translations and rotations by multiples of $\pi /3$, ergodic with respect to translations, and has positively associated blue- and red-spin marginals.

The same argument may be used to construct measures ,  and  as limits of finite-volume measures with the proper boundary conditions. Below we prove that these measures are all equal to a single measure $\mu _{\mathbb {H}}$. We also show that the measures  converge to $\mu _{\mathbb {H}}$ as ${\mathscr {D}}$ increases to ${\mathbb {H}}$.

For the double-spin representation, the theorem below may be understood as a partial uniqueness theorem; see Remark 3.3 for more on why it is not a complete uniqueness theorem. For Lipschitz functions, the theorem below amounts to non-quantitative delocalisation: it proves that the value at 0 is not tight as the domain increases to ${\mathbb {H}}$, but does not offer the speed at which its variance increases.

For $n \ge 1$, let  be the event that there exists a simple closed path of edges of ${\mathbb {H}}$ surrounding $\Lambda _n$ with the property that the red spin of all faces adjacent to any of its edges is . The events ,  and  are defined similarly.

### Theorem 3.2

(Uniqueness of infinite-volume measure / Delocalisation). For any $n \ge 1$,3.1In particular , and we will simply write $\mu _{\mathbb {H}}$.

Also, for any sequence of finite domains ${\mathscr {D}}_n$ that increases to ${\mathbb {H}}$, the measures , ,  and  all converge to $\mu _{\mathbb {H}}$.

### Remark 3.3

The theorem above states that any finite-volume measure with any red boundary condition converges to $\mu _{\mathbb {H}}$. However, we do not claim this for mixed red and blue boundary conditions.

It may be tempting to believe that, for any assignment of red and blue spins $\xi _n^r,\xi _n^b$ on $\partial _\mathrm {in}{\mathscr {D}}_n$, the measure $\mu _{{\mathscr {D}}_n}$ conditioned to have spins $\xi _n^r$ and $\xi _n^b$ on $\partial _\mathrm {in}{\mathscr {D}}_n$ also converge to $\mu _{\mathbb {H}}$. Unfortunately this is not the case: counter examples may be created where the boundary conditions $\xi _n^r,\xi _n^b$ force one single configuration inside the domain.

The rest of the section is dedicated to the proofs of the two theorems above. The RSW theorem developed in Section 3.2 will be of great use also in Section 4.

### Infinite-volume measure for red marginal

We will work here only with the red-spin marginals $\nu$ of the measures $\mu$.

#### Theorem 3.4

(Limiting measure for red spins). For any increasing sequence of domains  $({\mathscr {D}}_n)_{n \ge 0}$ with $\bigcup _{n\ge 0} {\mathscr {D}}_n = {\mathbb {H}}$, the sequence of measures  converges to a measure  on . Moreover   is translation-invariant, ergodic with respect to translations and positively associated.

#### Proof

Let ${\mathscr {D}}$ and ${{\tilde{{\mathscr {D}}}}}$ be two finite domains, with ${\mathscr {D}}\subset {{\tilde{{\mathscr {D}}}}}$. Due to the FKG inequality (Theorem 2.6) and to the Spatial Markov property (Theorem 2.3) for the boundary conditions ,where the above only refers to the restrictions of the measures to ${\mathscr {D}}$. Thus, the sequence of measures  is decreasing, hence converges to a measure on , which we denote by .

Since the limit exists for any sequence of domains, it necessarily is the same for any sequence of domains $({\mathscr {D}}_n)_{n\ge 0}$. In particular, the same limit is obtained for any sequence $(\Lambda _n + z)_{n\ge 1}$ with $z \in V({\mathbb {H}})$, which implies that   is invariant under translations.

That  is positively associated for increasing events depending only on the state of finitely many faces follows by passing to the limit. The property extends to arbitrary increasing events by the monotone class theorem (see [24, Prop. 4.10]).

In order to prove that  is ergodic, we will show that it has the following mixing property. The measure  is said to be *mixing* if, for any events *A*, *B*, if $\tau _x(B)$ denotes the translation of *B* by some $x \in V({\mathbb {H}})$,3.2The above implies that  is ergodic with respect to translations, as explained in [24, Cor. 4.23]. By the monotone class theorem, it suffices to prove (3.2) for events *A* and *B* that are increasing and only depend on finitely many faces. We do this below.

Let *A*, *B* be increasing events depending only on the states of faces in some finite domain of ${\mathbb {H}}$. Fix $\epsilon > 0$. Then there exists $N \ge 1$ such that  and . Then, for any $x \in V({\mathbb {H}})$ with $|x| > 2N+2$, by positive association of  and (2.2),Conversely, positive association implies thatThese two inequalities and the fact that $\epsilon > 0$ is arbitrary imply (3.2), and hence the ergodicity of . $\quad \square$

### Crossing estimates for double-plus percolation (weak version)

We will work in the rest of the paper with two percolation models derived from spin configurations. Let us describe them for a red spin configuration $\sigma _r$ on ${\mathbb {H}}$; the definitions adapt readily to blue spins, to − instead of $+$, and to domains of ${\mathbb {H}}$.

The first corresponds to connections via face-paths of spins . This percolation, along with its paths, clusters etc. will be referred to as *simple-*; connections between two sets of faces *A* and *B* are denoted by . This notion was implicitly used in the proof of Theorem 2.6.

The second is termed *double-* percolation. The double-plus configuration $\mathrm {dp}(\sigma _r) \in \{0,1\}^{E({\mathbb {H}})}$ associated to $\sigma _r$ is formed of the edges of ${\mathbb {H}}$ whose two adjacent faces have spins . We regard $\mathrm {dp}(\sigma _r)$ as a bond percolation on ${\mathbb {H}}$ and use the ensuing notion of connectivity. In particular, for sets *A* and *B* of vertices, we write  for the event that there exists an edge-path in $\mathrm {dp}(\sigma _r)$ with one endpoint in *A* and the other in *B*. More generally, we call a double- path, or a double-path of spin , a path of edges in $\mathrm {dp}(\sigma _r)$. All notions related to this percolation (clusters, crossings, circuits etc.) will be referred to as double-. Thus, the event  of Theorem 3.2 may be described as the existence of a double- circuit surrounding $\Lambda _n$. The appeal of this second, more restrictive percolation model is that double- circuits isolate the inside from the outside in the sense of the Spatial Markov property (2.2).

Since $\sigma _r$ is positively correlated under $\mu _{\mathscr {D}}$, so is $\mathrm {dp}(\sigma _r)$. Indeed any event *A* which is increasing for $\mathrm {dp}(\sigma _r)$ is also increasing for $\sigma _r$. A double-minus configuration $\mathrm {dm}(\sigma _r) \in \{0,1\}^{E({\mathbb {H}})}$ is defined in a similar way and is also positively correlated under $\mu _{\mathscr {D}}$. We want to stress however that the union $\mathrm {dp}(\sigma _r) \cup \mathrm {dm}(\sigma _r)$ is not necessarily positively correlated.

**Fig. 6:**
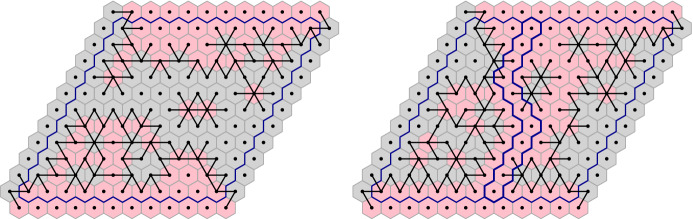
A configuration $\sigma _r$ of red spins ( is depicted in red,  in gray) on $\mathrm {Int}(\mathsf{Par}_{n,n})$ with the configuration $\zeta$ on $\partial _\mathrm {in}\mathsf{Par}\cup \partial _\mathrm {out}\mathsf{Par}$. In the left image no simple- crossing exists between the red arcs $\mathsf{Top}$ and $\mathsf{Bottom}$. The right image is $\tau (\sigma _r) \cup \zeta$; it contains a simple- crossing between these arcs. Observe that $\theta (\tau (\sigma _r) \cup \zeta )$ is the reflection of $\theta (\sigma _r \cup \zeta )$ with respect to the diagonal of the rhombus. In this concrete example $k[\theta (\sigma _r\cup \zeta )] - k[\theta (\tau (\sigma _r)\cup \zeta )]=1$ because the clusters of $\mathsf{Top}$ and $\mathsf{Bottom}$ are linked together after the reflection but not before

Write $\mathsf{Par}_{m,n}$ for the set of faces of ${\mathbb {H}}$ with centres at $k + \ell e^{i\pi /3}$ with $0 \le k \le m$ and $0 \le \ell \le n$ (see Figure 6). They form a domain approximately shaped as a parallelogram. Its boundary $\partial _E \mathsf{Par}_{m,n}$ may be partitioned into four sides called $\mathsf{Bottom}(m,n)$, $\mathsf{Right}(m,n)$, $\mathsf{Top}(m,n)$ and $\mathsf{Left}(m,n)$, defined as their name indicates. To be precise, $\mathsf{Right}(m,n)$ and $\mathsf{Left}(m,n)$ start and end with vertical edges. Below we will also use the notation $\mathsf{Bottom}(m,n)$, $\mathsf{Right}(m,n)$, $\mathsf{Top}(m,n)$ and $\mathsf{Left}(m,n)$ to refer to the faces of $\partial _\mathrm {in}\mathsf{Par}_{m,n} \cup \partial _\mathrm {out}\mathsf{Par}_{m,n}$ that are adjacent to these sections of $\partial _E\mathsf{Par}_{m,n}$. Faces in the corners of $\mathsf{Par}_{m,n}$ belong to two such sets.

Write  (and ) for the event that there exits a face-path in $\mathsf{Par}_{m,n}$ formed only of faces with spin , with the first face adjacent to the left side of $\mathsf{Par}_{m,n}$ and the last face adjacent to the right side (and top and bottom sides, respectively). Call such face-paths horizontal (respectively, vertical) *simple-* *crossings* of $\mathsf{Par}_{m,n}$.

Write  (and ) for the event that there exits a path of edges of $\mathrm {dp}(\sigma _r)$ contained in $\mathsf{Par}_{m,n}$ with one endpoint on the left side of $\mathsf{Par}_{m,n}$ and the other on the right side (and top and bottom sides, respectively); for technical reasons, we ask that the endpoints of the paths not be corners of $\mathsf{Par}_{m,n}$. We call such paths horizontal (and vertical, respectively) *double-* *crossings* of $\mathsf{Par}_{m,n}$.

The Russo-Seymour-Welsh (or RSW for short) theory first appeared in the simultaneous works of Russo and Seymour and Welsh for Bernoulli percolation [36, 38]. Its ultimate conclusion is that rectangles are crossed with probability bounded by constants that only depend on the rectangles’ aspect-ratios, not their sizes. Such crossing probability bounds were obtained for Bernoulli percolation using two separate arguments:

- a self-duality argument proves that the probability of crossing a square of any size is 1/2 (or more generally bounded uniformly away from 0);
- the so-called RSW lemma proves that crossing a rectangle of aspect ratio 2 in the long direction is bounded by a function of the probability of crossing a square of (roughly) the same size.

The same two step procedure will be used below for the double- percolation. While for bond percolation on ${\mathbb {Z}}^2$ with parameter 1/2 the first point is immediate due to self-duality, in our context a more complex argument is needed. The second point also requires special attention, due to the lack of independence and even of a general Spatial Markov property. A weak version of the RSW lemma is obtained easily using a general argument due to Tassion [42] (see Proposition 3.10 below). A more elaborate statement is proved later on (see Proposition 4.7); it requires considerable work.

#### Crossings of symmetric domains

This part contains results on crossing of symmetric domains; they are akin to the consequences of self-duality for site percolation on the triangular lattice or bond percolation on ${\mathbb {Z}}^2$. Two type of crossings will be treated: simple- crossings and double- crossings. We start with the former, where self-duality applies as for percolation.

##### Lemma 3.5

Let ${\mathscr {D}}$ be a domain containing $\mathsf{Par}_{n,n} \cup \partial _\mathrm {out}\mathsf{Par}_{n,n}$ for some *n*. Let  be such that  on $\mathsf{Bottom}(n,n)\cup \mathsf{Top}(n,n)$. Then3.3

##### Proof

Fix ${\mathscr {D}}$, $\zeta$ and *n*. Drop *n* from the notation $\mathsf{Par}$, $\mathsf{Bottom}$ and $\mathsf{Top}$. First observe that by the monotonicity in boundary conditions (Corollary 2.12*(i)*), the LHS of (3.3) is minimal when  on ${\mathscr {D}}\setminus (\mathsf{Bottom}(n,n)\cup \mathsf{Top}(n,n))$. We will assume this to be the case. All faces of $\mathsf{Bottom}(n,n)\cup \mathsf{Top}(n,n)$ have spin  in $\zeta$ as required by the proposition; we will switch the sign of the two left-most faces of $\mathsf{Top}(n,n)$ to  – this only decreases further the LHS of (3.3).

For $\sigma _r$ a red spin configuration on $\mathrm {Int}(\mathsf{Par})$, write $\sigma _r \cup \zeta$ for the configuration on ${\mathscr {D}}$ obtained by completing $\sigma _r$ with $\zeta$ on ${\mathscr {D}}\setminus \mathrm {Int}(\mathsf{Par})$. Write $\tau (\sigma _r)$ for the configuration obtained by applying the symmetry with respect to the line $e^{i\pi /6}{\mathbb {R}}$ to $-\sigma _r$. It is a known fact (see duality of site-percolation on ${\mathbb {T}}$ [28, Sec. 1.2]) that either  or .

Recall from Proposition 2.7 that  is proportional to $2^{k(\theta (\sigma _r\cup \zeta ))}$. Also notice that $\theta (\tau (\sigma _r) \cup \zeta )$ is easily determined in function of $\theta (\sigma _r\cup \zeta )$. Indeed, the configuration $\zeta$ restricted to $\partial _\mathrm {in}\mathsf{Par}\cup \partial _\mathrm {out}\mathsf{Par}$ is (almost) invariant under $\tau$. Thus, $\theta (\tau (\sigma _r) \cup \zeta )$ restricted to $\mathsf{Par}\cup \partial _\mathrm {out}\mathsf{Par}$ is the reflection with respect to the line $e^{i\pi /6}{\mathbb {R}}$ of $\theta (\sigma _r \cup \zeta )$. See Figure 6.

All other edges have same state in $\theta (\tau (\sigma _r) \cup \zeta )$ and $\theta (\sigma _r \cup \zeta )$: they are determined by $\zeta$ and are quite simple. Indeed, in $\theta (\zeta )$, all faces except those in the corners of $\partial _\mathrm {out}\mathsf{Par}$ are isolated points. Moreover, the top corners of $\partial _\mathrm {out}\mathsf{Par}$ are connected, as are the bottom ones.

It follows that, for any $\sigma _r$, $| k[\theta (\sigma _r\cup \zeta )] - k[\theta (\tau (\sigma _r)\cup \zeta )] | \le 1.$ Thus

3.4 $$\begin{aligned} \mu _{\mathscr {D}}( \sigma _r\cup \zeta ) \ge \tfrac{1}{2}\, \mu _{\mathscr {D}}( \tau (\sigma _r)\cup \zeta ). \end{aligned}$$

By summing the above over  we findThis proves the desired bound. $\quad \square$

Next we turn to double- crossings. The absence of such a crossing does not induce the existence of a double- crossing, and we may not apply the same argument as above. We do however have a similar statement.

##### Lemma 3.6

For any $m,n \ge 1$, and any pair of coherent configurations  and , either $\mathsf{Par}_{m,n}$ is crossed horizontally by a double path of constant red spin, or it is crossed vertically be a double path of constant blue spin. That is3.5

##### Proof

Recall that $\mathrm {dp}(\sigma _r),\mathrm {dm}(\sigma _r),\mathrm {dp}(\sigma _b),\mathrm {dm}(\sigma _b)\subset E(\mathsf{Par}_{m,n})$ denote the sets of double plus and double minus edges in $\sigma _r$ and $\sigma _b$, respectively. Also, recall that to each edge $e\in E({\mathbb {H}})$ we associate its dual $e^*\in E({\mathbb {T}})$ that is defined as the unique edge on ${\mathbb {T}}$ that intersects *e*. For a set $S\subset E({\mathbb {H}})$ we denote by $S^*\subset {\mathbb {T}}$ the set of edges dual to the edges in *S*.

By duality between ${\mathbb {H}}$ and ${\mathbb {T}}$, either $\mathrm {dp}(\sigma _r)\cup \mathrm {dm}(\sigma _r)$ contains a left-right crossing of $\mathsf{Par}_{m,n}$, or $\left[ E(\mathsf{Par}_{m,n})\setminus (\mathrm {dp}(\sigma _r)\cup \mathrm {dm}(\sigma _r))\right] ^*$ contains a top-bottom crossing of $\mathsf{Par}_{m,n}$.

First consider the case when $\mathrm {dp}(\sigma _r)\cup \mathrm {dm}(\sigma _r)$ contains a left-right crossing of $\mathsf{Par}_{m,n}$. Any such crossing consists either entirely of edges of $\mathrm {dp}(\sigma _r)$ or entirely of edges of $\mathrm {dm}(\sigma _r)$. Indeed, edges of $\mathrm {dp}(\sigma _r)$ and $\mathrm {dm}(\sigma _r)$ can never share a vertex.

In conclusion, in this case at least one of  and  occurs.

It remains to consider the case when $\left[ E(\mathsf{Par}_{m,n})\setminus (\mathrm {dp}(\sigma _r)\cup \mathrm {dm}(\sigma _r))\right] ^*$ contains a top-bottom crossing of $\mathsf{Par}_{m,n}$. Let $\gamma ^*$ be such a crossing.

For each edge $e\in E({\mathscr {D}})$, let $N(e)\subset E(\mathsf{Par}_{m,n})$ denote the set of edges consisting of *e* and all edges in $E(\mathsf{Par}_{m,n})$ that share a vertex with *e*. Then, if $e^*\in \gamma ^*$, we claim that $N(e)\subset \mathrm {dp}(\sigma _b)\cup \mathrm {dm}(\sigma _b)$. Indeed, the two faces of  $\mathsf{Par}_{m,n}$ separated by *e* have opposite red spin, hence same blue spin. Moreover, the blue spins of the two faces adjacent the endpoints of *e* but not containing *e* in their boundary must also coincide with the spins on either side of *e*.

It remains to observe that the union of *N*(*e*) taken over all *e* such that $e^*\in \gamma ^*$ contains a top-bottom crossing of $\mathsf{Par}_{m,n}$. Thus $\mathrm {dp}(\sigma _b)\cup \mathrm {dm}(\sigma _b)$ contains a top-bottom crossing of $\mathsf{Par}_{m,n}$, and thus either  or  occurs. $\quad \square$

##### Remark 3.7

It is obvious from the proof that Lemma 3.6 may be generalised to other domains with four arcs marked on the boundary.

Later we will also use the fact that, if an annulus $\Lambda _N \setminus \Lambda _n$ does not contain a circuit around $\Lambda _n$ of either double- or double-, then $\Lambda _n$ is connected to $\Lambda _N^c$ by a double-path of constant blue spin.

##### Lemma 3.8

For any $n \ge 1$ and any domain ${\mathscr {D}}$ containing $\mathsf{Par}_{n,n}$ and symmetric with respect to one of the diagonals of $\mathsf{Par}_{n,n}$,

##### Proof

By Lemma 3.6 we haveIn the equality we used the fact that the blue spin marginal of  is  and that  ; in the last line we used that . This provides the desired result. $\quad \square$

##### Corollary 3.9

For any $$n \ge 1$$

##### Proof

For any domain ${\mathscr {D}}$ as in Lemma 3.8, by the monotonicity of boundary conditions,By taking the limit of the above as ${\mathscr {D}}$ grows to ${\mathbb {H}}$, we obtain the desired bound. $\quad \square$

#### Sub-sequential RSW

##### Proposition 3.10

(RSW). We have3.6As a consequence .

The proof of Proposition 3.10 uses a technique introduced by Tassion in [42]. Indeed, the main argument of [42, Thm. 1] shows that  (which is the result of Corollary 3.9) implies (3.6). This technique applies to general percolation measures with the FKG property and sufficient symmetry. Our model fits in this framework and the relevant part of the proof of [42, Thm. 1] applies readily. We simply point out that, in order to harness the symmetries of the hexagonal lattice, one should apply the argument using crossings of hexagonal domains between opposite sides, rather than crossings of squares or lozenges.

Note that [42, Thm. 1] actually claims a stronger result than (3.6), where $\limsup$ is replaced by $\liminf$. This improvement requires an additional ingredient which is lacking here. For now we are content with the above sub-sequential form of RSW.

A stronger statement (with the lower bound valid for all *n*) will be proved in Section 4 – see Proposition 4.7. All the ingredients for it are already available, however the proof is tedious and is not necessary at this point. The argument of [42] is elegant, short and quite robust, and suffices to prove Theorem 3.2; we prefer it for now.

##### Proof

The argument of [42, Thm. 1] requires minor modifications because the hexagonal lattice in invariant under rotations of $\pi /3$, unlike the square one, which is invariant under rotations of $\pi /2$. We briefly sketch the adapted argument below.

Write $\mathsf{T}$ and $\mathsf{B}$ for the top and bottom horizontal sections of $\partial \Lambda _n$, and let  be the event that $\mathsf{T}$ and $\mathsf{B}$ are connected to each other by a double- path contained in $\Lambda _n$. From Corollary 3.9, using standard applications of the FKG inequality and the invariance of $\Lambda _n$ under rotations by multiples of $\pi /3$, we deduce that  is bounded away from 0 uniformly in *n*.

Following [42], define $2\alpha _n$ as the maximal width of a centred interval *I* on $\mathsf{B}$ such that

**Fig. 7:**
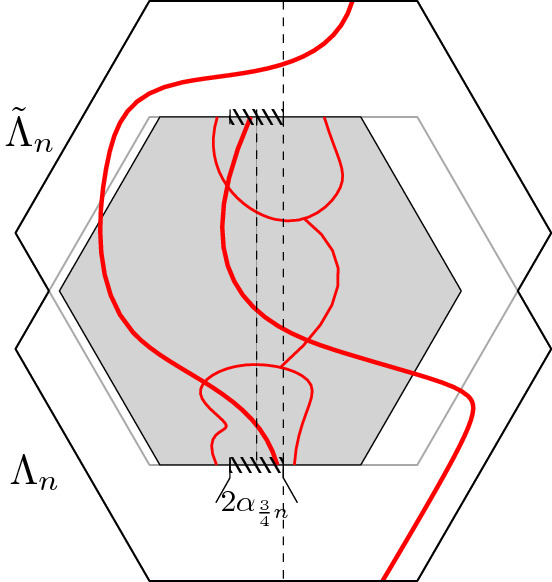
The construction that proves that if $\alpha _{n} \le \frac{1}{2} \alpha _{3n/4}$, then $\Lambda _n \cup {{\tilde{\Lambda }}}_{n}$ is crossed vertically with uniformly positive probability

Call ${{\tilde{\Lambda }}}_n$ the vertical translate of $\Lambda _n$ by *n*/2 and ${\widetilde{\mathsf{T}}}$ the corresponding translate of $\mathsf{T}$. Then, using the same argument as in [42, Lemma 2.2] (see also Figure 7), $\alpha _{n} \le 2 \alpha _{3n/4} \le n/4$ implies thatfor some constant $c> 0$ independent of *n*. Through additional standard applications of the FKG inequality, the above implies in turn that  and  are bounded below by strictly positive quantities depending only on *c*. Moreover, if $\alpha _n > n/4$, then lower bounds on  and  follow by simple considerations, similar to those of the start of the proof of [42, Lemma 2.2].

Finally, since $0\le \alpha _n \le n$ for all *n*, there exist infinitely many values of *n* such that $\alpha _{n} \le 2 \alpha _{3n/4}$, and the proof is complete. $\quad \square$

##### Corollary 3.11

Under , 0 is surrounded a.s. by an infinite number of disjoint circuits of double-.

##### Proof

Suppose the opposite, that is that with positive -probability, 0 is surrounded by a finite number of disjoint double- circuits. Since  is ergodic and the above event is translation invariant, it occurs with probability 1.

Set ; observe that *N* is a random variable that is, by our assumption, -a.s. finite. Then there exists $n_0$ such thatsince the right-hand side is strictly positive by Proposition 3.10. Using that, for all $n>n_0$,we obtain a contradiction. $\quad \square$

##### Corollary 3.12

The graph $\theta (\sigma _r)$ contains -a.s. no infinite cluster.

##### Proof

Observe that a double--circuit in $\sigma _r$ blocks connections in $\theta (\sigma _r)$. More precisely, if , then all clusters of $\theta (\sigma _r)$ that intersect $\Lambda _n$ are finite. Now Corollary 3.11 states that  for all *n*, which implies by the observation above that $\theta (\sigma _r)$ contains no infinite cluster a.s. $\quad \square$

### Joint infinite-volume measure

We now turn to the existence of limiting measures for the joint law of the red and blue spins, that is Theorem 3.1.

The crucial property here is that by Lemma 2.7, conditionally on $\sigma _r$, $\sigma _b$ is obtained by colouring independently and uniformly the clusters of $\theta (\sigma _r)$ in either  or . This procedure may also be applied in infinite-volume for red-spin configurations sampled according to . The absence of infinite clusters in $\theta (\sigma _r)$ is used to show that the result of this procedure in a finite but large volume is close to that in infinite-volume.

#### Proof of Theorem 3.1

Let  $({\mathscr {D}}_n)_{n \ge 0}$ be an increasing sequence of domains with $\bigcup _{n\ge 0} {\mathscr {D}}_n = {\mathbb {H}}$. Recall from Theorem 3.4 that the red-spin marginals of  converge to an ergodic translation-invariant limiting measure denoted by . Let  be the measure obtained by sampling $\sigma _r$ according to , then awarding to all faces of each cluster of $\theta (\sigma _r)$ a blue spin uniformly chosen in , independently for each cluster. Let us prove that  converges to .

Fix $k\in {\mathbb {N}}$ and $\epsilon >0$. We will show that the total-variation distance between the restrictions of  and  to $\Lambda _k$ is smaller than $2\epsilon$, provided that *n* is large enough.

Let $K \ge k$ be such that . Due to Corollary 3.12, it is always possible to choose *K* with this property.

Now, let $N = N(\epsilon ,K)$ be such that, for any $n \ge N$, the distance in total variation between the restrictions of  and  to $\Lambda _K$ is smaller than $\epsilon$. Thus, one may couple  and  to produce configurations $\sigma _r, \sigma _r'$ in such a way $\sigma _r = \sigma _r'$ on $\Lambda _K$ with probability at least $1- \epsilon$. Moreover, by choice of *K*, with probability at least $1 - 2\epsilon$, $\sigma _r = \sigma _r'$ on $\Lambda _K$ and there is no connected component of $\theta (\sigma _r)$ that intersects both $\Lambda _k$ and $\Lambda _K^c$. On this event, the connected components of $\theta (\sigma _r)$ and $\theta (\sigma _r')$ that intersect $\Lambda _k$ are identical. Using the same blue spin assignment for these components, we have produced a coupling of  and  that is equal inside $\Lambda _k$ with probability at least $1- 2\epsilon$, which was our goal.

Since $\epsilon > 0$ and *k* are arbitrary, we conclude that   converges to .

The translation invariance of  follows from that of . Since  is invariant under rotations by multiples of $\pi /3$ and converges to , the latter is also invariant under such rotations. The ergodicity of  follows from that of  and from the absence of infinite clusters in $\theta (\sigma _r)$. $\quad \square$

#### Proposition 3.13

Under , $\sigma _b$ contains a.s. no infinite -cluster and no infinite -cluster. As a consequence, $\omega _b$ is formed entirely of finite loops -a.s.

The proof below is a straightforward application of the uniqueness argument of Burton and Keane [5] and of Zhang’s argument for non-coexistence of clusters (see [23, Lem 11.12] for an illustration of this argument which was never published by Zhang himself).

#### Proof

To start, observe that under  the number  of infinite -clusters is a.s. constant. This is a direct consequence of the ergodicity of $\sigma _b$ under this measure. The same applies to infinite -clusters.

The technique introduced by Burton–Keane in [5] applies readily to the blue-spin marginal under . Indeed, this marginal satisfies the finite-energy property required by [5]. As a consequence we obtain that either  -a.s. or  -a.s.

Finally, let us prove that   -a.s. by contradiction. Assume that  -a.s. Then, by the symmetry of the blue-spin marginal, the number of -infinite clusters is also equal to 1 a.s. Thus, there exists some $n \ge 1$ such that . Write $\partial _1\Lambda _n,\dots , \partial _6 \Lambda _n$ for the six sides of $\partial \Lambda _n$ in counter-clockwise order. ThenThe inequality is due to the FKG property for $\sigma _b$ and to the invariance of the measure under rotations by $\pi /3$. Thus we findThe same holds for any side of $\Lambda _n$ and also for -connections instead of  ones.

Define the event  and . Using the union bound, we findNow notice that when the above event occurs, then necessarily either there exist two infinite -clusters or two infinite -clusters. This contradicts the uniqueness of the infinite cluster proved above.

Finally, the existence of an infinite path in $\omega _b$ implies the existence of both infinite  and  clusters, which was excluded above. $\quad \square$

### Uniqueness of infinite-volume measure: proof of Theorem 3.2

#### Proof of Theorem 3.2

Let us first prove that -a.s., there exist infinitely many loops surrounding the origin in the loop representation of $(\sigma _r,\sigma _b)$. To that end, it is enough to show that for any *n*, -a.s. there exists at least one loop surrounding $\Lambda _n$. Fix *n* and consider the union of all - and -clusters that intersect $\Lambda _n$. Due to Proposition 3.13, all these clusters are finite. The outer boundary of their union is then a finite blue loop surrounding $\Lambda _n$. Hence, 0 is surrounded a.s. by infinitely many loops.

Let us now prove (3.1). Fix *n*. By the above, -a.s. there exist infinitely many loops surrounding $\Lambda _n$ which may be ordered starting from the inner most. Since each loop is blue or red with probability 1/2 independently, there exist a.s. four consecutive loops $\gamma _1,\dots , \gamma _4$ surrounding $\Lambda _n$ that have colours red, blue, red, blue, in this order, from inside out. Then both $\gamma _1$ and $\gamma _3$ have constant blue spins on all faces adjacent to them, but that for $\gamma _1$ is opposite to that for $\gamma _3$. That is, either $\gamma _1$ is double- and $\gamma _3$ is double- or $\gamma _1$ is double- and $\gamma _3$ is double-. Similarly, of $\gamma _2$ and $\gamma _4$, one is double- and the other is double-. This proves (3.1).

A direct consequence of  is that the restriction of  to $\Lambda _n$ is dominated by . Thus . Moreover, due to the monotonicity of boundary conditions, for any sequence of finite domains ${\mathscr {D}}_n$ that increases to ${\mathbb {H}}$, the measures  and , as well as the red-spin marginals of , ,  and  all converge to .

Finally, due to the procedure that selects blue spins knowing the red spins, we conclude that  for all boundary conditions  . $\quad \square$

## A Dichotomy Theorem

Below we state a dichotomy result similar to those of [17] and [18]. The result states that the model is in one of two states: co-existence of phases (see case *(i)* of Corollary 4.2) or (stretched)-exponential decay of diameters for clusters of one phase inside the other (see case *(ii)*). In Section 5, we show that the latter case contradicts Theorem 3.2.

Compared to the setting of [17] and [18], the present model exhibits considerable additional difficulties due to the lack of a general Spatial Markov property and to the absence of monotonicity in the boundary conditions . These difficulties appear in several places, most notably in proving a crossing estimate inside mixed boundary conditions (Corollary 4.14) and in eliminating case *(ii)*.

### Theorem 4.1

There exist constants $\rho > 2$ and $C>1$ such that, if for $n\ge 1$ we set , we have

4.1 $$\begin{aligned} \alpha _{(\rho +2)n} \le C \alpha _n^2 \qquad \qquad \text {for all }n\ge 1. \end{aligned}$$

### Corollary 4.2

For $\rho > 2$ given by the above, one of the two following statements holds

(i) $$\inf _n \alpha _n >0$$ or
(ii) there exist constants $c,C>0$ and $n_0 \ge 1$ such that $\alpha _n \le C e^{-n^c}$ for all $n = (\rho +2)^k n_0$ with $k\in {\mathbb {N}}$.

The constant $\rho$ in Theorem 4.1 will be chosen large enough to accommodate certain geometric constructions used in the proof. Its choice only affects scenario (ii) of Corollary 4.2, which we will see is contradictory. Thus, any value of $\rho$ suffices for our purposes.

### Preparation: measure in cylinder

**Fig. 8:**
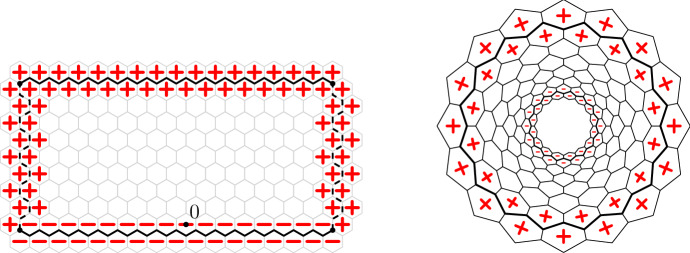
Left: The rectangle $\mathsf{Rect}_{m,n}$ with boundary conditions . The dashed lines are the left and right boundaries; in $\mathsf{Cyl}_{m,n}$ they are identified to each other. Right: The cylinder $\mathsf{Cyl}_{m,n}$ may be drawn in the plane as depicted; its top and bottom are marked by bold lines. The resulting graph is trivalent, all its faces except the interior one are hexagons, and their union is simply connected. The FKG lattice condition may be proved for this graph in the same way as for domains of the hexagonal lattice. The measure  is obtained as a conditioning of the measure on the depicted graph: all faces adjacent to the bottom have red spin , while all those adjacent to the top have red spin

Write $[a,b] \times [c,d]$ for the set of faces of ${\mathbb {H}}$ with centres inside $[a,b] \times [c,d]$. Any such rectangle is a domain of ${\mathbb {H}}$ and we will treat it as such. Its boundary may be split into four segments: bottom, top, left and right. We do not give precise definitions, but mention that the left and right sections start and end with vertical edges (see Figure 8 for an illustration).

For $n,m \in {\mathbb {N}}$, let $\mathsf{Rect}_{m,n}$ be the rectangle $[-m,m - \frac{1}{2}] \times [0,n]$. Write  for the boundary conditions on $\mathsf{Rect}_{m,n}$ where all faces adjacent to the bottom of $\partial \mathsf{Rect}_{m,n}$ have red spin  and all other faces adjacent to  $\partial \mathsf{Rect}_{m,n}$ have red spin . As for other boundary conditions, these may be defined only on $\mathsf{Rect}_{m,n}$, with no reference to the outside faces. One may however check that, since there are only two arcs of different sign on the boundary, these boundary conditions do satisfy the Spatial Markov property.

We will also consider the cylinder $\mathsf{Cyl}_{m,n}$ obtained by identifying the left and right boundaries of $\mathsf{Rect}_{m,n}$. Write  for the boundary conditions on $\mathsf{Cyl}_{m,n}$ which are double- on the bottom and double- on the top. That is  is the uniform measure on pairs of coherent spin configurations $(\sigma _b,\sigma _r)$ on $\mathsf{Cyl}_{m,n}$ with the property that all faces adjacent to the top boundary of $\mathsf{Cyl}_{m,n}$ have  and all those adjacent to the bottom have . No restriction on the blue spins of the boundary faces is imposed.

It is immediate that the Spatial Markov property applies to  in the same way as for planar domains. In particular,   is related to  by the following.

#### Lemma 4.3

Fix $m,n \ge 1$ and let $\mathsf {L_v}$ be the right boundary of $\mathsf{Rect}_{m,n}$. Then $\mathsf {L_v}$ is also an edge-path of $\mathsf{Cyl}_{m,n}$, and

While $\mathsf{Cyl}_{m,n}$ is not a planar domain, the FKG inequality applies to it.

#### Lemma 4.4

For any $m,n \ge 1$, the red-spin marginal of  satisfies the FKG lattice condition and is positively associated.

#### Proof

We will not give a full proof of this, only a sketch. Notice that $\mathsf{Cyl}_{m,n} \cup \partial _\mathrm {out}\mathsf{Cyl}_{m,n}$, may be embedded in the plane and rendered simply connected by adding a face of degree 4*m* below the bottom of $\partial _\mathrm {out}\mathsf{Cyl}_{m,n}$, as drawn in Figure 8, right diagram. Write ${\mathscr {D}}$ for the planar graph obtained by this procedure. Then a straightforward adaptation of Theorem 2.6 (see also remark 2.11*(ii)*) shows that the FKG lattice condition also holds for $\mu _{\mathscr {D}}$.

As explained in Corollary 2.10, conditioning on the value of red spins on a given set conserves the FKG lattice condition for the red spin marginal. In particular, the red-spin marginal of $\mu _{\mathscr {D}}$ conditioned on the event that all faces adjacent to the top of $\mathsf{Cyl}_{m,n}$ have  while all those adjacent to the bottom have   also satisfies the FKG lattice condition. Finally, the Spatial Markov property states that the conditional measure above is identical to  (when restricted to $\mathsf{Cyl}_{m,n}$). $\quad \square$

### Strong RSW theory

As promised in Section 3.2.2, we will now prove a stronger RSW result. Variations of it may be envisioned; we will state it in the form most useful to us. We start with a general lemma that allows to lengthen crossings of long rectangles. The main result of the section (Proposition 4.7) is given afterwards. It will be stated and proved for the cylinder, but may also be deduced in other settings.

#### Lengthening crossings

Recall the definition of $\mathsf{Par}_{m,n}$ and its boundary segments $\mathsf{Top}$ and $\mathsf{Bottom}$ from Section 3.2.

##### Lemma 4.5

Let ${\mathscr {D}}$ be a domain and *n* be such that $\mathsf{Par}_{3n,n} \cup \partial _\mathrm {out}\mathsf{Par}_{3n,n} \subset {\mathscr {D}}$. Fix some red spin configuration $\zeta$ on ${\mathscr {D}}$ with the property that all faces of $\mathsf{Bottom}(3n,n) \cup \mathsf{Top}(3n,n)$ are awarded spins . Then4.2

The previous lemma may be used to glue crossings of long rectangles as described below.

##### Corollary 4.6

Let ${\mathscr {D}}$ be a domain such that $\mathsf{Par}_{5n,n}\subset {\mathscr {D}}$. Then4.3where $(n,0) + \mathsf{Par}_{4n,n}$ is the translate of $\mathsf{Par}_{4n,n}$ by *n* units to the right.

Due to the Spatial Markov property, the statements of Lemma 4.5 and Corollary 4.6 also apply to measures with boundary conditions such as .

**Fig. 9:**
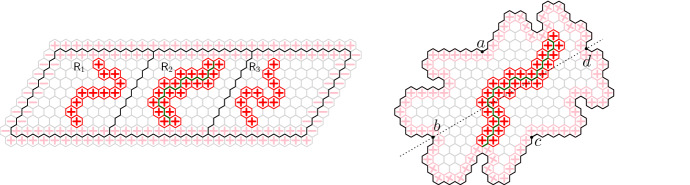
*Left:* The parallelogram  $\mathsf{Par}_{3n,n}$ contains three disjoint translations ${\mathsf {R}}_1$, ${\mathsf {R}}_2$ and ${\mathsf {R}}_3$ of  $\mathsf{Par}_{3n,n}$. Given the outside configuration $\zeta$, ${\mathsf {R}}_1$ and ${\mathsf {R}}_3$ contain simple- vertical crossings with probability at least 1/2. Conditionally on the existence of such crossings, ${\mathsf {R}}_2$ contains a vertical double- crossing with probability at least 1/4. *Right:* The latter statement is proved by working in the symmetric domain $\mathsf{D}$, which is crossed vertically by a double- path with probability 1/4 at least

##### Proof of Lemma 4.5

By the FKG inequality, the LHS of (4.2) is minimal when  on ${\mathscr {D}}\setminus (\mathsf{Bottom}(3n,n) \cup \mathsf{Top}(3n,n))$. We may assume this below.

Observe that $\mathsf{Par}_{3n,n}$ may be partitioned into three translations of $\mathsf{Par}_{n,n}$; call them ${\mathsf {R}}_1$, ${\mathsf {R}}_2$ and ${\mathsf {R}}_3$ ordered from left to right (see Figure 9). Due to Lemma 3.5 and the FKG inequality,4.4If  occurs, let $\Gamma _L$ be the left-most simple- vertical crossing of ${\mathsf {R}}_1$. Formally, let $\Gamma _L$ be the edge-path running along the left side of the spin-crossing. Also, let $\Gamma _R$ be the right-most simple- vertical crossing of ${\mathsf {R}}_3$ when  occurs.

Fix two possible realisations $\gamma _L,\gamma _R$ of $\Gamma _L,\Gamma _R$. Let $\mathsf{D}_0$ be the domain formed of the faces of $\mathsf{Rect}_{3n,n}$ between $\gamma _L$ and $\gamma _R$. The events $\Gamma _L = \gamma _L$ and $\Gamma _R =\gamma _R$ are both measurable in terms of the configuration outside $\mathsf{D}_0$.

Consider the line $\ell$ running through the bottom left and upper right corner of ${\mathsf {R}}_2$ and let $\rho$ be the orthogonal symmetry with respect to $\ell$; note that ${\mathbb {H}}$ is invariant under $\rho$. Let $\mathsf{D}$ be the domain formed of the faces of $\mathsf{D}_0$ and those of $\rho (\mathsf{D}_0)$.

Let *a*, *b*, *c*, *d* be the corners of ${\mathsf {R}}_2$ ordered in counter-clockwise order, starting from the top-left corner. Write (*bc*) and (*da*) for the arcs of $\partial \mathsf{D}$ in counter-clockwise order. Then, due to the monotonicity in boundary conditions and the FKG inequality,The equality is due to the specific to the boundary conditions induced by $\Gamma _L$, $\Gamma _R$ and $\zeta$ on $\mathsf{D}_0$ (a brief analysis is needed to ensure that the is no multiplicative constant appearing between the two sides). The first inequality is due to the FKG inequality and the inclusion of events; the last one is due to Lemma 3.8. Averaging the above over all possible values of $\Gamma _L~$ and $\Gamma _R$ and using (4.4), we obtain the desired bound. $\quad \square$

##### Proof of Corollary 4.6

Let $\Gamma _L^+$ and $\Gamma _L^-$ be the top and bottom most, respectively, double- horizontal crossings of $\mathsf{Par}_{4n,n}$.

Define $\Gamma _R^+$ and $\Gamma _R^-$ similarly for the rectangle $(n,0) + \mathsf{Par}_{4n,n}$. When both $\mathsf{Par}_{4n,n}$ and $(n,0) + \mathsf{Par}_{4n,n}$ are crossed horizontally by double- paths, then either $\Gamma _L^+$ intersects or is higher than $\Gamma _R^-$ inside the middle parallelogram $(0,n)+\mathsf{Par}_{3n,n}$, or $\Gamma _L^-$ intersects or is lower than $\Gamma _R^+$. As a consequence,4.5In the last inequality we used the FKG property. We focus next on the first term in the LHS above.

For any realisation of $\Gamma _L^+$ and $\Gamma _R^-$ with the former intersecting or higher than the latter, if $\Gamma _L^+$ and $\Gamma _R^-$ intersect or if they are connected to each other by a double- path, then  occurs. Below we will show that, conditionally on $\Gamma _L^+$ and $\Gamma _R^-$, the two paths intersect or are connected by a double- path with positive probability. The case where the paths intersect is trivial; we assume henceforth that $\Gamma _L^+$ and $\Gamma _R^-$ are disjoint. Notice that $\Gamma _L^+$ is measurable in terms of the spins of the faces of $\mathsf{Par}_{4n,n}$ above it, and $\Gamma _R^-$ is measurable in terms of the spins of the faces of $(n,0) + \mathsf{Par}_{4n,n}$ below it. Let $\mathsf{U}$ be the set of all faces of $\mathsf{Par}_{5n,n}$ which are in neither of the two categories above.

Let *a* be the right endpoint of $\Gamma _L^+$ and *c* be the left endpoint of  $\Gamma _R^-$. Orient $\Gamma _L^+$ from left to right and $\Gamma _R^-$ from right to left. Let *b* be the last point of intersection of $\Gamma _L^+$ with the left side of  $(n,0) + \mathsf{Par}_{3n,n}$, and *d* be the last point of intersection of $\Gamma _R^-$ with the right side of  $(n,0) + \mathsf{Par}_{3n,n}$. Write $\mathsf{D}$ for the domain contained in $(n,0) + \mathsf{Par}_{3n,n}$, delimited at the top by the section of $\Gamma _L^+$ between *b* and *a*, and at the bottom by the section of $\Gamma _R^-$ between *d* and *c*.

Let *H* be the event that there exists an edge-path $\gamma$ contained in $\mathsf{D}$, connecting the arcs (*ab*) and (*cd*) of $\partial _E\mathsf{D}$, such that all faces of $\mathsf{U}\cap \mathsf{D}$ adjacent to it are of red spin . Then, by the FKG inequality and the properties *(i)* and *(ii)* of Lemma 2.13,The second inequality is due to the inclusion between the two events. Notice also that when *H* occurs, $\Gamma _L^+,\Gamma _R^-~$ are necessarily connected by a double- path.

Now, applying again Lemma 2.13, we conclude thatwhere $a',b',c'$ and $d'$ are the corners of the parallelogram, ordered in counter-clockwise order, starting from the top left. The RHS of the above is bounded below by 1/36, as proved in Lemma 4.5. Combining the last two displayed equations, we findAveraging over all values of $\Gamma _L^+$ and $\Gamma _R^-$ as above, we findThe same bound holds for the second term in (4.5), and (4.3) follows. $\quad \square$

**Fig. 10:**
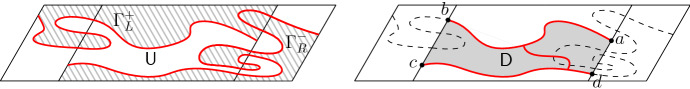
*Left:* the paths $\Gamma _L^+$ and $\Gamma _R^-$ are measurable in terms of the hashed regions; its complement is  $\mathsf{U}$. *Right:* The domain $\mathsf{D}$ is delimited by parts of $\Gamma _L^+$ and $\Gamma _R^-$. Any crossing between the arcs (*ab*) and (*cd*) in $\mathsf{D}$ induces a connection between $\Gamma _L^+$ and $\Gamma _R^-$

#### Statement of the strong RSW

The notions of simple and double horizontal and vertical crossings adapt readily to rectangular domains $[a,b]\times [c,d]$. Use the notations ${\mathscr {C}}^h_{.}([a,b]\times [c,d])$ and ${\mathscr {C}}^v_{.}([a,b]\times [c,d])$ for the existence of such crossings.

##### Proposition 4.7

(Strong RSW). There exists a function $\psi : (0,1] \rightarrow (0,1]$ such that, for all $N,M,n,k \ge 1$ with $n+k \le N$ and $2n < M$,4.6

In other words, the above tells us that if wide rectangles are crossed vertically with positive probability (that is in the easy direction), then they are also crossed horizontally (*i.e.* in the hard direction) with positive probability. This is a typical RSW result in that it relates probabilities of crossings in the easy direction to those of crossings in the hard direction. What is remarkable is that the measure to which it applies, namely , is not rotationally invariant. Thus, vertical crossings are “orthogonal” to horizontal ones. The exact aspect ratio of the two rectangles (4 and 6, respectively) is not essential; they have been chosen to simplify statements later on.

The proof of Proposition 4.7 is quite intricate. It is based on similar results from [18], but with additional difficulties due to the two layers of spins necessary for the Spatial Markov property (see Theorem 2.3). A very similar version appears in [14]. The next section is dedicated to proving Proposition 4.7.

#### Proof of Proposition 4.7

The structure is reminiscent of a proof by contradiction: assuming that (4.6) fails (more precisely that  has probability below a certain threshold), we prove that double- vertical crossings of $[-3n,3n] \times [k,k+n]$ have some particular behaviour. This is done in a series of lemmas (Lemmas 4.8, 4.9, 4.10 and 4.11) – the title of each lemma indicates a constraint that vertical crossings need to satisfy *for* (4.6) *to fail*. Then we prove that two typical vertical crossings of $[-3n,3n] \times [k,k+n]$ with starting points sufficiently close to each other are connected with positive probability by a double- path (see Lemma 4.12). Proposition 4.7 follows from this last statement. Lemma 4.12 is the heart of the proof; it relies on the construction of a symmetric domain, similarly to what was done for Lemma 4.5.

Fix *n*, *k*, *M*, *N* as in the proposition. We will work with *n* large; the function $\psi$ in the proposition may be adjusted to incorporate all small values of *n*. For ease of writing, translate the cylinder $\mathsf{Cyl}_{M,N}$ vertically by $-k$; write $\mu$ for the measure with boundary conditions  on this translated cylinder.

**Fig. 11:**
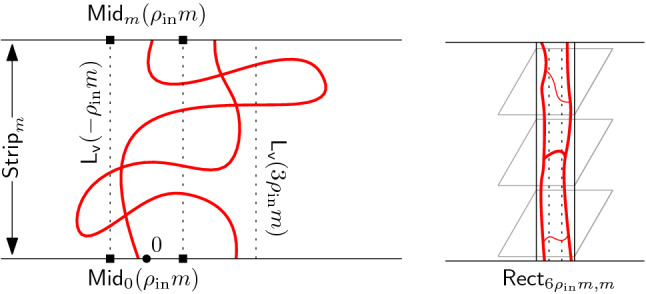
*Left:* Using two symmetric instances of ${\mathscr {H}}_\mathsf {wig}^\mathrm {in}(m)$ we create a vertical crossing of the thin rectangle $[-\rho _\mathrm {in}m,3\rho _\mathrm {in}m] \times [0,m]$. *Right:* When both $[-6\rho _\mathrm {in},-2\rho _\mathrm {in}m]\times [0,m]$ and $[2\rho _\mathrm {in},6\rho _\mathrm {in}m]\times [0,m]$ are crossed vertically by double- paths, then these paths may be connected as in Lemma 4.5: first by simple- paths (thin red lines), then by a double- path (bold red line)

Using this notation, our goal is to prove that, for any $C > 0$ there exists $\Delta > 0$ depending only on *C*, not on *n*, *k*, *M* or *N*, such thatFor $m,h\ge 0$, write $\mathsf {L_v}(m)$ for the right boundary of the rectangle $\mathsf{Rect}_{m,N}$ and $\mathsf {L_h}(h)$ for the top boundary of $\mathsf{Rect}_{M,h}$. That is $\mathsf {L_h}(h)$ is approximately a horizontal line at height *h*; $\mathsf {L_v}(m)$ is a vertical line *m* units to the right of 0. Let $\mathsf{Strip}_h$ be the set of faces of the cylinder contained between $\mathsf {L_h}(0)$ and $\mathsf {L_h}(h)$. Define $\mathsf {Mid}_h(m)$ as the segment of $\mathsf {L_h}(h)$ contained in $[- m,m] \times {\mathbb {R}}$. See Figure 11 for illustrations.

Below we will talk about double- paths contained in $\mathsf{Strip}_h$ with endpoints in $\mathsf {Mid}_0(m)$ and $\mathsf {Mid}_h(m)$, respectively. While not explicitly stating it each time, we will always ask that such a path have trivial winding around the cylinder.

##### Lemma 4.8

(Endpoints of paths are centred). For any $\epsilon > 0$ and $C_{\mathsf {mid}} >0$ there exists $\Delta _{\mathsf {mid}} =\Delta _{\mathsf {mid}}(\epsilon ,C_{\mathsf {mid}}) >0$ such that the following holds. Fix $m \le n$ and write ${\mathscr {H}}_\mathsf {mid}(m)$ for the event that there exists a double- path in $\mathsf{Strip}_m$ with one endpoint in $\mathsf {Mid}_0(m \epsilon )$ and one in $\mathsf {L_h}(m)\setminus \mathsf {Mid}_{m}(2 m\epsilon )$. Then4.7

##### Proof

Fix $C_\mathsf {mid}$ and suppose $\mu [{\mathscr {H}}_\mathsf {mid}(m)] > C_{\mathsf {mid}}$. Then, by symmetry, with probability at least $C_\mathsf {mid}/2$ there exists a double- path in $\mathsf{Strip}_m$ with lower endpoint in $\mathsf {Mid}_0(m\epsilon )$ and upper end-point on $\mathsf {L_h}(m)$, to the right of $\mathsf {L_v}(2\epsilon m)$. By horizontal symmetry and translation invariance, the event that there exists a double- path in $\mathsf{Strip}_m$ with lower endpoint in $(4\epsilon m,0) + \mathsf {Mid}_0(m\epsilon )$ and upper end-point on $\mathsf {L_h}(m)$, to the left of $\mathsf {L_v}(2\epsilon m)$ also has probability greater than $C_\mathsf {mid}/2$. Notice that when these two events occur, then the segments $\mathsf {Mid}_0(m\epsilon )$ and $(4\epsilon m,0) + \mathsf {Mid}_0(m\epsilon )$ are connected by a double- path contained in $\mathsf{Strip}_m$. Using the FKG inequality and the above considerations, we find4.8For $j \in {\mathbb {Z}}$, let $M_j = (2j\epsilon m,0) + \mathsf {Mid}_0(m\epsilon )$. Using again the invariance of $\mu$ under horizontal shift, we find thatFinally, if all the events above occur simultaneously, then  also occurs. By the FKG inequality, we find$\square$

##### Lemma 4.9

(Vertical paths wiggle). There exist explicit constants $0< \rho _\mathrm {in}< \rho _\mathrm {out}$ with the following property. For $m \le n$ define the events

- ${\mathscr {H}}_\mathsf {wig}^\mathrm {in}(m)$ is the event that there exists a double- path contained in $\mathsf{Strip}_m$, from $\mathsf {Mid}_{0}(\rho _\mathrm {in}m)$ to $\mathsf {Mid}_{m}(\rho _\mathrm {in}m)$ that does not cross $\mathsf {L_v}(\rho _\mathrm {in}m)$ or that does not cross $\mathsf {L_v}(-\rho _\mathrm {in}m)$;
- ${\mathscr {H}}_\mathsf {wig}^\mathrm {out}(m)$ is the event that there exists a double- path contained in $\mathsf{Strip}_m$, from $\mathsf {Mid}_0(\rho _\mathrm {in}m)$ to $\mathsf {Mid}_m(\rho _\mathrm {in}m)$ that is not contained in $\mathsf{Rect}(\rho _\mathrm {out}m , m)$.

Then, for any $C_{\mathsf {wig}} >0$ there exists $\Delta _{\mathsf {wig}} >0$ such that, for any $m \le n$,4.9

In other words, the lemma tells us that, if vertical crossings of $\mathsf{Strip}_m$ starting on $\mathsf {Mid}_0(\rho _\mathrm {in}m)$ and ending on $\mathsf {Mid}_h(\rho _\mathrm {in}m)$ wiggle either too little (when ${\mathscr {H}}_\mathsf {wig}^\mathrm {in}(m)$ occurs) or too much (when ${\mathscr {H}}_\mathsf {wig}^\mathrm {out}(m)$ occurs), then wide rectangles may be crossed horizontally.

##### Proof

Take $\rho _\mathrm {in}= 1/72$ and $\rho _\mathrm {out}= 4 + \rho _\mathrm {in}$. Fix some constant $C_\mathsf {wig}$; the value of $\Delta _{\mathsf {wig}}$ will be determined below. If $\mu [{\mathscr {H}}_\mathsf {wig}^\mathrm {in}(m) \cup {\mathscr {H}}_\mathsf {wig}^\mathrm {out}(m) ] > C_{\mathsf {wig}}$, then either $\mu [{\mathscr {H}}_\mathsf {wig}^\mathrm {in}(m) ] > C_{\mathsf {wig}}/2$ or $\mu [{\mathscr {H}}_\mathsf {wig}^\mathrm {out}(m)] > C_{\mathsf {wig}}/2$.

Suppose the second inequality is valid. If the event ${\mathscr {H}}_\mathsf {wig}^\mathrm {out}$ occurs, one of the rectangles $[\rho _\mathrm {in},\rho _\mathrm {out}m]\times [0,m]$ or $[-\rho _\mathrm {out}m,-\rho _\mathrm {in}]\times [0,m]$ is crossed horizontally by a double- path. Thus, due to the invariance of $\mu$ under horizontal shift, . The implication is therefore proved in this case for any $\Delta _\mathsf {wig}\le C_{\mathsf {wig}}/4$.

It remains to consider the case when $\mu [{\mathscr {H}}_\mathsf {wig}^\mathrm {in}(m) ] > C_{\mathsf {wig}}/2$. Then, by symmetry, the probability that there exists a double- path from $\mathsf {Mid}_{0}(\rho _\mathrm {in}m)$ to $\mathsf {Mid}_{h}(\rho _\mathrm {in}m)$ contained in $\mathsf{Strip}_m$ and to the right of $\mathsf {L_v}(-\rho _\mathrm {in}m)$ is greater than $C_{\mathsf {wig}}/4$. Moreover, due to the invariance of $\mu$ under horizontal translation and symmetry, with probability at least $C_\mathsf {wig}/ 4$, the segment $(2\rho _\mathrm {in}m,0 )+ \mathsf {Mid}_{0}(\rho _\mathrm {in}m)$ may be connected to $(2\rho _\mathrm {in}m,0 ) + \mathsf {Mid}_{h}(\rho _\mathrm {in}m)$ using a double- path contained in $\mathsf{Strip}_m$ that stays to the left of $\mathsf {L_v}(3\rho _\mathrm {in}m)$. The two events described above are increasing, and due to the FKG inequality, they occur simultaneously with probability at least $(C_\mathsf {wig}/ 4)^2$. When both do occur, then the rectangle $[-\rho _\mathrm {in}m,3\rho _\mathrm {in}m] \times [0,m]$ is crossed vertically by a path of double-. See Figure 11 - left diagram.

Let ${\mathscr {A}}$ be the event that both rectangles $[-6\rho _\mathrm {in},-2\rho _\mathrm {in}m]\times [0,m]$ and $[2\rho _\mathrm {in},6\rho _\mathrm {in}m]\times [0,m]$ contain double- vertical crossings. Due to the FKG inequality, invariance under horizontal translation, and the above estimate, we find that $\mu ({\mathscr {A}}) \ge (C_{\mathsf {wig}}/4)^4$.

A rotated version of Lemma 4.5 (and Corollary 4.6) applies to the rectangle $\mathsf{Rect}_{6\rho _\mathrm {in}m,m}$, and proves that the left-most vertical crossing of $[-6\rho _\mathrm {in},-2\rho _\mathrm {in}m]\times [0,m]$ connects via a double- path to the right-most vertical crossing of $[2\rho _\mathrm {in},6\rho _\mathrm {in}m]\times [0,m]$ with probability at least 1/36. (The choice of $\rho _\mathrm {in}$ ensures that this rectangle is sufficiently thin to be covered by three disjoint translates of $\mathsf{Par}_{m/3,m/3}$ placed one on top of the other. Slight adaptations to the proof need to be made; we leave this to the reader. See also Figure 11 - right diagram.) Using this and the lower bound on the probability of ${\mathscr {A}}$, we findThe above is akin to (4.8). We conclude as in the proof of Lemma 4.8 that4.10for some sufficiently small constant $\Delta _{\mathsf {wig}}>0$ depending only on $C_{\mathsf {wig}}$ and $\rho _\mathrm {in}$. $\quad \square$

The last two lemmas will be used for different scales $m \le n$. The following will only be used at scale *n*. To simplify notation, we only state it at this scale.

##### Lemma 4.10

(Vertical paths have fixed width). Let $c_\mathsf {loc}= 5$ and fix any $\epsilon \le \rho _\mathrm {in}$. For $k \ge 1$, let ${\mathscr {G}}_{\mathsf {loc}}(\epsilon , k)$ be the event that any double- path contained in $\mathsf{Strip}_n$ with endpoints in $\mathsf {Mid}_0(\epsilon n)$ and $\mathsf {Mid}_n(\epsilon n)$ intersects the vertical line $\mathsf {L_v}(k\epsilon n)$ but not $\mathsf {L_v}[(k+c_\mathsf {loc})\epsilon n]$. Then, for any constant $C_{\mathsf {loc}} > 0$ there exists $\Delta _\mathsf {loc}=\Delta _\mathsf {loc}(\epsilon ,C_\mathsf {loc}) > 0$ such that

Notice that ${\mathscr {G}}_{\mathsf {loc}}(\epsilon , k)$ contains all configurations with no double- path connecting $\mathsf {Mid}_0(\epsilon n)$ to $\mathsf {Mid}_n(\epsilon n)$ inside $\mathsf{Strip}_n$. Indeed, the condition is trivially satisfied.

##### Proof

Fix $\epsilon \le \rho _\mathrm {in}$ and $C_{\mathsf {loc}} > 0$; to simplify notation we will consider  $\rho _\mathrm {in}/\epsilon$ to be an integer. Assume that $\mu [ {\mathscr {G}}_{\mathsf {loc}}(\epsilon , k) ] < 1 - C_\mathsf {loc}$ for all $k \ge {\rho _\mathrm {in}}/{\epsilon }$.

For $k \ge 1$, let ${\mathscr {E}}_k$ be the event that there exists a double- path in $\mathsf{Strip}_n$ with endpoints in $\mathsf {Mid}_0(\epsilon n)$ and $\mathsf {Mid}_n(\epsilon n)$ and which intersects $\mathsf {L_v}(k\epsilon n)$. Also write ${\mathscr {E}}_0$ for the event that $\mathsf {Mid}_0(\epsilon n)$ and $\mathsf {Mid}_n(\epsilon n)$ are connected by a double- path contained in $\mathsf{Strip}_n$, with no other restriction. The events ${\mathscr {E}}_k$ are increasing each, but form a decreasing sequence. Additionally, define ${\mathscr {A}}_k$ as the event that there exists a double- path in $\mathsf{Strip}_n$ that connects $\mathsf {Mid}_0(\epsilon n)$ to $\mathsf {Mid}_n(\epsilon n)$ and which does not intersect $\mathsf {L_v}(k\epsilon n)$. Then each ${\mathscr {A}}_k$ is an increasing event and the sequence ${\mathscr {A}}_k$ is increasing in *k*. Moreover ${\mathscr {G}}_{\mathsf {loc}}(\epsilon , k) = ({\mathscr {A}}_k \cup {\mathscr {E}}_{k+5})^c$.

Notice that ${\mathscr {E}}_0^c$ is contained in all events  ${\mathscr {G}}_{\mathsf {loc}}(\epsilon , k)$, hence $\mu ({\mathscr {E}}_0) \ge C_\mathsf {loc}$. Set *k* to be the smallest index such that $\mu ({\mathscr {A}}_k) \ge C_\mathsf {loc}/2$. The existence of *k* is guaranteed by the fact that $\lim _j \mu ({\mathscr {A}}_j) = \mu ({\mathscr {E}}_0) \ge C_\mathsf {loc}$.

Suppose first that $k \le {\rho _\mathrm {in}}/{\epsilon }$. Then, ${\mathscr {H}}_\mathsf {wig}^\mathrm {in}(n) \ge \mu ({\mathscr {A}}_k) \ge C_\mathsf {loc}/2$, and Lemma 4.9 shows that  is bounded below by some constant that only depends on $C_\mathsf {loc}$.

Henceforth we assume that  $k > {\rho _\mathrm {in}}/{\epsilon }$. Then, due to our initial assumption,

$$\begin{aligned} 1 - C_\mathsf {loc}> \mu ({\mathscr {G}}_{\mathsf {loc}}(\epsilon , k-1)) \ge 1 - \mu ({\mathscr {A}}_{k-1}) -\mu ({\mathscr {E}}_{k+4}) > 1 - C_\mathsf {loc}/2 -\mu ({\mathscr {E}}_{k+4}), \end{aligned}$$

which implies $\mu ({\mathscr {E}}_{k+4}) > C_\mathsf {loc}/2$. Write ${{\tilde{{\mathscr {A}}}}}_k$ for the horizontal shift of the event ${\mathscr {A}}_k$ by $4\epsilon n$. By the choice of *k*, we have $\mu ({{\tilde{{\mathscr {A}}}}}_k ) = \mu ({\mathscr {A}}_k )\ge C_\mathsf {loc}/2$. Using the FKG inequality, we find

$$\begin{aligned} \mu ({{\tilde{{\mathscr {A}}}}}_k \cap {\mathscr {E}}_{k+4})\ge \mu ({\tilde{{\mathscr {A}}}}_k)\,\mu ({\mathscr {E}}_{k+4}) \ge (C_\mathsf {loc}/2)^2. \end{aligned}$$

Notice now that, if both ${{\tilde{{\mathscr {A}}}}}_k$ and ${\mathscr {E}}_{k+4}$ occur, then the paths in the definition of these two events necessarily intersect. In conclusionAs in the proof of Lemma 4.8, this implies that  is larger than some threshold depending only on $\epsilon$ and $C_\mathsf {loc}$, and the lemma is proved. $\quad \square$

In the proof of Proposition 4.7 we will work with two scales: the scale *n* and a lower scale *m* chosen below. Moreover, the endpoints of the vertical paths will be fixed in some segment of length $2\epsilon n$ where $\epsilon > 0$ is also chosen below.

Fix $m = \frac{\rho _\mathrm {in}}{2\rho _\mathrm {out}} n$. Then, fix some $\epsilon > 0$ so that

4.11 $$\begin{aligned} \epsilon \, n<\tfrac{1}{2} \rho _\mathrm {in}\, m \qquad \text { and } \qquad \rho _\mathrm {out}\, m < \rho _\mathrm {in}\, n - c_\mathsf {loc}\, \epsilon \, n. \end{aligned}$$

In conclusion, the scales $\epsilon n$, *m* and *n* are fixed so that $\epsilon n$ is much smaller than *m*, which in turn is much smaller than *n*. All constants below depend on the ratios between these scales.

Write $\Gamma _L$ and $\Gamma _R$ for the left- and right-most, respectively, double- paths contained in $\mathsf{Strip}_n$, with lower endpoint on $\mathsf {Mid}_0(\epsilon n)$ and top endpoint in $\mathsf {Mid}_n(\epsilon n)$ (recall that these are paths formed of edges of the hexagonal lattice). If no such crossings exists, set $\Gamma _L = \Gamma _R = \emptyset$. We will always orient such paths from their endpoint on $\mathsf {L_h}(0)$ towards that on $\mathsf {L_h}(n)$.

When $\Gamma _L$ and $\Gamma _R$ exist and are disjoint, write $\textsf {Int}(\Gamma _L, \Gamma _R)$ for the domain with boundary formed of the concatenation of $\Gamma _R$, the segment of $\mathsf {L_h}(n)$ between the top endpoints of $\Gamma _R$ and $\Gamma _L$ (from right to left), $\Gamma _L$ (in reverse), and the segment of $\mathsf {L_h}(0)$ between the bottom endpoints of $\Gamma _L$ and $\Gamma _R$ (from left to right). Also let $\mathsf {Ext}(\Gamma _L,\Gamma _R)$ be the set of faces of $\mathsf{Strip}_n$ which are not strictly inside $\textsf {Int}(\Gamma _L, \Gamma _R)$; precisely, $\mathsf {Ext}(\Gamma _L,\Gamma _R)$ contains all faces of $\mathsf{Strip}_n\setminus \textsf {Int}(\Gamma _L, \Gamma _R)$ as well as all faces adjacent to $\Gamma _L$ or $\Gamma _R$.

It is standard that $\Gamma _L$ and $\Gamma _R$ may be explored from their left and right, respectively. That is, for $\gamma _L$ and $\gamma _R$ two possible realisations of $\Gamma _L$ and $\Gamma _R$, respectively, the event $\{\Gamma _L = \gamma _L \text { and } \Gamma _R =\gamma _R\}$ is measurable with respect to the state of faces in  $\mathsf {Ext}(\gamma _L,\gamma _R)$.

Our next goal is to show that, whenever $\Gamma _L$ and $\Gamma _R$ exist and behave reasonably well, they have a positive probability to be connected inside $\textsf {Int}(\Gamma _L, \Gamma _R)$ by a path of double-. The notion of well-behaved vertical paths is defined below.

For an edge-path $\Gamma$ contained in $\mathsf{Strip}_n$, with starting point in $\mathsf {Mid}_0(\epsilon n)$ and endpoint in $\mathsf {Mid}_n(\epsilon n)$, let $\Gamma ^{b}$ be the segment of $\Gamma$ contained between its starting-point and its first visit of $\mathsf {L_h}(m)$. Let $\Gamma ^t$ be the segment of $\Gamma$ between its last visit of $\mathsf {L_h}(n-m)$ and its endpoint.

Define ${\mathscr {G}}_\mathsf {wb}(k)$ as the event that any double- path $\Gamma$ contained in $\mathsf{Strip}_n$, with starting point in $\mathsf {Mid}_0(\epsilon n)$ and endpoint in $\mathsf {Mid}_n(\epsilon n)$ is well-behaved (for this value of *k*), that is

(i) $\Gamma ^b$ has one endpoint in $\mathsf {Mid}_{m}(2\epsilon n)$ and $\Gamma ^t$ has one endpoint in $\mathsf {Mid}_{n-m}(2\epsilon n)$;
(ii) $\Gamma ^b$ and $\Gamma ^t$ are both contained in $\mathsf{Rect}(\rho _\mathrm {out}m,n)$ but each crosses $\mathsf {L_v}(\rho _\mathrm {in}m)$,
(iii) $\Gamma$ crosses $\mathsf {L_v}(k\,\epsilon \, n)$ but not $\mathsf {L_v}((k+c_\mathsf {loc})\,\epsilon \, n)$.

In order to apply our reasoning, we will ask that $\Gamma _L$ and $\Gamma _R$ are well-behaved, that is, we will ask that ${\mathscr {G}}_\mathsf {wb}(k)$ occurs for some *k*. This is guaranteed by the following result.

##### Lemma 4.11

(Paths are well-behaved). For any $C_\mathsf {wb}> 0$, there exists a constant $\Delta _{\mathsf {wb}} > 0$ such that

##### Proof

Fix $C_\mathsf {wb}> 0$ and assume $\mu [ {\mathscr {G}}_\mathsf {wb}(k) ] < 1 - C_\mathsf {wb}$ for all $k\ge {\rho _\mathrm {in}}/{\epsilon }$. Then, one out of the three conditions defining ${\mathscr {G}}_\mathsf {wb}(k)$ fails with probability at least $C_\mathsf {wb}/ 3$ for every *k*. Thus, at least one of the following cases occurs:

- (i) fails with probability at least  $C_\mathsf {wb}/ 3$ for some *k*. Then Lemma 4.8 states that  for some $\Delta _{\mathsf {mid}} > 0$ depending only on $C_\mathsf {wb}$. Using the horizontal translation invariance of $\mu$, the same bound applies to any horizontal translate $\mathsf{Rect}_{2m,m} + j(m,0)$ of $\mathsf{Rect}_{2m,m}$, with $j \in {\mathbb {Z}}$. Using this and Corollary 4.6, we find 4.12 for some universal constant $c> 0$.
- (ii) fails with probability at least  $C_\mathsf {wb}/ 3$ for some *k*. Then Lemma 4.9 implies that  for some $\Delta _{\mathsf {wig}} > 0$ depending only on $C_\mathsf {wb}$. As above, we conclude that  .
- (iii) fails with probability at least  $C_\mathsf {wb}/ 3$ for all *k*. Then, by Lemma 4.10 applied with $C_\mathsf {loc}= C_\mathsf {wb}/ 3$, we deduce .

We conclude that in all cases,  is bounded below by a constant depending only on $C_\mathsf {wb}$, as required. $\quad \square$

Now that we proved that some ${\mathscr {G}}_\mathsf {wb}(k)$ occurs with high probability, we will show that, when it does occur, $\Gamma _R$ and $\Gamma _L$ connect to each other. Since $\Gamma _R$ and $\Gamma _L$ are measurable in terms of the spins in $\mathsf {Ext}(\Gamma _L,\Gamma _R)$, the same applies to  ${\mathscr {G}}_\mathsf {wb}(k)$. Indeed, if $\Gamma _L$ and $\Gamma _R$ satisfy the conditions of ${\mathscr {G}}_\mathsf {wb}(k)$, then so do all double- paths contained in $\mathsf{Strip}_n$, from $\mathsf {Mid}_0(\epsilon n)$ to $\mathsf {Mid}_n(\epsilon n)$.

##### Lemma 4.12

There exists some universal constant $C_{\mathsf {cnt}} > 0$ such that, for any possible realisations $\gamma _L,\gamma _R$ of $\Gamma _L,\Gamma _R$ with the property that ${\mathscr {G}}_\mathsf {wb}(k)$ occurs for some $k \ge {\rho _\mathrm {in}}/{ \epsilon }$ and any red spin configuration $\zeta$ such that $\Gamma _L = \gamma _L$ and $\Gamma _R = \gamma _R$4.134.14

The conditioning in (4.13) may be reduced simply to {$\sigma _r = \zeta$ on $\mathsf {Ext}(\gamma _L,\gamma _R)$}, since this determines $\Gamma _L = \gamma _L, \, \Gamma _R = \gamma _R$, which in turn implies that ${\mathscr {G}}_\mathsf {wb}(k)$ occurs. We included the latter conditions in (4.13) to emphasise their importance.

The lemma above is the heart of the proof of Theorem 4.7.

##### Proof

Fix $\gamma _L$, $\gamma _R$ and $\zeta$ as in the statement. Let $k \ge {\rho _\mathrm {in}}/{ \epsilon }$ be some value for which ${\mathscr {G}}_\mathsf {wb}(k)$ occurs. We may assume that $\gamma _L$ and $\gamma _R$ are disjoint, otherwise the conclusion is trivially attained. We will proceed in two steps, first we will create simple- connections between $\gamma _L$ and $\gamma _R$, close to the top and bottom of $\mathsf{Strip}_n$, respectively. In a second stage, we connect $\gamma _L$ and $\gamma _R$ by a double- path contained between the two simple- paths shown to exist in the previous step.

Recall that $\gamma _L$ and $\gamma _R$ are oriented from bottom to top. Let $R_1 = \mathsf{Rect}(\rho _\mathrm {out}m, m)$ and $R_2 = R_1 + (0,n-m)$ be the vertical translation of $R_1$ contained between $\mathsf {L_h}(n-m)$ and $\mathsf {L_h}(n)$. Due to ${\mathscr {G}}_\mathsf {wb}(k)$ occuring, $\gamma _L^b$ and $\gamma _R^b$ are contained in $R_1$, while $\gamma _L^t$ and $\gamma _R^t$ are contained in $R_2$.

**Fig. 12:**
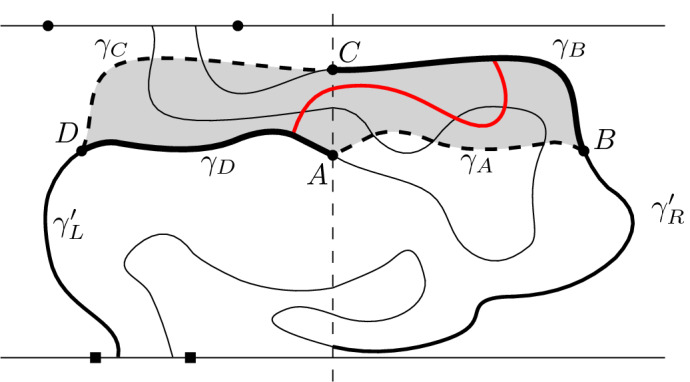
The paths $\gamma _L^b$ and $\gamma _R^b$ are drawn in solid lines; the thicker parts are $\gamma _L'$ and $\gamma _R'$. The reflections of parts of $\gamma _L'$ and $\gamma _R'$ are in dashed lines. The domain ${\mathscr {D}}$ is shaded. Observe that any crossing from $\gamma _B$ to $\gamma _D$ in ${\mathscr {D}}$ induces a connection between $\gamma _L^b$ and $\gamma _R^b$

**Step 1: Simple-**  **crossings.** Let ${\mathscr {I}}$ be the event that $\gamma _L$ and $\gamma _R$ are connected by two simple- paths contained in $R_1$ and $R_2$, respectively. We will now prove that ${\mathscr {I}}$ has positive probability, uniformly in *m*, *n*, $\gamma _L$, $\gamma _R$ and $\zeta$. We do this for the connection in $R_1$; the same argument applies in $R_2$. The argument used in this step is exactly that of [18]. Figure 12 contains an illustration of the construction below.

Recall that both $\gamma _L^b$ and $\gamma _R^b$ intersect $\mathsf {L_v}(\rho _\mathrm {in}m)$ but that their endpoints are in $\mathsf {Mid}_{0}(\epsilon n)$ and $\mathsf {Mid}_{m}(2\epsilon n)$, hence to the left of $\mathsf {L_v}(\rho _\mathrm {in}m)$. Let *A* be the first point where $\gamma _L$ intersects $\mathsf {L_v}(\rho _\mathrm {in}m)$ and write $\gamma _L'$ for the subpath of $\gamma _L$ from its starting point up to *A*.

Then $\gamma _R^b$ contains at least one subpath contained in the part of $R_1$ to the right of $\mathsf {L_v}(\rho _\mathrm {in}m)$, which has both endpoints on $\mathsf {L_v}(\rho _\mathrm {in}m)$, one below *A* and one above *A* (this is because $\gamma _R^b$ has both its endpoints to the left of $\mathsf {L_v}(\rho _\mathrm {in}m)$). Write $\gamma _R'$ for the left-most such path and let *C* be the endpoint of $\gamma _R'$ above *A*.

Write $\tau$ for the reflection with respect to $\mathsf {L_v}(\rho _\mathrm {in}m)$ (actually, with respect to the vertical axis $\{\lfloor \rho _\mathrm {in}m \rfloor \}\times {\mathbb {R}}$). Then $\tau$ leaves the lattice invariant.

Observe now that $\tau (\gamma _L')$ intersects $\gamma _R'$. Indeed, $\tau (\gamma _L')$ runs from *A* to $\mathsf {L_h}(0)$ and is contained in the region to the right of $\mathsf {L_v}(\rho _\mathrm {in}m)$, while $\gamma _R'$ separates *A* from $\mathsf {L_h}(0)$ in this same region. Let *B* be the first intersection point of $\tau (\gamma _L')$ with $\gamma _R'$ when starting from *A* and let $\gamma _A$ be the subpath of $\tau (\gamma _L')$ between *A* and *B*. Let $\gamma _B$ be the subpath of $\gamma _R'$ between *B* and *C*. Finally set $\gamma _C = \tau (\gamma _B)$, $\gamma _D =\tau (\gamma _A)$ and $D = \tau (B)$.

The paths $\gamma _A,\gamma _B,\gamma _C$ and $\gamma _D$ only intersect at their endpoints and their concatenation bounds a domain which we call ${\mathscr {D}}$.

Let us derive a bound on the crossing probability of ${\mathscr {D}}$, independently of how ${\mathscr {D}}$ was formed. Consider the red spin configuration $\xi$ on $\mathsf{Cyl}$ consisting only of  with the exception of the faces adjacent to $\gamma _B$ and those adjacent to $\gamma _D$, which have spin . By the same reasoning as in Lemma 3.5 and due to the invariance of ${\mathscr {D}}$ under $\tau$, we obtainWrite $\mu _{\mathscr {D}}^\xi$ for the conditional measure above.

By Corollary 2.12 and due to the condition $\Gamma _L = \gamma _L$ and $\Gamma _R = \gamma _R$, the measure $\mu [.\,|\,\sigma _r = \zeta \text { on }\mathsf {Ext}(\gamma _L,\gamma _R)]$ restricted to ${\mathscr {D}}\cap \textsf {Int}(\gamma _L, \gamma _R)$ dominates the restriction of $\mu _{\mathscr {D}}^\xi$ to this same set of faces.

Set ${\mathscr {A}}$ to be the event that there exists a face-path $\chi$ in ${\mathscr {D}}$, with the first and last faces adjacent to $\gamma _B$ and $\gamma _D$, respectively, and such that all faces of $\chi$ that are contained in $\textsf {Int}(\gamma _L, \gamma _R)$ have spin . ThenNow observe that a path $\chi$ as in the definition of ${\mathscr {A}}$ necessarily contains a subpath contained in $\textsf {Int}(\gamma _L, \gamma _R)$ with the first and last faces adjacent to $\gamma _L$ and $\gamma _R$, respectively. We conclude thatUsing the same argument in $R_2$ and the FKG inequality, we obtain

4.15 $$\begin{aligned} \mu \big [{\mathscr {I}}\, \big |\, \sigma _r = \zeta \text { on } \mathsf {Ext}(\gamma _L,\gamma _R)\big ] \ge \tfrac{1}{9}. \end{aligned}$$

**Fig. 13:**
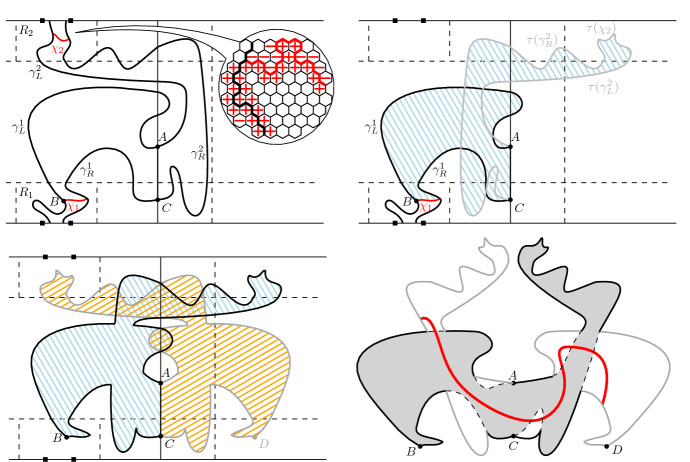
Top left: the paths $\gamma _L$ and $\gamma _R$ are connected in $R_1$ and $R_2$ by simple- paths (in red). The dashed lines delimit $\mathsf{Strip}_m$ and $(0,n-m) + \mathsf{Strip}_m$; $\mathsf {L_v}((k+c_\mathsf {loc})\,\epsilon \, n)$ is also dashed. Top right: The domain ${\mathscr {D}}^1$ contains $\chi ^1$ and $\tau (\chi ^2)$ in its boundary. Bottom left: the domain ${\mathscr {D}}$ is formed from ${\mathscr {D}}^1$ (blue) and ${\mathscr {D}}^2$ (orange). These two domains are in different copies of ${\mathbb {H}}$, glued along the segment [*A*, *C*]. Bottom right: a deformation of ${\mathscr {D}}$ allows to embed it in the plane; it contains $\textsf {Int}$, whose deformation is shaded. Any crossing from (*AB*) to (*CD*) contains a path from $\gamma _L$ to $\gamma _R$ in $\textsf {Int}$

**Step 2: Double-** **crossing.** We will now prove that4.16The procedure is similar to that of Step 1, but at scale *n* rather than *m* and with some additional difficulties. We recommend that the reader inspects Figure 13, which contains the strategy of the proof as well as the relevant notation.

When ${\mathscr {I}}$ occurs, we will denote by $\Xi ^1$ and $\Xi ^2$ be the lowest and highest, respectively, paths of simple- from $\gamma _L$ to $\gamma _R$, contained in $\textsf {Int}(\gamma _L, \gamma _R)$. More precisely, define $\Xi ^1$ to be the lowest edge-path contained in $\textsf {Int}(\gamma _L, \gamma _R)$, with endpoints on $\gamma _L$ and $\gamma _R$, respectively, with the property that all faces above it have spin . Define $\Xi ^2$ similarly, only that it is highest and that all faces below it are required to have spin . By the definition of ${\mathscr {I}}$ and the first condition of ${\mathscr {G}}(k)$, $\Xi ^1$ and $\Xi ^2$ are contained in $R_1$ and $R_2$, respectively, whenever ${\mathscr {I}}$ occurs.

Let $\chi ^1$ and $\chi ^2$ be possible realisations of $\Xi ^1$ and $\Xi ^2$, respectively, such that ${\mathscr {I}}$ occurs. Define the domain $\textsf {Int}= \textsf {Int}(\gamma _L, \gamma _R, \chi ^1, \chi ^2)$ as the set of faces delimited by these four paths. Also let $\mathsf {Ext}=\mathsf {Ext}(\gamma _L, \gamma _R, \chi ^1, \chi ^2)$ be the set of faces outside $\textsf {Int}$ along with those of $\partial _\mathrm {in}\textsf {Int}$. By a standard exploration argument, the event $\{\Xi ^1 = \chi ^1, \Xi ^2 = \chi ^2\}$ is measurable with respect to the spins on $\mathsf {Ext}$. Fix a red spin configuration $\xi$ on $\mathsf {Ext}(\gamma _L, \gamma _R, \chi ^1, \chi ^2)$ with $\xi = \zeta$ on $\mathsf {Ext}(\gamma _L,\gamma _R)$ and such that $\Xi ^1 = \chi ^1$, $\Xi ^2 = \chi ^2$. This implies in particular that all faces of $\partial _\mathrm {in}\textsf {Int}$ have spin  in $\xi$.

The line $\mathsf {L_v}(k\,\epsilon \, n)$ separates $\chi ^1$ from $\chi ^2$ inside the simply connected domain $\textsf {Int}$. It follows that there exists at least one segment of $\mathsf {L_v}(k\,\epsilon \, n)$ that is fully contained in $\textsf {Int}$ and that separates $\chi ^1$ from $\chi ^2$ inside this domain. Indeed, $\mathsf {L_v}(k\,\epsilon \, n)$ needs to intersect both $\gamma _L$ to $\gamma _R$ in order to separate $\chi ^1$ from $\chi ^2$. Consider the intersections of $\mathsf {L_v}(k\,\epsilon \, n)$ with $\gamma _L$ and $\gamma _R$ in increasing vertical order; there necessarily exists one intersection with $\gamma _R$ followed by one with $\gamma _L$. The segment of $\mathsf {L_v}(k\,\epsilon \, n)$ between these two intersections has the desired property.

Let [*A*, *C*] be the first such segment when going from $\chi ^1$ to $\chi ^2$, where *A* denotes its higher endpoint (the segments with this property are naturally ordered, for instance by their end-points on $\Gamma _L$). Then *A* is a point of $\gamma _L$ while *C* is a point of $\gamma _R$. Write $\gamma ^1_L$ and $\gamma ^2_L$ for the subpaths of $\gamma _L$ from the intersection with $\chi ^1$ to *A* and from *A* to the intersection with $\chi ^2$, respectively. The same notation applies to $\gamma _R$.

Then [*A*, *C*] separates $\textsf {Int}$ into two sub-domains. The first, which we call $\textsf {Int}^1$, has boundary formed of $\chi ^1$, $\gamma _L^1$, [*A*, *C*] and $\gamma _R^1$. The boundary of the second, called $\textsf {Int}^2$, is the concatenation of $\chi ^2$, $\gamma _R^2$, [*A*, *C*] and $\gamma _L^2$.

Let $\tau$ be the reflection with respect to the vertical axis $\mathsf {L_v}(k\,\epsilon \, n)$. Now define ${\mathscr {D}}^1$ as the union of the sets of faces of $\textsf {Int}^1$ and $\tau (\textsf {Int}^2)$. Then ${\mathscr {D}}^1$ is itself a domain, whose boundary consists of $\chi ^1$, $\tau (\chi ^2)$, [*A*, *C*] and pieces of $\gamma _L^1$, $\gamma _R^1$, $\tau (\gamma _L^2)$ and $\tau (\gamma _R^2)$. It is particularly important that $\chi ^1$ is fully part of the boundary of ${\mathscr {D}}^1$. This is because $\tau (\textsf {Int}^2)$ lies entirely to the right of $\mathsf {L_v}((k-c_\mathsf {loc})\,\epsilon \, n)$, and thus does not intersect $R_1$ (property (*iii*) of well-behaved paths, see also (4.11)). For similar reasons, $\tau (\chi ^2)$ is also fully contained in the boundary of ${\mathscr {D}}^1$.

Let *B* be the intersection point of $\gamma _L$ and $\chi ^1$; it is on the boundary of ${\mathscr {D}}^1$. Write ${\mathscr {D}}^2 = \tau ({\mathscr {D}}^1)$ and $D = \tau (B)$. Define the domain ${\mathscr {D}}$ by gluing ${\mathscr {D}}^1$ and ${\mathscr {D}}^2$ along the segment [*A*, *C*]. The result of this operation is not a domain of ${\mathbb {H}}$. Indeed, ${\mathscr {D}}^1$ and ${\mathscr {D}}^2$ may intersect in ${\mathbb {H}}$; we will consider them as embedded in two different copies of ${\mathbb {H}}$ that are then glued along the segment [*A*, *C*]. Nevertheless, ${\mathscr {D}}$ is planar (that is, it may be embedded in the plane after some distortion; see Figure 13) and is simply connected. Orient $\partial _E {\mathscr {D}}$ in counter-clockwise order and write (*AB*), (*BC*) etc. for the portions of $\partial _E {\mathscr {D}}$ between *A* and *B*, *B* and *C* etc.

Let us study the measure with boundary conditions  on ${\mathscr {D}}$. By the same argument as for Lemma 3.6, either (*AB*) is connected to (*CD*) inside ${\mathscr {D}}$ by a path of double- or double-, or (*BC*) is connected to (*DA*) by a path of double- or double-. As in Lemma 3.8, the domain is symmetric with respect to $\tau$ and the boundary conditions  favour the connection with double-. Thus we find4.17Observe now that ${\mathscr {D}}$ contains $\textsf {Int}$. Moreover, any path crossing from  (*AB*) to (*CD*) in ${\mathscr {D}}$ contains a subpath which is contained in $\textsf {Int}$ and which has endpoints on $\gamma _L$ and $\gamma _R$, respectively. Indeed, the segment (*AB*) is above $\gamma _L^1$, while (*CD*) is below $\gamma ^2_R$.

Finally, we claim that the restriction of $\mu (.\, |\, \sigma _r = \xi \text { on }\mathsf {Ext})$ to $\textsf {Int}$ dominates that of . We start off with a heuristic explanation. The key to this argument is to observe that ${\mathscr {D}}$ may be obtained from $\textsf {Int}$ by “pushing away” parts of the boundary of ${\mathscr {D}}$, but that these only belong to $\gamma _L$ and $\gamma _R$, not to $\chi ^1$ or $\chi ^2$. Since these are double- paths in $\xi$, the monotonicity of boundary conditions applies, and we may conclude.

Let us now present a rigorous proof of this domination with a slightly weaker conclusion. As already explained, ${\mathscr {D}}$ is part of two copies of ${\mathbb {H}}$ glued along the segment [*A*, *C*]. Let ${\mathscr {D}}'$ be a planar domain of this graph that contains ${\mathscr {D}}$ along with all faces adjacent to it. Then, due to the Spatial Markov property that also applies in this slightly different setting,  is the restriction to ${\mathscr {D}}$ of .

Since ${\mathscr {D}}'$ is planar, the FKG inequality holds for $\mu _{{\mathscr {D}}'}$. Let ${\mathscr {A}}^-$ be the set of faces of $\partial _\mathrm {out}\textsf {Int}$ adjacent to $\chi ^1$ or $\chi ^2$. Let ${\mathscr {A}}^+$ be all the other faces of ${\mathscr {D}}' \setminus \textsf {Int}$ along with $\partial _\mathrm {in}\textsf {Int}$. Then, by the monotonicity of boundary conditions (Corollary 2.12*(i)*) and the consideration above, the restriction of   to $\textsf {Int}$ is dominated by that of . Moreover, the latter is equal to the restriction of  to $\textsf {Int}$. (Here ${\mathscr {A}}^-$ is also viewed as a subset of $\mathsf{Cyl}$.)

In conclusion**Conclusion.** Equations (4.15) and (4.16) imply thatwhich is the desired bound. $\quad \square$

We are finally ready to prove the main result of the section, namely Proposition 4.7.

##### Proof of Proposition 4.7

Recall from (4.11) that *m* and $\epsilon$ are fixed. Let . The bottom boundary of $\mathsf{Rect}_{3n,n}$ may be partitioned into $18/\epsilon$ segments of length $\epsilon n /3$. At least one of these segments is connected inside $\mathsf{Strip}_n$ by a double- path to  $\mathsf {L_v}(n)$ with probability at least $C_v \epsilon /18$. Since the measure $\mu$ is translation invariant,4.18Let $\Delta _\mathsf {mid}$ be given by Lemma 4.8 with $C_{\mathsf {mid}} = \frac{C_v \epsilon }{36}$ and $\epsilon /6$ instead of $\epsilon$. If  the proof is complete. We will therefore assume that $\mu [{\mathscr {H}}_\mathsf {mid}(m)] < C_{\mathsf {mid}}$, which along with (4.18) implies4.19For $j \in {\mathbb {Z}}$, write $M_j^{b} = (j \frac{2}{3}\epsilon n, 0) + \mathsf {Mid}_0(\epsilon n/3)$ and $M_j^{t} = (j \frac{2}{3}\epsilon n, 0) + \mathsf {Mid}_n(\epsilon n/3)$. Let ${\mathscr {J}}$ be the event that $M_j^{b}$ is connected to $M_j^{t}$ by a double- path inside $\mathsf{Strip}_n$ for both $j = -1$ and $j = 1$. Using again the translation invariance of $\mu$, the FKG inequality and (4.19), we find

4.20 $$\begin{aligned} \mu [{\mathscr {J}}] \ge \Big (\tfrac{C_v \epsilon }{36}\Big )^2. \end{aligned}$$

Now let $\Delta _\mathsf {wb}$ be the constant given by Lemma 4.11 with $C_\mathsf {wb}= \frac{1}{2} \big (\tfrac{C_v \epsilon }{36}\big )^2$. If  we have obtained the result. We may therefore assume the opposite, thus that

$$\begin{aligned} \mu [ {\mathscr {G}}_\mathsf {wb}(k) ] > 1 - C_\mathsf {wb}, \end{aligned}$$

for some $k \ge {\rho _\mathrm {in}}/{\epsilon }$. By choice of $C_\mathsf {wb}$ and using a union bound, we conclude that

4.21 $$\begin{aligned} \mu [{\mathscr {J}}\cap {\mathscr {G}}_\mathsf {wb}(k) ] \ge \tfrac{1}{2} \Big (\tfrac{C_v \epsilon }{72}\Big )^2 = C_\mathsf {wb}. \end{aligned}$$

Applying now Lemma 4.12, we findWhen ${\mathscr {J}}$ occurs, the endpoints of $\Gamma _L$ are contained in $M_{-1}^b$ and $M_{-1}^t$, respectively, while those of $\Gamma _R$ are in $M_{1}^b$ and $M_{1}^t$. Thus, when all three events above occur simultaneously, $M_{-1}^b$ and $M_{1}^b$ are connected inside $\mathsf{Strip}_n$ by a path of double-. We conclude thatWe conclude in the same way as in the proof of Lemma 4.8: the lower bound above applies also to  for all $-\tfrac{3}{2 \epsilon } \le j \le \tfrac{3}{2 \epsilon }$. Using the FKG inequality, the intersection of all these translations occurs with probability at least $(C_\mathsf {cnt}\cdot C_\mathsf {wb})^{3/\epsilon +1}$. When all the events above occur, $\mathsf{Rect}_{2n,n}$ contains a double- horizontal crossing. ThusSince $\epsilon$ is a universal constant and $C_\mathsf {cnt}$ and $C_\mathsf {wb}$ only depend on $C_v$, the above provides the desired bound. $\quad \square$

### Crossing rectangles in mixed boundary conditions

We give two statements that are crucial in the proof of Theorem 4.1. They are crossing probability estimates similar to those of Proposition 3.10. What is essential here is that they are in finite domains with mixed boundary conditions.

#### Proposition 4.13

For $C \ge 3$ there exists $\delta = \delta (C) > 0$ such that, for all $n\ge 1$,

#### Corollary 4.14

For all $C_h \ge 3$ and $C_v \ge 1$ there exists $\delta = \delta (C_h,C_v)> 0$ such that, for all $n\ge 1$,

Corollary 4.14 is referred to in [18] as the “pushing” lemma; it is an essential result in establishing the dichotomy of Corollary 4.2.

The results stated above mimic the structure of the original RSW theory: the first result serves as an input (such as self-duality in critical bond percolation on ${\mathbb {Z}}^2$ or as Corollary 3.9 for the weaker RSW statement of Proposition 3.10), the second states that horizontal crossings may be extended to longer rectangles. The latter follows from the former in a fairly standard way using Proposition 4.7. For clarity, we will avoid using Proposition 4.7 in the proof of Proposition 4.13. We start with the proof of the proposition; the proof of the corollary may be found at the end of the section.

#### Proof of Proposition 4.13

Fix $C\ge 3$. We will proceed by contradiction and will assume that4.22for some constant $\delta > 0$ that we will choose later. It will be obvious that the choice of $\delta$ only depends on *C*.

Write $\mathsf{Cyl}$ for $\mathsf{Cyl}_{Cn,5n}$ and . The cylinder is split into five strips of height *n*: $\mathsf{Strip}_i = [-Cn - 2, Cn + 2] \times [(i-1)n,in]$ for $i =1,\dots , 5$.

**Fig. 14:**
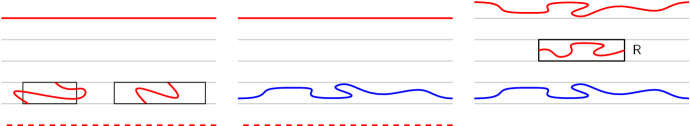
*Left:* A vertical double-crossing of red plus of $\mathsf{Strip}_2$ contains either a vertical crossing of a $6n\times n$ rectangle or a horizontal one of a $4n\times n$ rectangle. *Middle:* The absence of a vertical double-crossing of $\mathsf{Strip}_2$ of constant red spin implies the existence of a double-circuit of constant blue spin. Conditionally on it, we study the probability that ${\mathsf {R}}$ contains a double crossing of red-plus spins. *Right:* The symmetric cylinder ${{\widetilde{\mathsf{Cyl}}}}$. The probability that ${\mathsf {R}}$ is crossed (horizontally or vertically) by a double red-plus path is higher in the right image than in the middle

The proof of the proposition is based on two claims that we state and prove below. The whole argument is summarised in Figure 14. $\quad \square$

#### Claim 4.15

Write  for the event that there exists a double- path winding around $\mathsf{Cyl}$ and contained in $\mathsf{Strip}_2$. Assuming $\delta$ is small enough,

#### Proof

The same argument as in the proof of Lemma 3.8 shows that either $\mathsf{Strip}_2$ is crossed vertically by double-path of constant red spins, or it contains a horizontal double-circuit (winding around the cylinder) of constant blue spins. Thus4.23where  is defined similarly to  and  is the event that $\mathsf{Strip}_2$ contains a path of double- with one endpoint on its bottom and one on its top.

If  occurs, then at least one of the rectangles $[ kn,(k+6)n] \times [2n,3n]$ with $-C \le k < C$ is crossed vertically by a double- path, or one of the rectangles $[ kn,(k+4)n] \times [2n,3n]$ with $-C \le k < C$ is crossed horizontally by a double- path. Due to our assumptions (4.22) and to the monotonicity with respect to boundary conditions, all of the crossing events above occur with probabilities at most $\delta$. Thus,The same argument applies to double- crossings and we findFor this second inequality, the monotonicity of boundary conditions was not used, but rather the invariance of $\mu$ under vertical reflection composed with red spin flip.

Assume now that $\delta \le \frac{1}{16 C}$. Then the first two terms of (4.23) sum up to at least 1/2. Moreover, $\mu$ is invariant under blue spin flip, hence these two terms are equal. In conclusion, each is larger than 1/4. $\quad \square$

#### Claim 4.16

Let $${\mathsf {R}}= [-2n,2n] \times [3n,4n]$$. Then

#### Proof

If  occurs, let $\Gamma$ be the lowest circuit as in its definition. Let $\gamma$ be a possible realisation for $\Gamma$ and let ${{\overline{\gamma }}}$ be the reflection of $\gamma$ with respect to the horizontal line ${\mathbb {R}}\times \{7n/2\}$. Then ${{\overline{\gamma }}}$ lies entirely above ${\mathbb {R}}\times \{5n\}$, hence above the top of $\mathsf{Cyl}$. It will be useful to view $\gamma$ and ${{\overline{\gamma }}}$ as drawn on the infinite vertical cylinder  $\mathsf{Cyl}_\infty := ({\mathbb {R}}/ (2Cn+4){\mathbb {Z}}) \times {\mathbb {R}}$, of whom $\mathsf{Cyl}$ is a subset.

Let ${{\widetilde{\mathsf{Cyl}}}}$ be the cylinder contained between $\gamma$ and ${{\overline{\gamma }}}$ and let $\textsf {Top}$ be the top boundary of $\mathsf{Cyl}$ (it may be seen as a horizontal circuit in $\widetilde{\mathsf{Cyl}}$). Let  be the measure on ${{\widetilde{\mathsf{Cyl}}}}$ with boundary conditions  on ${{\overline{\gamma }}}$ and  on $\gamma$. Precisely,  is the uniform measure on pairs of coherent spin configuration  $(\sigma _r, \sigma _b)$ on ${\widetilde{\mathsf{Cyl}}}$ with the property that all faces adjacent to $\gamma$ have blue spin  and all faces adjacent to ${{\overline{\gamma }}}$ have .

Then, both the red-spin and blue-spin marginal of  have the FKG property. We sketch the proof of this fact next. Embed ${{\widetilde{\mathsf{Cyl}}}}$ in the plane in the same way that $\mathsf{Cyl}_{m,n}$ was embedded in Figure 8; call ${\mathscr {D}}$ the planar graph thus obtained. The measure  is equal to that on ${\mathscr {D}}$ with the faces adjacent to $\gamma$ conditioned to have blue spin  and those adjacent to ${{\overline{\gamma }}}$ to have red spin . By Corollary 2.10, this conditioned measure does satisfy the FKG inequality for both the blue and red-spin marginals.

Moreover, the boundary conditions of  satisfy the following Spatial Markov property for measures on $\mathsf{Cyl}_\infty$. Let $A_r$ be the set of faces that are either below $\gamma$, above $\overline{\gamma }$, or below ${{\overline{\gamma }}}$ but adjacent to it. Similarly, let $A_b$ be the set of faces that are above ${{\overline{\gamma }}}$, below $\gamma$, or above $\gamma$ but adjacent to it. Then, for any red spin configuration $\xi _r$ with the property that all faces adjacent to ${{\overline{\gamma }}}$ have spin  and any blue spin configuration $\xi _b$ with the property that all faces adjacent to $\gamma$ have spin , we have  where the equality refers only to the restriction on ${{\widetilde{\mathsf{Cyl}}}}$. This fact may be proved exactly as Theorem 2.3 and we do not give further details.

In addition, the Spatial Markov property for boundary conditions  holds under . Thus, using the FKG property for , we find4.24where  stands for the event that all faces adjacent to the top of $\mathsf{Cyl}$ have red spin . The same holds for .

Finally, let us mention that  is invariant under reflection with respect to the horizontal line ${\mathbb {R}}\times \{7n/2\}$ composed with colour inversion. This transformation maps  onto  and  onto . Now, due to Lemma 3.8, ${\mathsf {R}}$ contains either a horizontal double-crossing of constant red spin, or a vertical one of constant blue spin. Thus,4.25Define the boundary conditions  on ${{\widetilde{\mathsf{Cyl}}}}$ in the same way as . Then  is obtained from  by flipping the sign of all red spins. Using the same argument as in Corollary 2.12*(ii)*, it may be shown that the red spin marginal of  dominates that of . In particular,The same holds for vertical crossings. Insert the above in (4.25) and use (4.24), to findIn conclusionwhere the sum is over all possible realisations $\gamma$ of $\Gamma$. $\quad \square$

Finally, let us finish the proof of Proposition 4.13. For $\delta > 0$ small enough for Claim 4.15 to hold, using Claims 4.15 and 4.16, we findHowever, by our assumption (4.22), the left-hand side is bounded above by $2\delta$. This leads to a contradiction if $\delta$ is chosen smaller than 1/16. $\quad \square$

#### Proof of Corollary 4.14

Fix $C_h,C_v$ and *n* as in the statement. We may assume *n* larger than some constant depending on $C_v$ and $C_h$; the inequality for smaller values may be satisfied by altering the value of $\delta (C_v,C_h)$.

Apply Proposition 4.13 to $N = \frac{C_v}{5} n$ and $C = \frac{C_h}{5C_v}$ to obtain that4.264.27for some $\delta > 0$ depending only on $C_h$ and $C_v$. If the second inequality occurs, then Proposition 4.7 implies thatThus, up to replacing $\delta$ by $\psi (\delta )$, we may suppose that (4.26) holds always. Then, by repeated applications of Corollary 4.6 we deduce that4.28for some $\delta _0 > 0$ depending only on $C_h$ and $C_v$.

A consequence of Lemma 4.3 and of the FKG property is that the red-spin marginal of  is dominated by that of  . Using this, and the fact that $N = \frac{C_v}{5} n$, we find4.29Define the rectangles $R_j = \mathsf{Rect}_{C_h n, (4/5)^j C_v n}$ and  $S_j = [-C_h n,C_h n] \times [\frac{3}{4} \cdot (\frac{4}{5})^{j} C_v n, (\frac{4}{5})^{j+1} C_v n]$. Then (4.29) applies to any of the rectangles $R_j$ with $j \ge 0$, and we find4.30for some $\delta _j > 0$ that depends on $C_h$, $C_v$ and *j*, but not on *n*.^3^

**Fig. 15:**
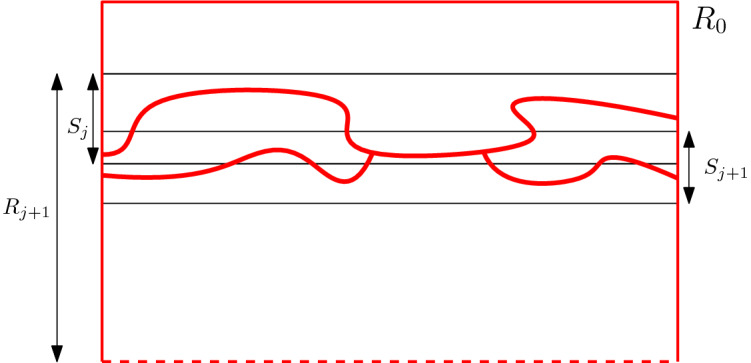
The rectangle $R_0 = \mathsf{Rect}_{C_h n ,C_v n}$ with  boundary conditions on the top and lateral sides and  on the bottom. If  occurs, the measure under $\Gamma$ dominates  and  is more likely to occur than in $R_{j+1}$

When  occurs for some $j \ge 0$, there exists a double- path contained in $S_j \subset R_{j+1}$, connecting the left and right side of $R_0$. Any such path separates the top of $R_0$ from its bottom. Let $\Gamma$ be the highest such path and $\textsf {Under}(\Gamma )$ be the set of faces of  $R_0$ that are separated from the top of $R_0$ by $\Gamma$. Then $\Gamma$ is measurable with respect to the spins above and adjacent to $\Gamma$. For any possible realisation $\gamma$ of $\Gamma$, due to the Spatial Markov property, the red-spin marginal of  restricted to $\textsf {Under}(\gamma )$ stochastically dominates that of . It follows that4.31This may appear surprising, as it is not always the case that $S_{j+1} \subset \textsf {Under}(\gamma )$. Let us explain briefly why (4.31) is nevertheless true. Couple the red-spin marginals of  and  in an increasing fashion (this is possible do to the stochastic domination of the former by the latter). Then, if $(\sigma _r, {{\tilde{\sigma }}}_r)$ is a sample of this coupling, ${{\tilde{\sigma }}}_r$ is equal to  for all faces adjacent to $\gamma$ and is greater of equal to $\sigma _r$ for the faces of $\textsf {Under}(\gamma )$. If $\sigma _r$ is such that  occurs, then  as well. See Figure 15 for an illustration.

Summing over all possible values of $\gamma$, we findIterating this for $j < J := \lfloor \log _{5/4} C_v \rfloor$, we findNotice that $S_J$ is included in $\mathsf{Rect}_{C_h n,n}$, hence the above implies thatThe right-hand side of the above is a positive constant depending only on $C_h$ and $C_v$, and the proof is complete. $\quad \square$

### Proof of dichotomy theorem (Theorem 4.1 and Corollary 4.2)

#### Proof of Theorem 4.1

**Fig. 16:**
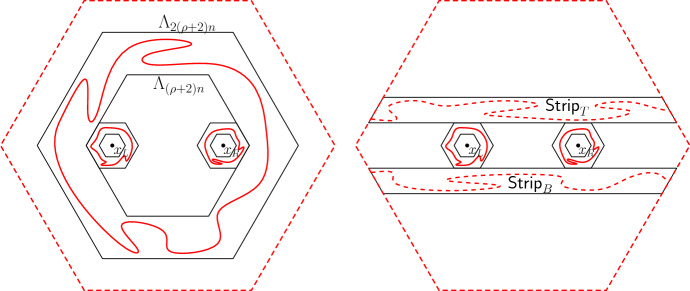
Left: Create  and  by first creating one double- circuit surrounding the whole of $\Lambda _{(\rho +2)n}$ (at a cost $\alpha _{(\rho +2)n}$), then creating two smaller circuits inside $\Lambda _{(\rho +2)n}$ which come at constant cost. **Right:** when  occurs, Corollary 4.14 allows us to create two long double- crossings in the strips above and below $\Lambda _{2n}(x_L)$

Fix *n* and let $\rho$ be some large constant (we will see below how to choose it). We will work in the domain ${\textsf {B}}:= \Lambda _{\rho (\rho +2) n}$, under the measure . The steps of the proof are described in Figure 16.

Let $x_L = (-\rho n,0)$ and $x_R = (\rho n,0)$. Write $\Lambda _k(x_L)$ for the ball of radius *k* centred at $x_L$, and use the same notation for $x_R$. Let  and  be the events that there exists a double- circuit in $\Lambda _{2n}(x_L) \setminus \Lambda _{n}(x_L)$ and $\Lambda _{2n}(x_R) \setminus \Lambda _{n}(x_R)$, respectively. Notice that both  and  depend only on the spins inside $\Lambda _{(\rho + 2)n}$. Recall that  is the event that there exists a double- circuit in $\Lambda _{\ell }$ that surrounds $\Lambda _{k}$.

By the monotonicity of boundary conditionsIn the second inequality we used the monotonicity of boundary conditions (Corollary 2.12) and the definition of $\alpha _{(\rho +2)n}$; the last inequality is due to the positive association of $\sigma _r$ under . It is a standard consequence of the comparison of boundary conditions and Corollary 4.14 that  for some constant $c_0>0$ that does not depend on *n*. In conclusion4.32We will now condition   on the event , and will construct double- circuits around $\Lambda _{2n}(x_L)$ and $\Lambda _{2n}(x_R)$. Using the Spatial Markov property, these will allow to bound the probability in (4.32) as a product of two probabilities $\alpha _n$.

When   occurs, write $\Xi _L$ for the innermost double- circuit as in the definition of . Then $\Xi _L$ is measurable in terms of the spins of the faces inside and adjacent to it. Define $\Xi _R$ in the same way. Let $\chi _L$ and $\chi _R$ be two possible realisations of $\Xi _L$ and $\Xi _R$, respectively. A straightforward variant of the Spatial Markov property (Theorem 2.3) states that the restriction of  to the faces of ${\textsf {B}}$ outside of $\chi _L$ and $\chi _R$ is independent of the values of the spins strictly inside $\chi _L$ and $\chi _R$. In particular, the restricted measure above is equal to , and its red-spin marginal satisfies the FKG inequality (see Corollary 2.10).

Consider the horizontal strip $\mathsf{Strip}_T = {\mathbb {R}}\times [\sqrt{3}n,2\sqrt{3}n]$; it sits above $\Lambda _{2n}(x_L)$ and $\Lambda _{2n}(x_R)$. Write  for the event that $\mathsf{Strip}_T\cap {\textsf {B}}$ is crossed horizontally by a double- path ($\mathsf{Strip}_T\cap {\textsf {B}}$ is not technically a rectangle, but we use the same notation). Then Corollary 4.14 (or rather its variant with  and  inverted) implies the existence of a constant $c_1> 0$ independent of *n*, $\chi _L$ and $\chi _R$ such that4.33Indeed, the red-spin marginal of  restricted to $\mathsf{Strip}_T \cap {\textsf {B}}$ is dominated by that in the rectangle $[-\rho (\rho +2) n, \rho (\rho +2) n]\times [\sqrt{3}n,\rho (\rho +2) n]$ with boundary conditions  on the bottom and  on all other sides.

The estimate (4.33) also holds for $\mathsf{Strip}_B$, the symmetric of $\mathsf{Strip}_T$ with respect to the horizontal axis ${\mathbb {R}}\times \{0\}$. Thus, by the FKG inequality,Summing over all possible values $\chi _L$ and $\chi _R$ of $\Xi _L$ and $\Xi _R$ and using (4.32), we findAs a consequenceWrite $\mathsf{D}$ for the domain that is the intersection of ${\textsf {B}}$ with the strip ${\mathbb {R}}\times [-2\sqrt{3} n,2\sqrt{3} n]$. Then, by conditioning on the highest and lowest double- crossings of $\mathsf{Strip}_T$ and $\mathsf{Strip}_B$, respectively, using the spatial Markov property and the monotonicity of boundary conditions, we find

**Fig. 17:**
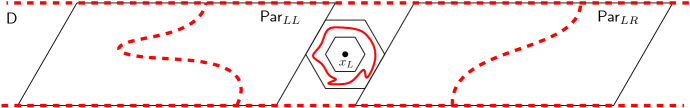
The rectangles $\mathsf{Par}_{LL}$ and $\mathsf{Par}_{LR}$ are to the left and right of $\Lambda _{2n}(x_L)$, respectively, and their top and bottom is part of the boundary of $\mathsf{D}$. Due to the  boundary conditions on $\mathsf{D}$, Lemma 4.5 applies to both these parallelograms. Thus, each is crossed vertically by a double- with uniform positive probability, independent of the configuration in the rest of $\mathsf{D}$

Now consider the parallelogram $\mathsf{Par}$ formed of the faces with centres at $k + \ell e^{i\pi /3}$ with $0 \le k \le 24n$ and $-4n \le \ell \le 4n$. Define its horizontal translates $\mathsf{Par}_{LL} = \mathsf{Par}- ((\rho + 26)n, 0)$, $\mathsf{Par}_{LR} = \mathsf{Par}- ((\rho -2)n, 0)$, $\mathsf{Par}_{RL} = \mathsf{Par}+ ((\rho -26)n, 0)$ and $\mathsf{Par}_{RR} = \mathsf{Par}+ ((\rho +2)n, 0)$. These are all contained in $\mathsf{D}$, touch its top and bottom and are left of $\Lambda _{2n}(x_L)$, right of $\Lambda _{2n}(x_L)$, left of $\Lambda _{2n}(x_R)$ and right of $\Lambda _{2n}(x_r)$, respectively. Let us assume that $\rho$ is large enough so that  $\mathsf{Par}_{LL}$ and $\mathsf{Par}_{LR}$ are included in $\Lambda _{\rho n}(x_L)$ ($\rho \ge 30$ suffices). Then $\mathsf{Par}_{RL}$ and $\mathsf{Par}_{RR}$ are included in $\Lambda _{\rho n}(x_R)$ and, in particular, are disjoint from the first two parallelograms.

Now observe that, due to Lemma 4.5 (applied with  and  exchanged)for some universal constant $c_2 >0$. Then, using Bayes ruleFinally, by conditioning on the left-most vertical double- crossing of $\mathsf{Par}_{LL}$ and the right-most of $\mathsf{Par}_{LR}$, and using the monotonicity of boundary conditions, it may be shown that the restriction of  to $\Lambda _{2n}(x_L)$ is dominated by that of . Moreover, due to the Spatial Markov property, this is true even when conditioning on the spins to the right of $\mathsf{Par}_{LR}$. The same procedure may be applied to  for the measure in $\Lambda _{2n}(x_R)$. Notice that the areas that determine the restriction of  to $\Lambda _{2n}(x_L)$ and $\Lambda _{2n}(x_R)$ are disjoint. Thus, the restriction of  to $\Lambda _{2n}(x_L)\cap \Lambda _{2n}(x_R)$ is dominated by the independent product of  and . In conclusion,The last two displayed equations yield the desired conclusion. $\quad \square$

#### Proof of Corollary 4.2

Let $\rho ,C$ be the constants of Theorem 4.1. Suppose that $\inf _n \alpha _n =0$. Let $n_0$ be such that $\alpha _{n_0} \le \frac{1}{2C}$. Then a simple induction involving (4.1) implies that $\alpha _{(\rho +2)^k n_0} \le \frac{1}{C} 2^{-2^k}$ for all $k \ge 0$. This implies the stated inequality for $c < \log 2 / \log (\rho +2)$ and *C* chosen accordingly.

## Conclusions

In this section we prove Theorems 1.2 and 1.3. To this end, we first resolve the dichotomy stated in Corollary 4.2 and then transfer the results from the spin representation to the loop *O*(2) model.

### Excluding stretched-exponential decay

The goal of this section is to show that the case *(ii)* of Corollary 4.2 is incoherent with Theorem 3.2. Once it is established that case *(i)* holds, it is fairly standard to deduce Theorem 1.2; this is done in Section 5.2.

#### Proposition 5.1

Case *(i)* of Corollary 4.2 occurs. That is,  for some fixed constant $\rho > 2$.

The constant $\rho$ and the ratio between the inner and outer radii of the annulus above may actually be chosen arbitrarily, as we prove below. Other variants referring to rectangle crossings may also be formulated.

#### Corollary 5.2

For any $$a > 1$$,

The lower bound on *n* in the infimum above is to ensure that the annulus is thick enough to allow the existence of a double- circuit. We start by proving the corollary, based on Proposition 5.1. The remainder of the section is then dedicated to proving Proposition 5.1.

#### Proof

This is a standard application of Proposition 5.1, the FKG property and the monotonicity of boundary conditions.

Fix $a > 1$ and let $b = (1+ a)/2$. We may limit ourselves to values of *n* larger than some threshold depending on *a*; smaller values of *n* only add strictly positive numbers to the set whose infimum we are considering.

**Fig. 18:**
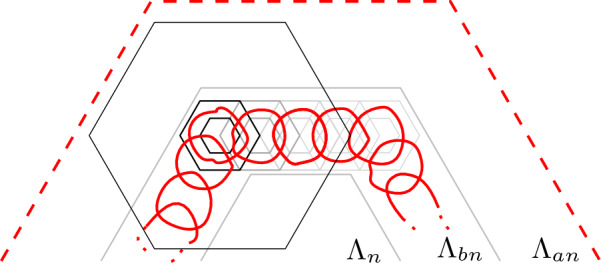
The small annuli $\text {Ann}_1,\dots ,\text {Ann}_K$ placed inside $\Lambda _{bn} \setminus \Lambda _n$ have inner radius *m* and outer radius 2*m*. They are such that the balls of radius $\rho m$ around each of their centres are contained in $\Lambda _{an}$. If they all contain double- circuits, then these form a circuit around $\Lambda _n$, contained in $\Lambda _{bn} \subset \Lambda _{an}$

Recall that $\rho$ is fixed by Theorem 4.1. Let $m = \lfloor \min \{ \frac{a-b}{\rho }; \frac{b-1}{4}\}\cdot n\rfloor$, and suppose that *n* is large enough so that $m \ge 2$. Then there exists a number $K = K(a,\rho )$, not depending on *m* or *n* such that one may place *K* translates $\text {Ann}_1,\dots , \text {Ann}_K$ of the annulus $\Lambda _{2 m } \setminus \Lambda _m$ inside $\Lambda _{bn} \setminus \Lambda _n$ in such a way that, if all of them contain a circuit of double-, then  occurs. See Figure 18 for an example.

Since $m \rho < (a -b)n$, all faces at distance $\rho m$ from each $\text {Ann}_j$ are contained in $\Lambda _{an}$. It follows from the FKG inequality and the monotonicity of boundary conditions thatwhere  is a strictly positive constant due to Proposition 5.1. Since the ultimate lower bound above does not depend on *n*, the proof is complete. $\quad \square$

We now turn to proving Proposition 5.1. We will proceed by contradiction. Fix $\rho > 2$ given by Theorem 4.1 and recall that . Will assume that case *(ii)* of Corollary 4.2 occurs, namely that there exist constants $c,C>0$ and $n_0 \ge 1$ such that

ExpDec $$\begin{aligned} \alpha _n \le C e^{-n^c} \qquad \text { for all }n = (\rho +2)^k n_0\text { with }k\in {\mathbb {N}}. \end{aligned}$$

We start by proving a series of results based on (ExpDec). All constants below depend implicitly on the values of $n_0$, $\rho$, *c* and *C* of (ExpDec).

#### Lemma 5.3

Under assumption (ExpDec), for any $\kappa \ge 2$ there exists $C_1 = C_1 (\kappa ) > 0$ such that5.1

#### Proof

**Fig. 19:**
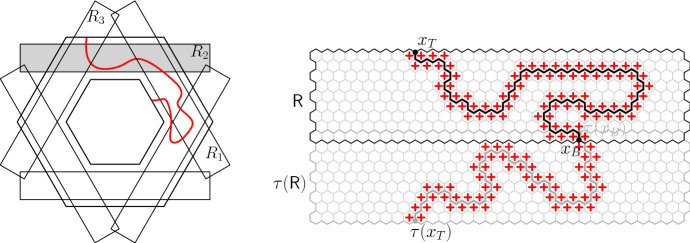
*Left:* One may place six copies of ${\mathsf {R}}$ around $\Lambda _n$ so that, if  occurs, then at least one of them is crossed in the short direction (here the crossed copy is shaded). *Right:* When $x_T$ is connected to $x_B$ in ${\mathsf {R}}$ and $\tau (x_T)$ is connected to $\tau (x_B)$ in $\tau ({\mathsf {R}})$, then $\tau (x_T)$ is connected to $x_T$ in $\tau ({\mathsf {R}})\cup {\mathsf {R}}$

Fix $\kappa \ge 2$ and *n* arbitrary. Let ${\mathsf {R}}:= \mathsf{Rect}_{2n, n/2}$. The annulus $\Lambda _{2n}\setminus \Lambda _n$ may be covered by six translations and rotation $R_1,\dots , R_6$ of ${\mathsf {R}}$ in such a way that, if  occurs, then at least one of $R_1,\dots , R_6$ is crossed in the short direction by a double- path (see Figure 19). For $1 \le i \le 6$, write  for the appropriate rotation and translation of . Then, using the union bound and the monotonicity of boundary conditions, we deduce that5.2Henceforth we aim to prove a stretched-exponential upper bound for .

Let $x_B$ and $x_T$ to be the points of the bottom and top, respectively, of ${\mathsf {R}}$ that are most probable under  to be connected by a double- path contained in ${\mathsf {R}}$. Then5.3since there are $16n^2$ potential pairs of points $(x_L,x_R)$. Let $\tau$ be the reflection with respect to the horizontal line ${\mathbb {R}}\times \{0\}$. Then we also have5.4If the events of (5.3) and (5.4) occur simultaneously, then $x_T$ and $\tau (x_T)$ are connected inside ${\mathsf {R}}\cup \tau ({\mathsf {R}}) = [-2n,2n] \times [-n/2,n/2]$ (see Figure 19). Thus, by the FKG inequality,Using the above, the FKG inequality again and the monotonicity of boundary conditions, we find,5.5Indeed, a vertical crossing of $[-2n,2n] \times [-8n,8n]$ may be obtained by intersecting sixteen translates of the event . The box has been increased to $\Lambda _{(\kappa + 10) n}$ so that all of these events occur in rectangles with distance to the boundary greater than $(\kappa + 2) n$.

**Fig. 20:**
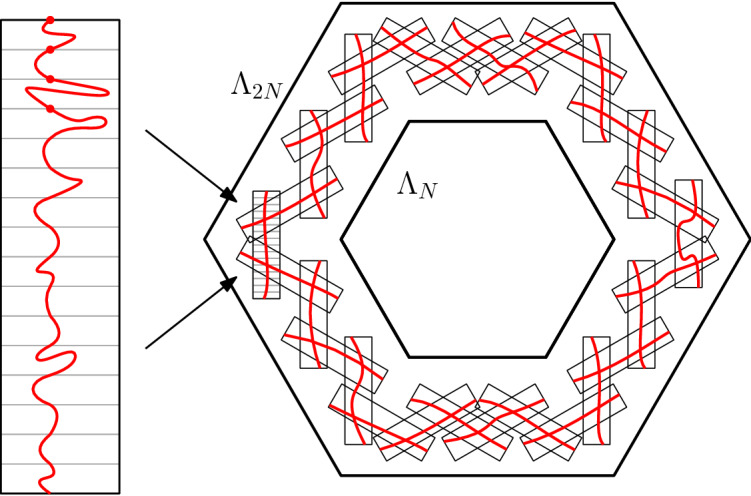
Combining *K* vertical crossings of (rotations and translations of) $[-2n,2n] \times [-8n,8n]$ produces a circuit in $\Lambda _{2N} \setminus \Lambda _N$. Each such vertical crossing is constructed from vertical crossings between $\tau (x_T)$ and $x_T$ in sixteen copies of ${\mathsf {R}}\cup \tau ({\mathsf {R}})$

Recall the fixed values $\rho >2$ and $n_0$ of (ExpDec). Let *k* be minimal such that, for $N := (\rho +2)^k n_0$, one has

$$\begin{aligned} N/n \ge \max \big \{16, \tfrac{\kappa + 10}{\rho -2}\big \}. \end{aligned}$$

By the minimality of *k*, we have $N \le c_0 n$ for some constant $c_0$ depending on $\rho$, $n_0$ and $\kappa$ only. Then, there exists a constant $K = K(\rho , n_0, \kappa )$, that depends on $\rho$, $n_0$ and $\kappa$ but not on *n* or on the resulting value of *N*, such that one may construct a circuit in $\Lambda _{2N} \setminus \Lambda _{N}$ by combining at most *K* vertical crossings of translates of $[-2n,2n] \times [-8n,8n]$ and rotations by $2\pi /3$ and $4\pi /3$ of this rectange, all contained in $\Lambda _{2N}$ (see Figure 20). Due to the choice of *N*, the faces at distance $(\kappa + 10)n$ from any of these rectangles are all contained in $\Lambda _{\rho N}$. Thus, by the monotonicity of boundary conditions and the FKG inequality,Due to (ExpDec), this impliesfor constants $c_1,c_2 > 0$ depending only on $\kappa$, $\rho$ and $n_0$. Finally, from (5.2) we deduce thatThis implies (5.1) with $C_1$ chosen small enough to absorb the multiplicative factor. $\quad \square$

#### Lemma 5.4

Under assumption (ExpDec), for any $\kappa \ge 2$ there exists $C_2 = C_2 (\kappa ) > 0$ such that5.6

#### Proof

Fix $\kappa \ge 2$ and let $C_2$ be some small constant to be fixed below (it will be obvious that the bound on $C_2$ depends only on $\kappa$). It suffices to prove the statement for *n* large enough; small values may be incorporated by adjusting $C_2$.

Suppose by contradiction that there exists $n \ge 1$ (large) such that  From now on *n* is fixed and it is crucial that we use the assumption above only for this particular value of *n*.

Due to the monotonicity of boundary conditions, we deduce thatfor any domain ${\mathscr {D}}$ containing $\Lambda _{\kappa n}$. Suppose now that $C_2$ is chosen smaller than $C_1(4\kappa )/2$, with $C_1(4\kappa )$ given by Lemma 5.3. Then, assuming *n* is above some threshold (which we will do from now on), we have5.7Due to the two displays above, and to the monotonicity of boundary conditions,for any domain ${\mathscr {D}}$ with $\Lambda _{\kappa n} \subset {\mathscr {D}}\subset \Lambda _{4\kappa n}$.

As in Lemma 3.6 , the absence of a double- or double- connection between $\Lambda _n$ and $\Lambda _{2n}^c$ implies that at least one of  and  occurs (see also Remark 3.7). Under , the blue spins are interchangeable, and we deduce that5.8for any domain ${\mathscr {D}}$ with $\Lambda _{\kappa n} \subset {\mathscr {D}}\subset \Lambda _{4\kappa n}$.

Next we work in the domain $\Lambda _{3\kappa n}$. Place translations $\text {Ann}_1,\dots , \text {Ann}_K$ of the annulus $\Lambda _{2n} \setminus \Lambda _n$ around the outside of $\Lambda _{\kappa n}$ as in Figure 18 so that, if all of them contain double- circuits, then there exists a double- circuit in $\Lambda _{(\kappa +2)n}$ surrounding $\Lambda _{\kappa n}$. As discussed in the proof of Corollary 5.2, this procedure employs a number *K* of translates that only depends on $\kappa$, not on *n*. Thus5.9The second inequality is due to the FKG property of blue spins under  (see Remark 2.11*(i)* with reversed colours). The last inequality is a consequence of (5.8). Indeed, if $\Lambda _{3\kappa n}$ is translated by the translation that sends $\text {Ann}_k$ to $\Lambda _{2n} \setminus \Lambda _n$, then it contains $\Lambda _{\kappa n}$ and is contained in $\Lambda _{4\kappa n}$, hence (5.8) applies to it.

When  occurs, write $\Gamma$ for the exterior most double- circuit in $\Lambda _{(\kappa + 2)n}$ that surrounds $\Lambda _{\kappa n}$. Let $\gamma$ be a possible realisation of $\Gamma$. Due to the Spatial Markov property, the measure  restricted to the interior $\textsf {Int}(\gamma )$ of $\gamma$ is simply . Then (5.8) with inverted colours applies to the domain $\textsf {Int}(\gamma )$, and we findAveraging the above over all possible values $\gamma$ taken by $\Gamma$ and using (5.9), we findThis contradicts (5.1) provided that $C_2$ is small enough (any $C_2 <C_1(3\kappa ) / (K+1)$ suffices) and *n* is large enough. $\quad \square$

#### Lemma 5.5

Under assumption (ExpDec), for any $\kappa \ge 2$ there exists $C_3 = C_3 (\kappa ) > 0$ such that5.10As a consequence, for any $\epsilon > 0$, there exists $n_1 \ge 1$ such that5.11

#### Proof

Fix some $\kappa \ge 2$. Let us first prove that5.12for all *n* and some fixed constant $C_4 > 0$. Notice that we are aiming to show that a thin rectangle is crossed in the long (vertical) direction with very high probability. Heuristically, Lemma 5.4 says that such rectangles are crossed with high probability in the short (i.e. horizontal) direction. To pass from crossing in the short direction to crossings in the long direction, we will use the same argument as in the proof of (5.5). However, since this argument applies to small probabilities rather than large ones, we will use it for the model dual to double- connections.

Recall the notation $\mathrm {dm}(\sigma _r)$ for the set of edges of ${\mathbb {H}}$ with spin  on either side. Let $\mathrm {dm}(\sigma _r)^*$ be the dual of  $\mathrm {dm}(\sigma _r)$; it is a percolation configuration on ${\mathbb {T}}$, with edges open if at least one of their endpoints is a face of spin . Then  fails if and only if $\mathsf{Rect}_{n/16, n/2}$ is crossed horizontally by a path in $\mathrm {dm}(\sigma _r)^*$. The same holds with $\mathsf{Rect}_{2n, n/2}$ instead of $\mathsf{Rect}_{n/16, n/2}$. Moreover, $\mathrm {dm}(\sigma _r)^*$ is increasing in $\sigma _r$, hence satisfies the FKG inequality under .

The same strategy as in the proof of Lemma 5.3 applies here, namely choosing the points on the left and right sides of $\mathsf{Rect}_{n/16, n/2}$ that are most likely to be connected in $\mathrm {dm}(\sigma _r)^*$, using horizontal reflection, the FKG inequality and monotonicity of boundary conditions, we find thatConsider now the copies $R_1,\dots , R_6$ of $\mathsf{Rect}_{2n, n/2}$ placed around $\Lambda _n$ as in Figure 19. Then, due to the FKG inequality and the comparison between boundary conditionsUsing the upper bound (5.6) for the LHS, we obtain (5.12) with an adjusted value $C_4 > 0$.

Let us now prove (5.10). There exists some fixed constant *K* such that one may place *K* translations and rotations by $2\pi /3$ and $4\pi /3$ of $\mathsf{Rect}_{n/16, n/2}$ around 0 in such a way that, if they are all crossed in the long direction by a double- path, then  occurs (look at Figure 20 for inspiration). Using again the FKG inequality and the monotonicity of boundary conditions, we findInserting  (5.12) in the above proves (5.10).

We move on to proving (5.11). For $n_1 \ge 1$, using the monotonicity of boundary conditions and the fact that , we findwhere $C_3$ is given by (5.10) for $\kappa = 4$. The right-hand side may be rendered as close to one as desired by taking $n_1$ sufficiently large. $\quad \square$

We are ready now to prove Proposition 5.1 and thus resolve the dichotomy stated in Corollary 4.2. Below we show that assumption (ExpDec) contradicts (3.1), hence it is false.

#### Proof of Proposition 5.1.

Suppose (ExpDec) occurs. Fix $n_1$ large enough so thatRecall from Theorem 3.1 that $\mu _{{\mathbb {H}}}$ is invariant under red spin flip, whenceThen, the intersection of the two events above occurs with probability at least 1/2. In particular, for any $j \ge 0$,Notice that  implies the occurrence of the the translate of  by $(2^{j}n_1,0)$. By the monotonicity of boundary conditions we deduce thatThis contradicts (5.1) for *j* large enough, and (ExpDec) fails. In other words, case *(i)* of Corollary 4.2 holds. $\quad \square$

### Proof of Theorem 1.2

In this section we show how the statements about the spin representation proven in previous sections imply Theorem 1.2.

#### Proof of Theorem 1.2.

***(i)*** Recall from Proposition 2.1 that for any domain ${\mathscr {D}}$, the measure ${\mathbb {P}}_{{\mathscr {D}}}$ is obtained from  by considering the edges separating faces of different blue or red spin.

If $({\mathscr {D}}_n)_{n\ge 1}$ is a sequence of increasing domains with $\bigcup _{n\ge 1} {\mathscr {D}}_n = {\mathbb {H}}$, Theorem 3.2 states that  converges to $\mu _{\mathbb {H}}$. As a consequence ${\mathbb {P}}_{{\mathscr {D}}_n}$ converges to the measure ${\mathbb {P}}_{\mathbb {H}}$ obtained from $\mu _{\mathbb {H}}$ by the same procedure that produces ${\mathbb {P}}_{\mathscr {D}}$ from .

***(ii)*** By Corollary 3.11, there exists ${\mathbb {P}}_{\mathbb {H}}$-a.s. no infinite path in $\omega _r$. Indeed, the existence of such a path is contradictory with the existence of infinitely many -circuits surrounding 0; the latter event was shown to occur $\mu _{\mathbb {H}}$-a.s. The statement extends to blue paths by symmetry.

Alternatively, one may see the proof of Theorem 3.2, where the absence of infinite paths was proved.

***(iii)*** The ergodicity and rotation invariance of ${\mathbb {P}}_{\mathbb {H}}$ follow from the corresponding properties of $\mu _{\mathbb {H}}$, which were obtained in Theorem 3.1.

***(iv)*** We start with the lower bound. Fix ${\mathscr {D}}$ a finite domain containing $\Lambda _{n}$ for some *n*, or simply ${\mathscr {D}}= {\mathbb {H}}$. The procedure that generates ${\mathbb {P}}_{{\mathscr {D}}}$ from  is such thatThe second and third inequalities are due to the monotonicity of boundary conditions. The last term is bounded uniformly away from 0 by Corollary 5.2.

Let us now prove the upper bound. Fix a finite domain ${\mathscr {D}}$ and set $n = \text {dist}(0,{\mathscr {D}}^c)$. Fix some $\rho <1$ close enough to 1; we will see below how $\rho$ needs to be chosen and that it does not depend on *n* or ${\mathscr {D}}$. Let $\mathsf {Loop}^2$ be the event that there exist at least two loops in ${\mathscr {D}}$ that surround $\Lambda _{\rho n}$. Let $\mathsf {Loop}^{2}(\text {red,blue})$ be the event that $\mathsf {Loop}^2$ occurs and that the outermost loop surrounding $\Lambda _{\rho n}$ is blue, while the second outermost is red. Then ${\mathbb {P}}_{{\mathscr {D}}}[\mathsf {Loop}^{2}(\text {red,blue})] =\frac{1}{4} {\mathbb {P}}_{\mathscr {D}}(\mathsf {Loop}^{2})$.

Let us consider for a moment spin configurations $(\sigma _r,\sigma _b)$ chosen according to  that correspond to loop configurations in $\mathsf {Loop}^{2}(\text {red,blue})$. The outermost blue loop corresponds to a double- circuit in ${\mathscr {D}}\setminus \Lambda _{\rho n}$. Indeed, as any blue loop, it is either a double- circuit or a double- circuit. Since there is no red loop separating it from $\partial _E{\mathscr {D}}$, its spin is the same as that of $\partial _\mathrm {in}{\mathscr {D}}$, namely . The second outermost loop, the red one, induces a simple- circuit that surrounds $\Lambda _{\rho n}$ and is contained inside the double- circuit above.

Coming back to general configurations $(\sigma _r,\sigma _b)$ on ${\mathscr {D}}$, let $\Xi$ be the outermost double- circuit surrounding $\Lambda _{\rho n}$; if no such circuit exists, set $\Xi =\emptyset$. Let $\textsf {Int}(\Xi )$ be the domain delimited by $\Xi$. Due to the Spatial Markov property and a standard exploration argument, the measure inside $\Xi$ is .

By the discussion above, if $\mathsf {Loop}^{2}(\text {red,blue})$ occurs, then $\Xi \ne \emptyset$ and there exists a simple- circuit contained in $\textsf {Int}(\Xi )$ and surrounding $\Lambda _{\rho n}$ (this is not an equality of events; generally the latter event contains strictly the former). Thus5.13where the sum is over all realisations $\chi \ne \emptyset$ of $\Xi$. The event  above refers to the existence of simple- circuit contained in $\textsf {Int}(\chi )$ and surrounding $\Lambda _{\rho n}$.

Due to the monotonicity of boundary conditions  for any $\chi$ in the sum above. In conclusion5.14Let *u* be a point where $\partial _E \Lambda _{n}$ intersects $\partial _E {\mathscr {D}}$ (such a point exists due to the choice of *n*). By considering the intersection of the annulus $u + \Lambda _n \setminus \Lambda _{(1-\rho )n}$ centred at *u* with ${\mathscr {D}}$, and using the monotonicity of boundary conditions, we find that5.15Indeed, the trace on ${\mathscr {D}}$ of any configuration $\sigma _r$ on ${\mathbb {H}}$ that contains a simple- circuit in the annulus $u + \Lambda _n \setminus \Lambda _{(1-\rho )n}$ does not belong to . The above conclusion follows by the domination .

It is a standard consequence of Corollary 5.2 that $\rho <1$ may be chosen so that  for all *n* larger than some fixed threshold. Then, by (5.14) and (5.15), ${\mathbb {P}}_{\mathscr {D}}(\mathsf {Loop}^{2}) \le \frac{1}{2}$ for all *n* above this threshold, as required. Smaller values of *n* may be incorporated by altering the constant *c*.

***(v)*** Fix a domain ${\mathscr {D}}$; we will prove the results for ${\mathbb {E}}_{\mathscr {D}}$; the same proof applied for ${\mathbb {E}}_{\mathbb {H}}$. First we show the lower bound. Set $K = \lfloor \log _2 \mathrm {dist}(0,{\mathscr {D}}^c)\rfloor$ and for $k = 1,\dots , K$, let $\mathsf{Circ}^2(k)$ be the event that there exists a double- circuit and a double- circuit in $\Lambda _{2^{k+1}}$ surrounding $\Lambda _{2^k}$. By the same reasoning as that used to prove *(iv)* above,5.16for some constant $c>0$ independent of *k*. As a consequence

$$\begin{aligned} {\mathbb {E}}_{{\mathscr {D}}}(\#\{ 1\le k < K-1\, :\, \mathsf{Circ}^2(k) \text { occurs}\}) \ge c\, (K-1) \ge c \,\big (\log _2 \mathrm {dist}(0,{\mathscr {D}}^c) -2\big ). \end{aligned}$$

Now observe that each event $\mathsf{Circ}^2(k)$ that occurs induces a loop in ${\mathscr {D}}$ that surrounds 0. This provides the desired lower bound, after alteration of the constant *c*.

We turn to the upper bound. Let $\Gamma _1,\dots , \Gamma _{N_{\mathscr {D}}}$ be the loops of $\omega$ surrounding 0, ordered from outermost to innermost, when $\omega$ is chosen according to ${\mathbb {P}}_{\mathscr {D}}$. Set $d_j = \text {dist}(0,\Gamma _j)$ for $j =1,\dots , N_{\mathscr {D}}$.

The loop measure inside $\Gamma _j$, conditionally on $\Gamma _1,\dots , \Gamma _j$, and more generally on the whole configuration outside $\Gamma _j$, is simply ${\mathbb {P}}_{\textsf {Int}(\Gamma _{j})}$. Applying (1.2) we find that

5.17 $$\begin{aligned} {\mathbb {P}}_{\mathscr {D}}( d_{j+2} < \rho \, d_j \,|\, \Gamma _j \text { and }\omega \text { outside }\Gamma _j) > c. \end{aligned}$$

From the above, it is standard to conclude that ${\mathbb {E}}_{\mathscr {D}}(N_{\mathscr {D}}) \le C \log n$ for some constant *C* depending on *c* and $\rho$ only. We sketch this below.

Let $T_k = \min \{ j\le N_{\mathscr {D}}:\, d_j < \rho ^k\cdot \mathrm {dist}(0,{\mathscr {D}}^c)\}$ for $k =1,\dots ,K$ where $K = \lceil \log _{1/\rho }\mathrm {dist}(0,{\mathscr {D}}^c)\rceil$. Formally $T_K = N_{\mathscr {D}}$ and $T_0 = 0$. Then (5.17) implies that each $T_{j+1} - T_j$ may be bounded by a random variable $2 G_j$, where $G_j$ has a geometric distribution of parameter $c > 0$. Then ${\mathbb {E}}_{\mathscr {D}}(N_{\mathscr {D}}) \le \sum _{j=1}^K 2{\mathbb {E}}(G_j) = \frac{2 K }{c}$, as required.

Finally, (5.16) also applies to ${\mathbb {P}}_{\mathbb {H}}$ instead of ${\mathbb {P}}_{\mathscr {D}}$, and directly implies that

$$\begin{aligned} \liminf _{K \rightarrow \infty } \frac{\#\{ 1\le k < K \, :\, \mathsf{Circ}^2(k) \text { occurs}\}}{\log K} > 0. \end{aligned}$$

Thus, there are indeed infinitely many loops surrounding the origin ${\mathbb {P}}_{\mathbb {H}}$-a.s.. $\quad \square$

### Proof of Theorem 1.3

Finally we prove Theorem 1.3. It may be worth mentioning that the proof below may be adapted to circumvent the use of the results of Section 4. Indeed, the non-quantitative delocalisation result of Theorem 3.2 suffices.

#### Proof of Theorem 1.3

From the construction of ${\mathbb {P}}_{\mathbb {H}}$ as limit of finite-volume measures, it is immediate that it is a Gibbs measure. The rest of the proof is dedicated to showing it is the only one.

Let $\eta$ be a Gibbs measure. For any configuration $\omega$ chosen according to $\eta$, colour each loop of $\omega$ independently in red or blue; colour each infinite path in red. Write $\omega _r$ and $\omega _b$ for the obtained red and blue configurations, respectively. Extend $\eta$ to incorporate this additional randomness.

Additionally, associate to $(\omega _r,\omega _b)$ a pair of spin configurations $(\sigma _r,\sigma _b)$ obtained by choosing the spins at 0  uniformly, then assign spins to all other faces with the constraint that two faces have distinct red spin (and blue spin, respectively) if and only if they are separated by an edge of $\omega _r$, and $\omega _b$, respectively.

Thus $\eta$ is both a law on pairs of red and blue loop configurations, as well as a law on pairs of red and blue spin configurations. We call the latter the double-spin representation of $\eta$.

Let us show that the red-spin marginal of $\eta$ is equal to $\nu _{\mathbb {H}}$. Notice that (DLR) implies that the double-spin representation of $\eta$ has the spatial Markov property in that, for any domain ${\mathscr {D}}$, the restriction of $\eta$ to $\textsf {Int}({\mathscr {D}})$ conditionally on the double-spin configuration outside $\textsf {Int}({\mathscr {D}})$ is measurable in terms of the double-spin configuration on $\partial _\mathrm {in}{\mathscr {D}}$.

Fix $\epsilon > 0$ and $n\ge 1$. Let $N > n$ be chosen so that5.18for any event *A* that depends only on spins in $\Lambda _n$. Now let $M > N$ be such that

5.19 $$\begin{aligned} \eta (\text {there exists a finite loop visiting }\Lambda _N\text { and }\Lambda _M^c) \le \epsilon . \end{aligned}$$

Since the event above is limited to finite loops, it is always possible to find such a value *M*. Write *B*(*N*, *M*) for the event in the probability above.

For a configuration $\xi \notin B(N,M)$, let ${\mathscr {D}}(\xi )$ be the domain obtained from $\Lambda _M$ by removing the interior of all loops intersecting its boundary. Then $\Lambda _N \subset {\mathscr {D}}(\xi )$. Let $X_1,\dots , X_{2k}$ be the points on $\partial _E {\mathscr {D}}(\xi )$ that belong to infinite paths in $\xi \setminus {\mathscr {D}}(\xi )$ and which have neighbours inside ${\mathscr {D}}(\xi )$, ordered in counter-clockwise order, starting from some arbitrary point. For $\xi \in B(N,M)$, set ${\mathscr {D}}(\xi ) = \emptyset$.

Let $D,x_1,\dots , x_{2k}$ be a possible realisation of ${\mathscr {D}}(\xi ),X_1,\dots , X_{2k}$ with $D \ne \emptyset$. Then (DLR) describes the restriction ${\mathbb {P}}_{D}^{x_1,\dots ,x_{2k}}$ of $\eta (.\,|\, {\mathscr {D}}(\omega ) = D \text { and } X_1,\dots ,X_{2k} = x_1,\dots , x_{2k})$ to *D* as

$$\begin{aligned} {\mathbb {P}}_{D}^{x_1,\dots ,x_{2k}}(\omega ) =\tfrac{1}{Z} \,2^{\#\ell (\omega )}{\mathbf {1}}_{\{\text {the odd vertices of }\omega \text { are }x_1,\dots ,x_{2k}\}}, \qquad \text {for all }\omega \in \{0,1\}^{E({\mathscr {D}})}, \end{aligned}$$

where $\ell (\omega )$ is the number of loops of $\omega$ entirely contained in *D*. Indeed, for any $\xi$ and $\omega$ as above, the loops of the configuration $\omega \cup (\xi \cap D^c)$ that intersect *D* are all contained in *D*. Additionally *D* also contains *k* segments of infinite paths.

Let us describe the conditional measure above for spin configurations. Since no finite loop intersects $\partial _E D$, the blue spins on $\partial _\mathrm {in}D$ are all identical, either  or . The red spins along $\partial _\mathrm {in}D$ switch from  to  and vice-versa at every point $x_i$ due to the infinite (red) paths. Write  for the spin measure on *D*   on $\partial _\mathrm {in}D$,  on the segments of $\partial _\mathrm {in}D$ between $x_i$ and $x_{i+1}$ with *i* odd, and  on all other parts of $\partial _\mathrm {in}D$. To be precise, this is the uniform measure on coherent configurations  that have the values above on $\partial _\mathrm {in}D$. Define ,  and , similarly; these are the push-forward of  via $(\sigma _r,\sigma _b) \mapsto (-\sigma _r,\sigma _b)$, $(\sigma _r,\sigma _b) \mapsto (\sigma _r,-\sigma _b)$ and $(\sigma _r,\sigma _b) \mapsto (-\sigma _r,-\sigma _b)$, respectively. Then, due to the uniform choice of the spins at 0,Due to Corollary 2.10 *(iii)*, the red spin marginals of the mesures , ,  and  all satisfy the FKG inequality. In particular, since $\Lambda _N \subset D$, their restrictions to $\Lambda _n$ are all dominated by . In conclusion we find that, for any increasing event *A* depending only on the red spins inside $\Lambda _n$,The last inequality is due to (5.18) and (5.19). Recall that the choice of $\epsilon$ is arbitrary, hence $\eta (A) \le \mu _{{\mathbb {H}}}(A)$ for all events *A* as above.

The same argument may be performed with  replaced by , and yields that for any decreasing event *B* depending only on the red spins inside $\Lambda _n$ (that is the complement of an increasing event), $\eta (B) \le \mu _{{\mathbb {H}}}(B)$. Thus, $\eta (A) = \mu _{{\mathbb {H}}}(A)$ for all increasing (and decreasing) events that only depend on the red spins in a finite region. The monotone class theorem allows to conclude that the red spin marginals of $\eta$ and $\mu _{\mathbb {H}}$ are equal.

Now, given the red spin marginal of $\mu _{\mathbb {H}}$, the blue spins are obtained by awarding uniform blue spins to the clusters of $\theta (\sigma _r)$. The same holds for $\eta$, since 0 is surrounded $\eta$-a.s. by infinitely many disjoint clusters of $\theta (\sigma _r)$. As a consequence $\eta = \mu _{\mathbb {H}}$. $\quad \square$

### RSW theorem for height functions

We finish the paper with a RSW result for the uniform Lipschitz functions model. Recall the notation of Section 1.1. When considering Lipschitz functions on a domain containing $\Lambda _{2n}$, write $\mathsf{Circ}_{\ge k}(n)$ for the event that there exists a closed face-path contained in $\Lambda _{2n}$, surrounding $\Lambda _n$ and formed entirely of faces for which the function is larger than *k*.

#### Theorem 5.6

For any $k \ge 1$ there exists $c(k) > 0$ such that for all *n* large enough and any domain ${\mathscr {D}}$ containing $\Lambda _{2n}$.

$$\begin{aligned} \pi _{\mathscr {D}}(\mathsf{Circ}_{\ge k}(n)) \ge c(k). \end{aligned}$$

#### Proof

Fix $k \ge 1$. Let $n \ge 1$ be a large integer and ${\mathscr {D}}$ a domain containing $\Lambda _{2n}$. Recall from Propositions 1.4 and 2.1 that the loop representation of a height function chosen according to $\pi _{\mathscr {D}}$ has law ${\mathbb {P}}_{\mathscr {D}}$, and its spin representation has law .

Write *H* for the event that there exist $k+1$ closed edge-paths $\gamma _1,\dots , \gamma _{k+1}$ in $\Lambda _{2n}$ that surround $\Lambda _n$, that are numbered from outer-most to inner-most, and such that $\gamma _j$ is a double- path if *j* is odd and a double- path if *j* is even. By repeated applications of Corollary 5.2, there exists a constant $c(k) > 0$ independent of *n* or ${\mathscr {D}}$ such that .

When *H* occurs, there exist at least *k* loops in the loop representation that are contained in $\Lambda _{2n} \setminus \Lambda _n$ and surround $\Lambda _n$. Write ${{\tilde{H}}}$ for set of loop configurations which contain at least *k* such loops, and denote by $\Gamma _1,\dots , \Gamma _k$ the outermost *k* loops as above. Recall that, in order to obtain the height function $\Phi$ from the loop configuration $\omega$ chosen according to ${\mathbb {P}}_{\mathscr {D}}$, loops need to be oriented uniformly, and that the orientation of each loop dictates whether the height inside the loop is larger or smaller than the one outside. By symmetry, conditionally on any loop configuration $\omega \in {{\tilde{H}}}$, with probability at least 1/2 the height of the faces outside and adjacent to $\Gamma _1$ is at least 0. Moreover, independently of the above, all paths $\Gamma _1,\dots , \Gamma _k$ are oriented clockwise with probability $2^{-k}$. When both of the above occur, the height of the faces inside and adjacent to $\Gamma _k$ is at least *k*. Thuswhich is the desired conclusion. $\quad \square$

## Footnotes

1 Here, due to the atypical boundary conditions, the configurations $\omega _r$ and $\omega _b$ are allowed to induce odd degrees for vertices on the boundary of ${\mathscr {D}}$. Then they are formed of disjoint loops and paths with endpoints on the boundary of ${\mathscr {D}}$.

2 Due to the choice of *a*, *b*, *c* and *d*, all such faces are on $\partial _\mathrm {out}{\mathscr {D}}$; their spins have no influence on the measure induced in ${\mathscr {D}}$.

3 We may actually restrict ourselves to *n* and *j* such that the rectangles $R_j$ and $S_j$ do not degenerate below the mesh size. Indeed, (4.30) will only be used with $j \le \log _{5/4} C_v$ and *n* may be assumed large enough.
